# The importance of stool DNA methylation in colorectal cancer diagnosis: A meta-analysis

**DOI:** 10.1371/journal.pone.0200735

**Published:** 2018-07-19

**Authors:** Afsaneh Mojtabanezhad Shariatpanahi, Maryam Yassi, Mehdi Nouraie, Amirhossein Sahebkar, Fatemeh Varshoee Tabrizi, Mohammad Amin Kerachian

**Affiliations:** 1 Cancer Genetics Research Unit, Reza Radiotherapy and Oncology Center, Mashhad, Iran; 2 Department of Medicine, University of Pittsburgh, Pittsburgh, PA, United States of America; 3 Biotechnology Research Center, Pharmaceutical Technology Institute, Mashhad University of Medical Sciences, Mashhad, Iran; 4 Neurogenic Inflammation Research Center, Mashhad University of Medical Sciences, Mashhad, Iran; 5 School of Pharmacy, Mashhad University of Medical Sciences, Mashhad, Iran; 6 Medical Genetics Research Center, Mashhad University of Medical Sciences, Mashhad, Iran; 7 Department of Medical Genetics, Faculty of Medicine, Mashhad University of Medical Sciences, Mashhad, Iran; University of Bonn, Institute of Experimental Hematology and Transfusion Medicine, GERMANY

## Abstract

A large number of tumor-related methylated genes have been suggested to be of diagnostic and prognostic values for CRC when analyzed in patients' stool samples; however, reported sensitivities and specificities have been inconsistent and widely varied. This meta-analysis was conducted to assess the detection accuracy of stool DNA methylation assay in CRC, early stages of CRC (advanced adenoma, non-advanced adenomas) and hyperplastic polyps, separately. We searched MEDLINE, Web of Science, Scopus and Google Scholar databases until May 1, 2016. From 469 publications obtained in the initial literature search, 38 studies were included in the final analysis involving 4867 individuals. The true positive, false positive, true negative and false negative of a stool-based DNA methylation biomarker using all single-gene tests considering a certain gene; regardless of a specific gene were pooled and studied in different categories. The sensitivity of different genes in detecting different stages of CRC ranged from 0% to 100% and the specificities ranged from 73% to 100%. Our results elucidated that *SFRP1* and *SFRP2* methylation possessed promising accuracy for detection of not only CRC (DOR: 31.67; 95%CI, 12.31–81.49 and DOR: 35.36; 95%CI, 18.71–66.84, respectively) but also the early stages of cancer, adenoma (DOR: 19.72; 95%CI, 6.68–58.25 and DOR: 13.20; 95%CI, 6.01–28.00, respectively). Besides, *NDRG4* could be also considered as a significant diagnostic marker gene in CRC (DOR: 24.37; 95%CI, 10.11–58.73) and *VIM* in adenoma (DOR: 15.21; 95%CI, 2.72–85.10). In conclusion, stool DNA hypermethylation assay based on the candidate genes *SFRP1*, *SFRP2*, *NDRG4* and *VIM* could offer potential diagnostic value for CRC based on the findings of this meta-analysis.

## Introduction

Colorectal cancer (CRC) is the third most common malignancy and the fourth leading cause of cancer-related death in the world with more than half million deaths every year [1–3]. Recent advances in our understanding of CRC epigenetic aberration have led to the identification of potential clinical biomarkers for prognostic, diagnostic, and therapeutic monitoring of CRC [3]. Early detection of colon cancer through screening and removal of adenomatous polyps prevents cancerous transformation and lowers the incidence and mortality rates [4]. One of the main process causing the initiation of CRC and transformation of benign polyps to malignant tumors is the accumulation of a variety of genetic and epigenetic changes in colonic epithelium [3, 5].

Although colonoscopic screening remains the gold standard for CRC screening, this procedure is invasive and expensive, and suffers from poor patient compliance [6]. Hence, there is remarkable interest in the development of accurate noninvasive screening tests, among which stool-based tests (*e*.*g*. stool DNA analysis) have been particularly the subject of extensive research. Stool DNA test, provides several advantages over colonoscopy, such as ease of performance, low risk and its low cost [7]. Stool DNA test detects aberrant methylation and mutation in DNA released from cells that are constantly shed from cancerous or pre-cancerous lesions [8]. Previous studies have identified a set of DNA methylation biomarkers isolated from patients' stool as a user-friendly and cost-effective procedure for noninvasive screening and early detection of cancer with a high analytical sensitivity and stability superior to the guaiac-based fecal occult blood tests (g-FOBTs) [9–12]. Numerous tumor-related hypermethylated genes in the stool of CRC patients have been introduced with different sensitivity and specificity values for CRC [13] and a relatively unclear diagnostic performance in cancer.

Based on the above-mentioned points, this meta-analysis was conducted to assess the diagnostic performance of individual DNA hypermethylation genes in stool samples. We also aimed to find the best single genes for the diagnosis of colorectal cancerous and precancerous lesions.

## Materials and methods

### Literature search strategy

The meta-analysis was performed in accordance with the PRISMA 2009 guidelines [14]. We searched MEDLINE, Web of Science, Scopus and Google Scholar international databases until May 1, 2016. The keywords employed for literature retrieval were (Methylation/ Methylated/ Hypermethylation/ Hypermethylated) AND (Colorectal/ Colon/ Rectal/ "large intestine") AND (Stool/ Feces/ Fecal) AND (Sensitivity/ Specificity) AND (Tumor/ Cancer/ Polyp/ Carcinoma/ Adenocarcinoma/ Neoplas*/ Adenom*) AND NOT (Rat/ Mice/ Mouse). We contacted authors to obtain additional information when necessary.

### Inclusion and exclusion criteria

Two reviewers (M. A. and Y. M.) independently assessed all identified publications to determine their eligibility for inclusion in the study. Studies meeting the following criteria were included in the study: (1) employed colonofibroscopic or surgical pathology examination as the reference standard; (2) inclusion of a control group consisting of normal healthy individuals; (3) stool collection before any tumor removal and polypectomy; (4) all included studies used stool DNA hypermethylation tests as CRC screening tool; (5) provision of sufficient data for 2 × 2 table construction for each gene separately; (6) original articles; (7) full-length article published in English. Exclusion criteria were: (1) diagnoses of secondary or metastatic instead of primary colon cancer; (2) chronic inflammatory diseases mimicking malignancy (such as inflammatory bowel disease); (3) duplicate publication; (4) trials lacking appropriate informed consent; (5) studies without control or normal group; (6) studies with same population.

### Study selection and data extraction

All potential studies were reviewed thoroughly by 2 independent reviewers (M. A. and Y. M.) using a standardized form (S1 Table). Any disagreement was resolved by discussion until consensus was reached. The reviewers were not blind to the journal and author names, author affiliations, or year of publication, as this procedure has been shown to be unnecessary. In this meta-analysis, 2 × 2 tables were constructed from each gene in each cancer category in all studies for the true-positive (TP), false-negative (FN), and true-negative (TN) and false-positive (FP) values. All essential data and relevant information, including the name of the first author, year of publication, sample size, study design, subject demographics, pathology or colonoscopy reports of participants, targeted genes, and lab DNA methylation detection method of targeted genes and country of study population were extracted from the included studies.

### Quality assessment

The Revised Quality Assessment of Diagnostic Accuracy Studies (QUADAS)-2 tool was utilized for quality assessment for the included studies [15], which has been demonstrated to be efficient for quality assessment of diagnostic accuracy studies. This tool consists of 4 key domains that cover patient selection, index tests, reference standard, and flow of patients through the study and timing of the index tests and reference standard (flow and timing). The quality assessment was also performed by the independent reviewers and a third reviewer was consulted for any uncertainties. The quality of each item was characterized as low, high, or unclear.

### Statistical analysis

The outcomes of the meta-analysis were the diagnostic performance, denoted as sensitivity, specificity, the positive likelihood ratio (PLR), negative likelihood ratio (NLR), and diagnostic odds ratio (DOR) of single-gene tests. The summary receiver operating characteristic curve (SROC) displays the trade-off between sensitivity and specificity and represents a global summary of test performance. The PLR represents the value by which the odds of the disease increase when a test is positive, whereas the NLR shows the value by which the odds of the disease decrease when a test is negative [16].

Because random error and clinical or methodological heterogeneity can affect study results, heterogeneity among the studies was assessed by the Cochran Q and the I^2^ statistic. For the Q statistic, P < 0.10 was considered statistically significant for heterogeneity. For the I^2^ statistic, which indicates the percentage of the observed between-study variability due to heterogeneity rather than chance, the following ranges were used: no heterogeneity (I^2^  =  0%–25%), moderate heterogeneity (I^2^  =  25%–50%), large heterogeneity (I^2^  =  50%–75%), and extreme heterogeneity (I^2^  =  75%–100%). Q statistics (P < 0.1) or I^2^ statistic (I^2^ > 50%) were considered to indicate the existence heterogeneity between studies.

We pooled estimates for sensitivity, specificity, the PLR, NLR, DOR and SROC curve. We used the professional statistical software programs (Meta-DiSc 1.4, Ramón y Cajal Hospital in Madrid, Spain) [16]. All statistics were calculated and then combined using a random-effects model and 95%CI as effect measurements for our analysis.

In addition, publication bias was inspected using Egger Test to evaluate Funnel plots of the DOR against study standard error. Funnel plot was conducted using Metafor package in R software [17].

## Results

### Study selection

**Fig 1:**
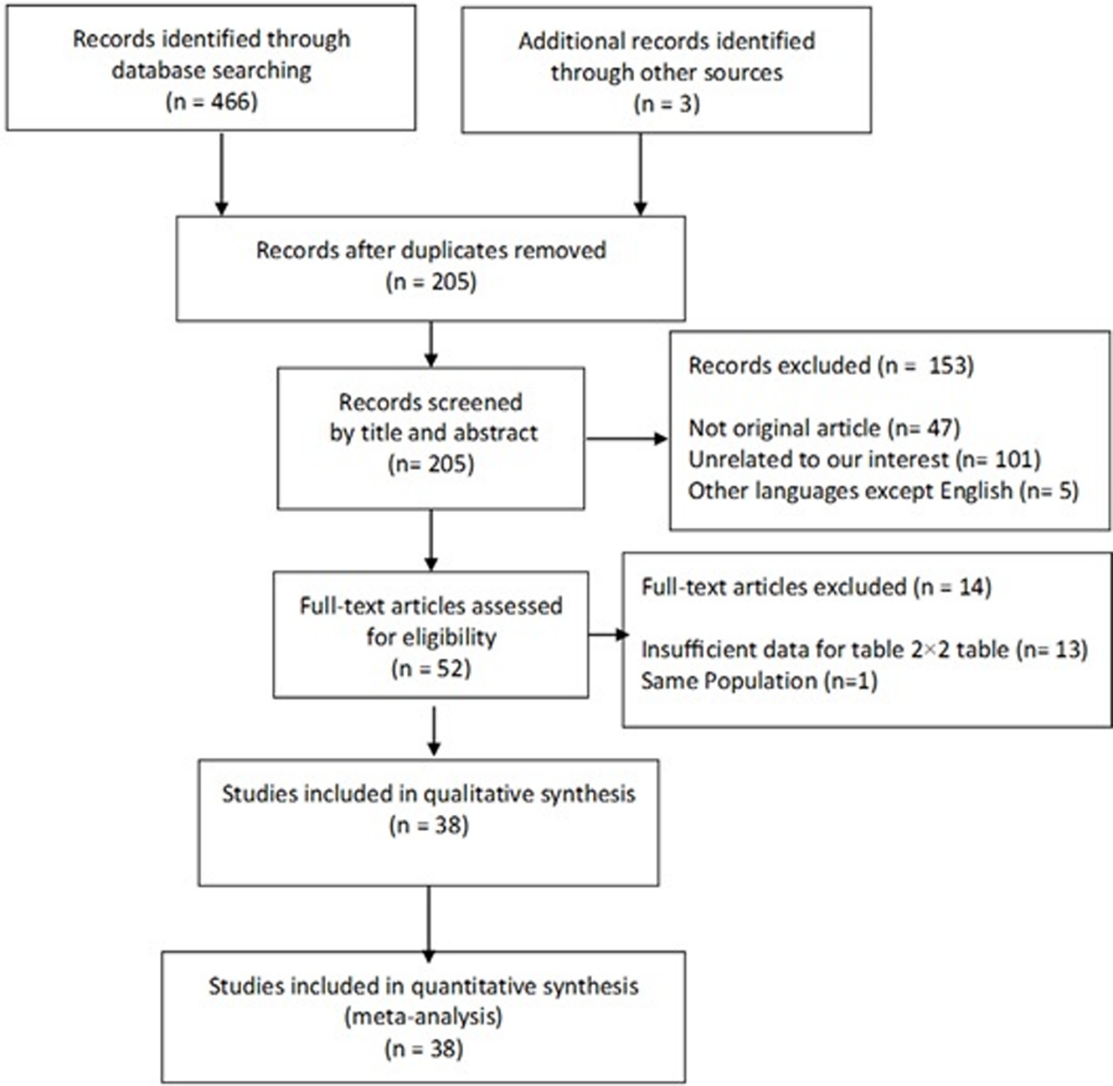
Flow diagram of study selection.

Of the 469 articles initially identified, in the next stage of assessment 205 duplicate publications were removed. One hundred fifty three were excluded by title and abstract. The remaining 52 studies were fully reviewed, of which 14 were excluded for not precisely meeting all the inclusion criteria. After carefully reading the texts, meta-analysis was performed on the final sample of 38 studies (Fig 1).

### Sample characteristics

The total number of participants in the studies was 4867, with the percentage of male patients and controls ranging from 26.6%-70% to 35%-75% and the percentage of female patients and controls from 30%-73.33% to 25%-65%. The patients and controls had a mean age of 61.60 ± 12.37 and 55.76 ± 12.5, respectively. Patients were classified into four categories including; CRC, total adenoma (TA, including advanced adenoma; AA and non-advanced adenoma; NAA), hyperplastic polyp (HP) and CRC plus adenoma (total patients; TP) named from 1 to 4, respectively. In TP category, hyperplastic polyps were not included since there is a scanty chance of malignancy. The numbers of patients in the four categories were 2005, 667, 154, and 2762 respectively. The total number of control groups were 1951. The sensitivity of a given assay for detecting CRC and polyps in stool samples varied across the studies; the ranges for sensitivity of categories 1 to 4, were 20%-94%, 0%-100%, 0%-50% and 0%100%, whereas their specificities were 77%-100%, 73%-100%, 73%-100% and 73%-100%, respectively. The population studied, targeted genes assessed, the targeted categories, the analysis methods to detect the hypermethylation of the targets genes and the sensitivity and specificity of each gene in a certain category of a given study until 2016 are shown in Table 1. Studies were conducted on four continents: Europe (n = 12; 1 in France, 3 in Spain, 4 in Netherland, 1 in Germany, 2 in Austria, 1 in Belgium), Asia (n = 20; 15 in China, 2 in Iran, 2 in South Korea, 1 in Japan), and North America (n = 4; all in the USA) and one multi-center study.

**Table 1:** Summary of basic characteristics and performance of studies included in meta-analysis.

Ref.	Study country	Detection method	Sample type: Number	Target Gene(s)	Study Group	TP	FN	TN	FP	Sensitivity* (%)	Specificity* (%)
Li, W.-h., et al. (2015) [18]	China	MSP	CRC: 89 Control: 30	*SNCA*	CRC/N	62	27	30	0	70	100
				*FBN1*	CRC/N	63	26	28	2	71	93
Xiao, W., et al. (2015) [19]	China	MSP	CRC: 87 Control: 16	*NDRG4*	CRC/N	66	21	14	2	76	89
Amiot, A., et al. (2014) [20]	France	qMSP	CRC, AA: 90 Control: 157	*Wif-1*	CRC+ AA/N	17	73	155	2	19	99
				*ALX4*	CRC+AA/N	10	80	155	2	11	99
				*VIM*	CRC+AA/N	29	61	157	0	33	100
He, C. G., et al. (2014) [11]	China	MSP	CRC: 61 AA: 27 Control: 20	*p33(ING1b)*	CRC/N	45	16	19	1	74	95
				*p33(ING1b)*	AA/N	17	10	19	1	63	95
Lu, H., et al. (2014) [21]	China	MSP	CRC: 56 Control: 40	*SFRP2*	CRC/ N	32	24	36	4	57	90
				*GATA4*	CRC/N	24	32	38	2	43	95
				*GATA5*	CRC/N	47	9	33	7	84	85
				*NDRG4*	CRC/N	16	40	39	1	28.5	97.5
				*VIM*	CRC/N	23	33	34	6	42	85
Xiao, Z., et al. (2014) [22]	China	MS_HRM	CRC: 40 AA: 36 Control: 57	*SFRP2*	CRC/N	35	5	52	5	87.5	91
				*VIM*	CRC/N	22	18	53	4	55	93
				*SFRP2*	AA/N	20	16	52	5	56	91
				*VIM*	AA/N	30	6	53	4	83	93
Zhang, H., et al. (2014) [10]	China	MSP	CRC: 48 AA: 15 NAA: 20 HP: 32 Control: 30	*SFRP2*	CRC/N	27	21	30	0	56	100
				*Wif-1*	CRC/N	29	19	29	1	60	97
				*SFRP2*	AA/N	9	6	30	0	60	100
				*Wif-1*	AA/N	8	7	29	1	53	97
				*SFRP2*	NAA/N	8	12	30	0	40	100
				*Wif-1*	NAA/N	7	13	29	1	35	97
				*SFRP2*	HP/N	4	28	30	0	12.5	100
				*Wif-1*	HP/N	6	26	29	1	19	97
Carmona, F. J., et al. (2013) [23]	Spain	Pyrosequencing	CRC: 68 Control: 39	*AGTR1*	CRC/N	14	54	37	2	21	95
				*WNT2*	CRC/N	21	31	38	1	40	97
				*SLIT2*	CRC/N	37	34	35	2	52	95
				*VIM*	CRC/N	18	15	19	3	55	86
				*SEPT9*	CRC/N	7	28	26	0	20	100
Guo, Q., et al. (2013) [13]	China	MSP	CRC: 75 Control: 30	*FBN1*	CRC/N	54	21	28	2	72	93
Zhang, H., et al. (2013) [24]	China	MSP	CRC: 96 Control: 30	*SPG20*	CRC/N	77	19	30	0	80	100
Bosch, L. J., et al. (2012) [25]	Netherland	qMSP	CRC: 65 AA: 19 Control: 101	*PHACTR3*	CRC/N	40	25	97	4	61.5	96
				*PHACTR3*	AA/N	6	13	97	4	31.5	96
Salehi, R., et al. (2012) [26]	Iran	MSP	CRC: 25 Control: 25	*SFRP1*	CRC/N	13	12	23	2	52	92
Zhang, J., et al. (2012) [12]	China	MSP	CRC: 60 A: 20 Control: 30	*TFPI2*	CRC/N	41	19	30	0	68	100
				*TFPI2*	A/N	7	13	30	0	35	100
Tang, D., et al. (2011) [27]	China	MSP	CRC: 169 AA: 63 Control: 30	*SFRP2*	CRC/N	142	27	28	2	84	93
				*SFRP2*	AA/N	29	34	28	2	46	93
Azuara, D., et al (2010) [28]	Spain	MS-MCA	CRC: 38 A: 40 Control: 20	*RARB2*	CRC/N	11	23	13	0	32	100
				*P16*	CRC/N	9	21	13	0	30	100
				*MGMT*	CRC/N	9	19	15	0	32	100
				*APC*	CRC/N	9	19	15	0	32	100
				*RARB2*	A/N	7	31	15	0	18	100
				*P16*	A/N	6	28	15	0	18	100
				*MGMT*	A/N	3	34	15	0	8	100
				*APC*	A/N	9	25	15	0	26	100
Baek, Y. H., et al. (2009) [29]	South Korea	MSP	CRC: 60 A: 52 Control: 37	*MLH1*	CRC/N	18	42	37	0	30	100
				*MGMT*	CRC/N	31	29	32	5	52	86
				*VIM*	CRC/N	23	37	37	0	38	100
				*MLH1*	A/N	6	46	37	0	11	100
				*MGMT*	A/N	19	33	32	5	36.5	86
				*VIM*	A/N	8	44	37	0	15	100
Chang, E., et al. (2009) [30]	South Korea	MSP	CRC: 30 A: 25 Control: 31	*ITGA4*	CRC/N	11	19	31	0	37	100
				*SFRP2*	CRC/N	18	12	31	0	60	100
				*P16*	CRC/N	12	18	30	1	40	97
				*ITGA4*	A/N	4	21	31	0	16	100
				*SFRP2*	A/N	11	14	31	0	44	100
				*P16*	A/N	6	19	30	1	24	97
Glöckner, S. C., et al. (2009) [31]	Netherland	qMSP	CRC: 84 A: 26 Control: 87	*TFPI2*	CRC/N	67	17	76	11	80	87
				*TFPI2*	A/N	7	19	76	11	27	87
Hellebrekers, D. M., et al. (2009) [32]	Netherland	qMSP	CRC: 75 Control: 75	*GATA4*	CRC/N	44	31	66	9	59	88
Mayor, R., et al. (2009) [33]	Spain	MS-MCA	CRC: 30 Control: 30	*EN1*	CRC/N	8	22	29	1	27	97
Melotte, V., et al. (2009) [34]	Netherland	qMSP	CRC: 75 Control: 75	*NDRG4*	CRC/N	42	33	72	3	56	96
Nagasaka, T., et al. (2009) [35]	Japan	Fluorescence Hi-SA	CRC: 84 AA: 27 NAA: 29 HP: 12 Control: 113	*RASSF2*	CRC/N	38	46	107	6	45	95
				*SFRP2*	CRC/N	53	31	104	9	63	92
				*RASSF2*	AA/N	6	20	107	6	22	95
				*SFRP2*	AA/N	10	17	104	9	37	92
				*RASSF2*	NAA/N	1	28	107	6	3.5	95
				*SFRP2*	NAA/N	8	21	104	9	28	92
				*RASSF2*	HP/N	6	6	107	6	50	95
				*SFRP2*	HP/N	6	6	104	9	50	92
Kim, M. S., et al. (2009) [36]	Belgium	qMSP	CRC: 89 A: 17 Control: 96	*OSMR*	CRC/N	35	54	92	4	39	96
				*SFRP1*	CRC/N	11	9	15	0	55	100
				*B4GALT1*	CRC/N	9	7	8	2	56	80
				*OSMR*	A/N	2	14	92	4	13	96
				*SFRP1*	A/N	5	12	15	0	29	100
Itzkowitz, S., et al. (2008) [37]	United States	MSP	CRC: 22 AA: 20 Control: 38	*VIM*	CRC/N	9	13	36	2	41	95
				*VIM*	AA/N	9	11	36	2	45	95
Li, M., et al. (2009) [38]	United States	Methyl-BEAMing	CRC: 42 AA: 6 Control: 241	*VIM*	CRC/N	34	8	198	43	81	82
				*VIM*	AA/N	6	0	198	43	100	82
Oberwalder, M., et al. (2008) [39]	Austria	Methyl Light	A: 13 HP: 6 Control: 6	*SFRP2*	CRC/N	6	7	6	0	46	100
				*SFRP2*	HP/N	2	4	6	0	33	100
Tang, D., et al. (2008) [40]	China	MSP	CRC: 39 AA: 19 NAA: 15 HP: 17 Control: 20	*SFRP1*	CRC/N	35	4	18	2	90	90
				*SFRP2*	CRC/N	32	7	19	1	82	95
				*SFRP1*	AA/N	14	5	18	2	74	90
				*SFRP2*	AA/N	13	6	19	1	68	95
				*SFRP1*	NAA/N	8	7	18	2	53	90
				*SFRP2*	NAA/N	6	9	19	1	40	95
				*SFRP1*	HP/N	6	11	18	2	35	90
				*SFRP2*	HP/N	5	12	19	1	29	95
Wang, D.-R. and D. Tang (2008) [41]	China	Methyl Light	CRC: 69 AA: 34 HP 26 Control: 30	*SFRP2*	CRC/N	60	9	28	2	87	94
				*SFRP2*	AA/N	21	13	28	2	62	94
				*SFRP2*	HP/N	11	15	28	2	42	94
Abbaszadegan, M. R., et al. (2007) [42]	Iran	MSP	CRC: 25 Control: 20	*P16*	CRC/N	5	20	20	0	20	100
Huang, Z.-H., et al. (2007) [43]	China	MSP	CRC: 52 A: 21 HP: 8 Control: 24	*SFRP2*	CRC/N	49	3	23	1	94	96
				*HPP1*	CRC/N	37	15	24	0	71	100
				*MGMT*	CRC/N	25	27	24	0	48	100
				*SFRP2*	A/N	11	10	23	1	52	96
				*HPP1*	A/N	12	9	24	0	57	100
				*MGMT*	A/N	6	15	24	0	29	100
				*SFRP2*	HP/N	3	5	23	1	37.5	96
				*HPP1*	HP/N	2	6	24	0	25	100
				*MGMT*	HP/N	1	7	24	0	12.5	100
				*SFRP2*	HP/N	3	5	23	1	37.5	96
				*HPP1*	HP/N	2	6	24	0	25	100
				*MGMT*	HP/N	1	7	24	0	12.5	100
Itzkowitz, S. H., et al. (2007) [44]	7 Center	MSP	CRC: 40 Control: 122	*VIM*	CRC/N	29	11	106	16	72.5	87
				*HLTF*	CRC/N	15	25	113	9	37.5	93
Leung, W. K., et al. (2007) [45]	China	MSP	CRC: 20 A: 25 HP: 5 Control: 30	*SFRP2*	CRC/N	6	14	28	2	30	93
				*MGMT*	CRC/N	4	16	30	0	20	100
				*MLH1*	CRC/N	4	16	30	0	20	100
				*HLTF*	CRC/N	4	16	29	1	20	97
				*ATM*	CRC/N	5	15	30	0	25	100
				*APC*	CRC/N	4	16	30	0	20	100
				*SFRP2*	A/N	3	22	28	2	12	93
				*MGMT*	A/N	3	22	30	0	12	100
				*MLH1*	A/N	3	22	30	0	12	100
				*HLTF*	A/N	5	20	29	1	20	97
				*ATM*	A/N	4	21	30	0	16	100
				*APC*	A/N	4	21	30	0	16	100
				*SFRP2*	HP/N	1	4	28	2	20	93
				*MGMT*	HP/N	0	5	30	0	0	100
				*MLH1*	HP/N	1	4	30	0	20	100
				*HLTF*	HP/N	1	4	29	1	20	97
				*ATM*	HP/N	1	4	30	0	20	100
				*APC*	HP/N	0	5	30	0	0	100
Zhang, W., et al. (2007) [46]	Germany	MSP	CRC: 29 A: 7 Control: 17	*SFRP1*	CRC/N	24	5	15	2	83	88
				*SFRP1*	A/N	7	0	15	2	100	88
Chen, W.-D., et al. (2005) [47]	United States	MSP	CRC: 94 A: 50 HP: 29 Control: 107	*VIM*	CRC/N	43	51	99	8	46	92.5
				*VIM*	A/N	6	44	99	8	12	92.5
				*VIM*	HP/N	6	23	99	8	21	92.5
Lenhard, K., et al. (2005) [48]	Germany	MSP	CRC: 26 A: 13 HP: 9 Control: 32	*HIC1*	CRC/N	11	15	32	0	42	100
				*HIC1*	A/N	4	9	32	0	31	100
				*HIC1*	HP/N	0	9	32	0	0	100
Petko, Z., et al. (2005) [49]	United States	MSP	A: 28 HP: 10 Control: 19	*MLH1*	A/N	0	28	17	2	0	90
				*MGMT*	A/N	14	14	13	5	50	73
				*CDKN2A (P16)*	A/N	9	18	16	3	33	84
				*MLH1*	HP/N	0	10	17	2	0	90
				*MGMT*	HP/N	3	7	13	5	30	73
				*CDKN2A (P16)*	HP/N	1	6	16	3	14	84
Müller, H. M., et al. (2004) [50]	Austria	Methyl Light	CRC: 23 Control: 26	*SFRP2*	CRC/N	19	4	20	6	83	77
Leung, W. K., et al. (2004) [51]	China	MSP	CRC: 20 Control: 20	*ATM*	CRC/N	5	15	20	0	25	100
				*APC*	CRC/N	4	16	20	0	20	100
				*MGMT*	CRC/N	4	16	20	0	20	100
				*MLH1*	CRC/N	4	16	20	0	20	100

MSP: methylation specific PCR, qMSP: quantitative methylation specific PCR, MS-HRM: methylation specific-high resolution melting, MS-MCA: methylation specific- melting curve analysis, Hi-SA: high sequence assay. CRC: colorectal cancer, A: adenoma, AA: advanced- adenoma, NAA: non-advanced adenoma, HP: hyperplastic polyp, N: normal/control FN: false negative, the number of cancerous lesions with negative diagnoses, FP: false positive, the number of non-cancerous lesions with positive diagnoses, TN: true negative, the number of non-cancerous lesions with negative diagnoses, TP: true positive, the number of cancerous lesions with positive diagnoses.

*Sensitivity (%) = TP/ (TP+FN) × 100% and specificity (%) = TN/ (TN+FP) × 100%.

### Performance of single-gene stool DNA methylation biomarker tests

The results of the analysis of pooled sensitivity, specificity, PLR, NLR, and SROC of a stool-based DNA methylation biomarker test using all single-gene tests regardless of a specific gene in 3 categories (CRC, TA and TP) are shown in Table 2 and (S1 File). The results showed the performance of single-gene stool DNA methylation tests in CRC (DOR: 18.54; 95%CI, 15.25–22.54) is higher than the early stages of cancer, TA (DOR: 8.79; 95%CI, 6.07–12.71).

**Table 2:** Performance of single-gene stool DNA methylation biomarker tests.

Groups	Sensitivity % (95%CI)	Specificity % (95%CI)	PLR (95%CI)	NLR (95%CI)	DOR (95%CI)	AUC
**CRC**	56.5 [55–58]	93 [92–94]	6.438 [5.629–7.362]	0.496 [0.448–0.549]	18.541 [15.250–22.542]	0.9033
**TA**	33 [30–35]	93 [92–94]	4.903 [3.858–6.232]	0.726 [0.669–0.788]	8.787 [6.073–12.714]	0.8838
**TP**	50 [48–51]	93 [92–94]	5.977 [5.151–6.934]	0.547 [0.500–0.598]	14.392 [11.720–17.673]	0.8814

CRC: colorectal cancer, TA: total adenoma TP: total patients, PLR: positive likelihood ratio, NLR: negative likelihood ratio, DOR: diagnosis odd ratio, AUC: area under the ROC curve

### Performance of a certain gene in single-gene stool-based DNA methylation biomarker tests

**Fig 2:**
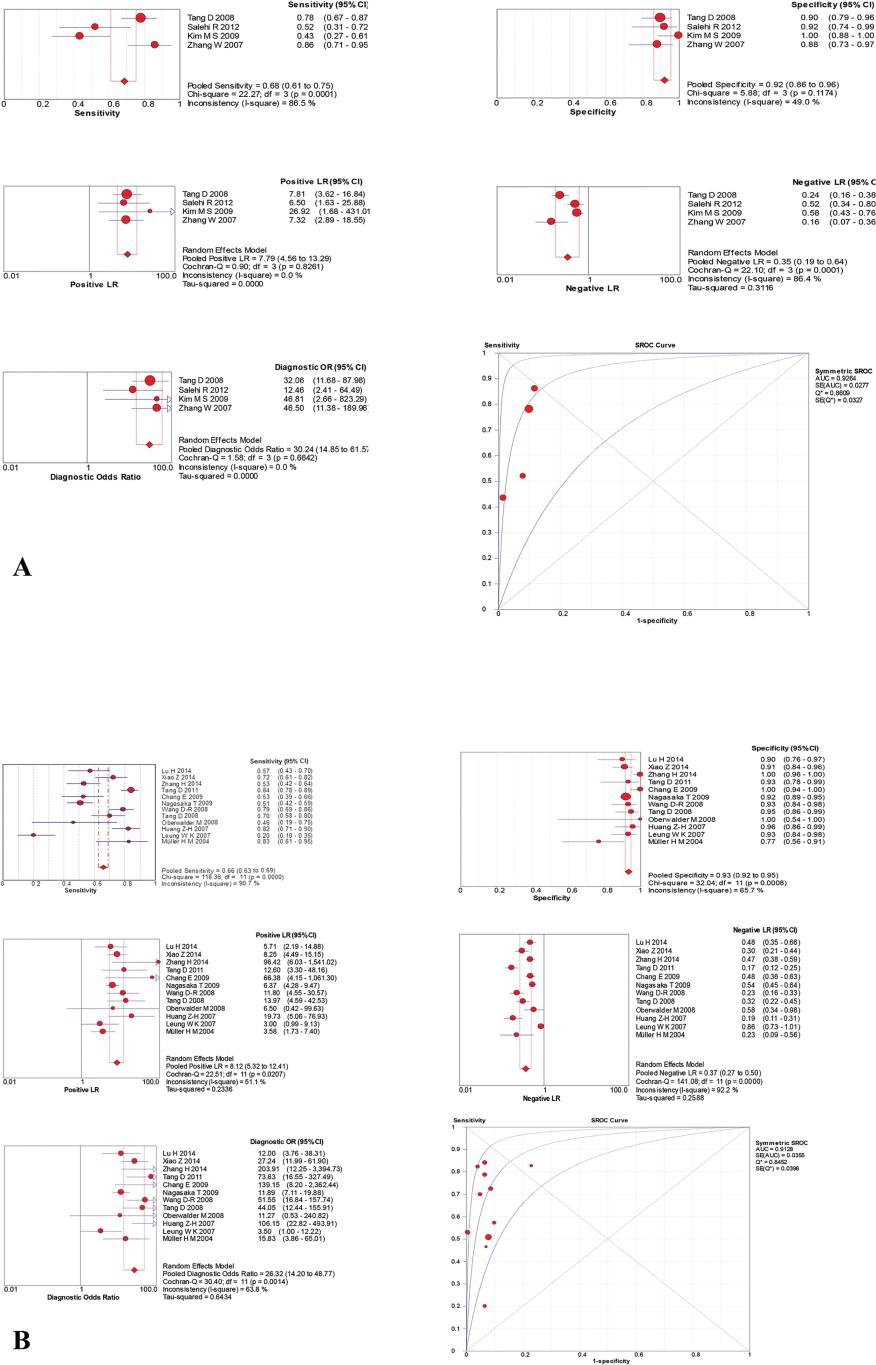
Summary estimates of SFRP1 and SFRP2. (A) Summary estimates of SFRP1. hypermethylation in stool samples used for TP (CRC+ Adenoma) diagnosis. Red circles represent each study that was included in the meta-analysis. The size of each study is indicated by the size of the red circle. Error bars indicate the 95% confidence interval (CI). Positive LR: positive likelihood ratio, Negative LR: negative likelihood ratio, Diagnostic OR: diagnosis odd ratio, SROC curves: summary receiver operating characteristic curve. (B) Summary estimates of SFRP2. hypermethylation in stool samples used for TP (CRC+ Adenoma) diagnosis. Red circles represent each study that was included in the meta-analysis. The size of each study is indicated by the size of the red circle. Error bars indicate the 95% confidence interval (CI). Positive LR: positive likelihood ratio, Negative LR: negative likelihood ratio, Diagnostic OR: diagnosis odd ratio, SROC curves: summary receiver operating characteristic.

We pooled estimates for sensitivity, specificity, PLR, NLR, DOR and the SROC of a stool-based DNA methylation biomarker test using all single-gene tests considering a certain gene in our 5 categories including CRC, AA, NAA, HP, TA, TP. The results of the analysis of pooled data from all genes which have been reported at least in three studies are shown in Table 3 and (S2 File). The results elucidated that Secreted Frizzled-Related Protein 1 (*SFRP1)* and Secreted Frizzled-Related Protein 2 (*SFRP2)* methylation possess the highest accuracy for detection of not only CRC (DOR: 31.67; 95%CI, 12.31–81.49 and DOR: 35.36; 95%CI, 18.71–66.84, respectively) but also the early stages of cancer, TA (DOR: 19.72; 95%CI, 6.68–58.25 and DOR: 13.20; 95%CI, 6.01–28.00, respectively) as illustrated in Fig 2. N-myc downstream regulated gene 4 (*NDRG4)* could be also considered as a significant diagnostic marker gene in CRC (DOR: 24.37; 95%CI, 10.11–58.73) and Vimentin (*VIM*) in adenoma (DOR: 15.21; 95%CI, 2.72–85.10).

**Table 3:** Performance of a certain gene in all single-gene stool-based DNA methylation biomarker tests included in this meta- analysis.

GENES	Number of studies	Sensitivity* % (95%CI)	Specificity* % (95%CI)	PLR (95%CI)	NLR (95%CI)	DOR (95%CI)	AUC
**CRC**							
*SFRP2*	12	74.5 [71–78]	93 [90–95 ]	7.917 [5.415–11.576]	0.294 [0.205–0.422]	35.362 [18.709–66.839]	0.9436
*VIM*	8	52 [47–57]	88 [85–90]	4.899 [3.932–6.104]	0.523 [0.424–0.644]	12.056 [7.885–18.432]	0.8686
*MGMT*	5	41 [33–48]	96 [91–99]	5.336 [2.576–11.054]	0.674 [0.563–0.807]	9.797 [4.127–23.258]	0.7234
*MLH1*	3	26 [18–36]	100 [96–100]	14.154 [2.755–72.724]	0.754 [0.671–0.848]	18.613 [3.415–101.45]	0.5653
*HLTF*	3	71 [52–85]	74 [68–80]	2.567 [1.835–3.590]	0.427 [0.261–0.699]	7.890 [3.444–18.074]	0.8094
*SFRP1*	4	73.5 [64–81]	92 [84–97]	7.938 [3.775–16.689]	0.301 [0.156–0.578]	31.670 [12.307–81.495]	0.9437
*APC*	3	25 [15–37]	100 [94–100]	10.771 [2.097–55.317]	0.771 [0.671–0.887]	14.13 [2.55–78.27]	0.8565
*P16*	3	31 [21–41]	98 [92–100]	10.409 [2.566–42.218]	0.731 [0.626–0.852]	15.056 [3.409–66.498]	0.9639
*NDRG4*	3	57 [50–64]	95 [90–98]	10.027 [4.585–21.929]	0.464 [0.269–0.802]	24.374 [10.115–58.730]	0.9061
**AA**							
*SFRP2*	5	56 [47–64]	93 [90–96]	6.592 [3.941–11.024]	0.486 [0.374–0.632]	14.379 [6.873–30.083]	0.8912
*VIM*	3	73 [60–83]	85 [81–89]	7.722 [2.519–23.671]	0.269 [0.073–0.990]	38.881 [14.523–104.09]	0.9549
**NAA**							
*SFRP2*	3	34 [23–48]	94 [89–97]	5.422 [1.849–15.900]	0.710 [0.595–0.847]	7.915 [2.328–26.906]	0.0438
**HP**							
*SFRP2*	7	30 [22–40]	94 [90–97]	6.116 [3.370–11.099]	0.740 [0.617–0.888]	9.488 [4.368–20.608]	0.8646
*MGMT*	3	22 [6–48]	88 [74–96]	1.803 [0.310–10.476]	0.880 [0.682–1.136]	2.046 [0.302–13.868]	-
**TA**							
*SFRP2*	8	45 [39–51]	94 [91–96]	6.905 [3.767–12.657]	0.575 [0.447–0.738]	13.200 [6.009–28.996]	0.8882
*VIM*	5	36 [29–44]	88 [85–90]	5.466 [2.689–11.112]	0.571 [0.350–0.932]	15.211 [2.719–85.105]	0.9470
*MGMT*	5	28 [21–35]	92 [86–96]	2.507 [1.417–4.438]	0.823 [0.712–0.951]	3.829 [1.811–8.097]	0.6224
*MLH1*	3	82 [48–98]	47 [39–54]	1.682 [0.954–2.964]	0.467 [0.012–17.634]	2.380 [0.140–40.403]	0.4167
*SFRP1*	3	59 [45–71]	92 [83–97]	6.754 [3.297–13.837]	0.451 [0.190–1.074]	19.720 [6.676–58.253]	0.9221
*P16*	3	24 [16–35]	94 [85–98]	3.124 [1.203–8.114]	0.815 [0.715–0.928]	4.303 [1.393–13.291]	0.4720
**TP**							
*SFRP2*	12	66 [63–69]	93 [92–95]	8.121 [5.316–12.407]	0.370 [0.271–0.504]	26.317 [14.200–48.773]	0.9128
*VIM*	8	47 [43–51]	88 [86–90]	5.236 [3.915–7.003]	0.509 [0.389–0.665]	12.636 [7.216–22.128]	0.8695
*MGMT*	6	34 [30–40]	94 [90–97]	4.859 [1.914–12.331]	0.733 [0.633–0.850]	6.984 [2.890–16.877]	0.6091
*MLH1*	4	17 [12–23]	99 [96–100]	5.530 [0.502–60.878]	0.877 [0.754–1.021]	6.353 [0.504–80.138]	0.3237
*HLTF*	4	28 [19–37]	95 [90–97]	5.464 [2.857–10.447]	0.771 [0.677–0.879]	7.808 [3.592–16.973]	0.7726
*SFRP1*	4	68 [61–75]	92 [86–96]	7.786 [4.563–13.286]	0.349 [0.191–0.637]	30.237 [14.850–61.567]	0.9264
*APC*	3	24 [16–32]	100 [97–100]	15.551 [3.053–79.207]	0.782 [0.710–0.861]	20.137 [3.764–107.73]	0.6715
*P16*	4	27.5 [21–35]	96 [91–99]	5.331 [1.888–15.052]	0.761 [0.692–0.839]	7.330 [2.759–19.476]	0.4028
*NDRG4*	3	57 [50–64]	95 [90–93]	10.027 [4.585–21.929]	0.464 [0.269–0.802]	24.374 [10.115–58.730]	0.9061

CRC: colorectal cancer, AA: advanced- adenoma, NAA: non-advanced adenoma, HP: hyperplastic polyp, TA: total adenoma TP: total patients, PLR: positive likelihood ratio, NLR: negative likelihood ratio, DOR: diagnosis odd ratio, AUC: area under the ROC curve

### Publication bias

**Fig 3:**
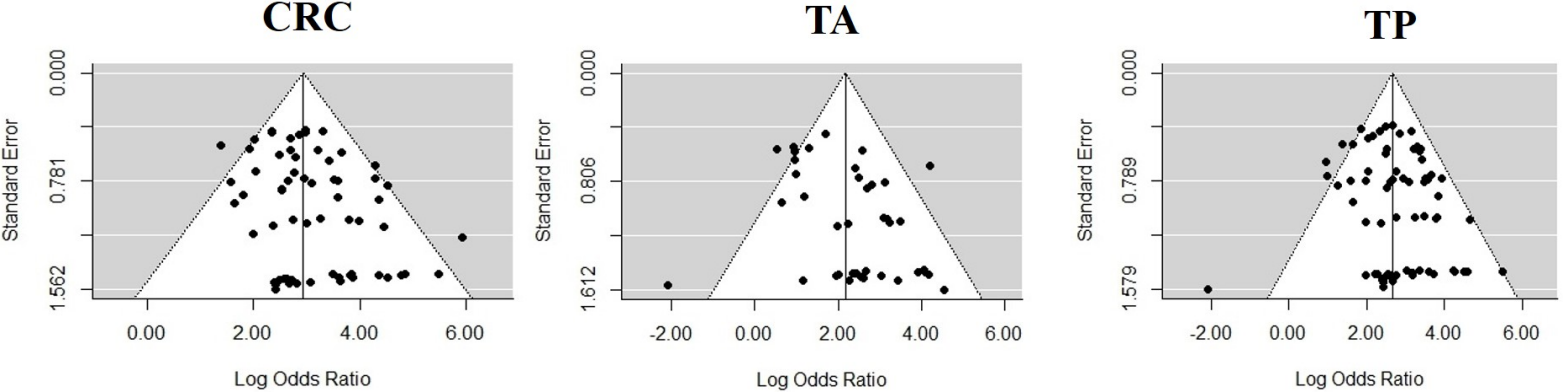
Publication bias of studies in different categories. CRC: colorectal cancer, TA: total adenoma, TP: total patients.

In our meta-analysis, publication bias was evaluated using Egger Test, a test for asymmetry of the funnel plot. The statistical results of Deek’s funnel plot did not show any obvious asymmetry and publication bias for all single-gene tests regardless of a specific gene in CRC (p-value = 0.904), TA (p-value = 0.486) and TP (p-value = 0.376) (Fig 3).

### Quality assessment

Quality assessment of the different studies found the greatest potential risk of bias, which came from patient selection, as most of the studies did not collect a consecutive or random sample (S2 Table and S3 File).

## Discussion

In the present study, we evaluated the clinical value of DNA hypermethylation of different genes as biomarkers for the diagnosis of CRC. Based on the pooled FP, FN, TP and TN of different methylated genes and considering the pooled sensitivity, specificity, PLR, NLR, DOR and SROC in different categories of CRC, the most diagnostic candidate genes were identified.

It is worth noting that a substantial heterogeneity and imprecision existed among the included studies was not due to non-standard or false identification/diagnosis of CRC and gene methylation. This was also reflected numerically as Q test and the I^2^ statistic which showed that the majority of analyses were not subjected to high heterogeneity (S4 File). While heterogeneity was inherent to any type of meta-analysis, the numerous analyses performed in the current study could reflect an authentic association.

The most advantage of this meta-analysis is the accuracy assessment of hypermethylated genes, which was calculated for each gene separately in the process of CRC (hyperplastic polyp, non-advanced adenoma, advanced adenoma, cancer tumor).

**Fig 4:**
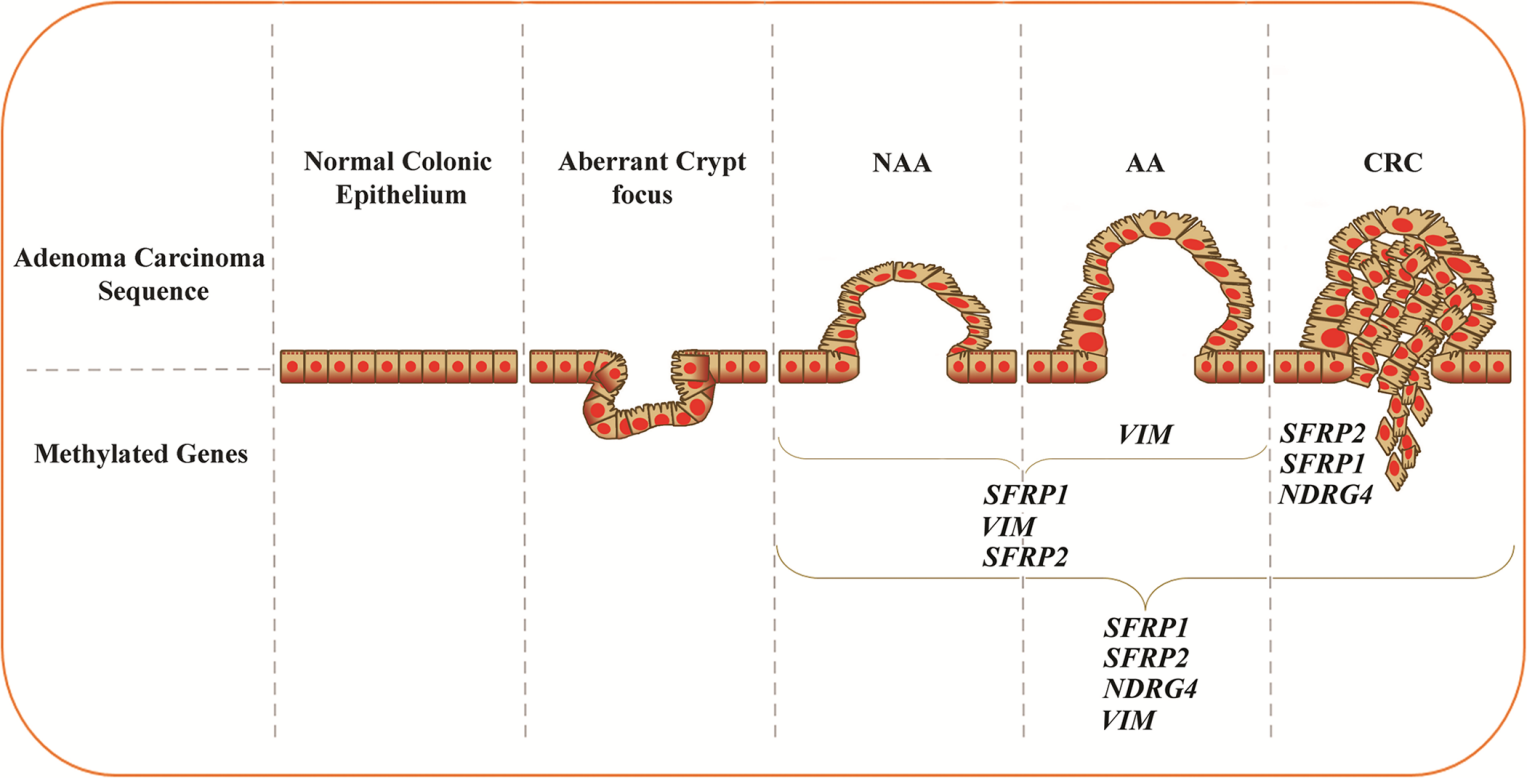
Methylation of candidate genes in different categories. Different genes are hypermethylated in development of CRC. CRC: colorectal cancer, AA: advanced-adenoma, NAA: non-advanced adenoma.

Based on our results, we concluded a methylation cascade of candidate genes in the process of colorectal cancer. The results demonstrated that *SFRP2*, *SFRP1* and *NDRG4* in CRC; *VIM* in AA; *SFRP1*, *VIM* and *SFRP2* in TA; *SFRP2* in HP; and *SFRP1*, *SFRP2*, *NDRG4* and *VIM* in TP patients offer the most accurate detection in their corresponding categories (Fig 4).

Zhang and his colleagues in their meta-analysis showed that *SFRP2* methylation serves as a promising marker with a great potential in early colorectal cancer diagnosis [52]. Fig 4 displays the methylated candidate genes in developing CRC. *SFRP1/ SFRP2* genes encode a member of the SFRP family that encodes soluble modulators of Wnt signaling. Epigenetic silencing of SFRP genes leads to downregulated activation of the Wnt-pathway which is often silenced by promoter hypermethylation in CRC [6, 53, 54]. *NDRG4* is a member of the NDRG family that includes a group of genes that have mostly tumor-suppressive effects. This novel candidate tumor suppressor gene, associated with energy balance and carcinogenesis, can inhibit PI3K/AKT signaling and controls cell growth and differentiation. *NDRG4* is downregulated by methylation in CRC [6, 53, 55]. The role of *NDRG4* as a tumor suppressor gene was demonstrated first by Melotte et al. They studied the *NDRG4* promoter methylation in the lines of colorectal cancerous cells, colorectal tissue and healthy colonic mucosa [34]. In August 2014, Cologuard^TM^ (Exact Sciences, Madison, WI, USA), a stool DNA test was approved by US FDA, based on molecular testing for the aberrant methylated regions of the promoters *NDRG4* and *BMP3*, muted *KRAS* and *β-actin* and an immunochemical test for human hemoglobin [4]. *VIM* is an intermediate filament protein and could increase the mechanical cell integrity and localize intracellular components. Aberrantly methylated *VIM* was already a diagnostic marker for early detection of CRC in the United States [6, 53, 56]. Hence, all candidate genes play important roles in the carcinogenesis of CRC.

Fecal immunochemical tests (FITs) and g-FOBTs are most commonly used in CRC screening programs. The sensitivity (specificity) of g-FOBT and FIT in cancer patients was 74.2% (95.7%) and 87.1% (91%), respectively, and in patients with AA, the sensitivity (specificity) of g-FOBT and FIT was 18.0% (97.4%) and 35.6% (97.2%), respectively [57]. Since the sensitivity is low for screening tests in both methods, researchers are seeking for other screening programs.

Methylated DNA is an attractive choice to serve as a biomarker substrate because CRCs harbor hundreds of aberrantly methylated genes [6]. Methylated DNA biomarkers can be detected in serum/plasma and stool of CRC patients [8, 58, 59]. Li et al. conducted a meta-analysis of DNA hypermethylation markers in peripheral blood for CRC detection. They found that single target gene had a sensitivity of 60% and a specificity of 94.3% for CRC detection [58]. A large number of studies verified the efficacy of detecting methylated DNA in stool to screen for early CRC and there are several meta-analyses assessing the diagnostic value of stool DNA testing [52, 60–62]. Zhai et al. reported the pooled sensitivities for single- and multiple-gene stool DNA (methylation and mutation) tests in CRC to be 48.0% and 77.8%, and the pooled specificity for single- and multiple-gene assays to be 97.0% and 92.7%, respectively [62]. Zhang and colleagues reported a sensitivity and specificity of combined single- and multiple-gene methylation analysis of stool DNA samples in CRC to be 73% and 92%, and for adenoma to be 51% and 92%, respectively [52]. In another study for methylated single- and multiple-gene tests in fecal samples, Luo et al. demonstrated an overall sensitivity of 62% and 54%, and a specificity of 89% and 88% in CRC and adenoma patients, respectively [60]. In the Qian et al., meta-analysis, the pooled sensitivity of the combined single- and multiple-gene DNA hypermethylation in stool was 71% and its specificity was 92% for CRC [61]. In the current study, single-gene stool DNA methylation analysis had a sensitivity (specificity) for CRC and adenoma of 56.5% (93.2%) and 32.6% (93.2%), respectively. These statistics demonstrated a lower sensitivity and specificity in compare to previous studies. Overall, there is a stool single-gene DNA methylation performance of 49.8% sensitivity and 92.9% specificity in diagnosis of CRC developing process in our study.

The difference in reported specificity and sensitivity among meta-analysis studies may reflect differences in included studies and their studied population. In our meta-analysis, fewer Chinese-based studies were included and single-gene stool DNA methylation tests were specifically considered. Individual study quality was assessed by QUADAS-2 tool to assess the quality of primary diagnostic accuracy studies. The quality assessment for all included studies revealed that there was a low risk of bias in all domains except in patient selection which was inevitable. As they were case-control studies they had similar criteria in their patient selection domain.

The current meta-analysis had several limitations which were considered when interpreting our results: (1) most publications included in the analysis were case-control studies and none of the included studies was a multicenter or randomized controlled trial, (2) in any meta-analysis, the effect of languages selection bias cannot be ignored, (3) studies on DNA methylation with statistical significance tend to be published and cited, (4) we excluded some of the valuable multi-gene studies from our meta-analysis due to the absence of sufficient data for each gene separately, and (5) the included studies did not account for the effect of sex, lifestyle, aging, diet and methodology on their findings.

In conclusion, our results demonstrated that *SFRP1* and *SFRP2* methylation assays, as non-invasive modalities, have promising accuracy for the detection of not only CRC but also the early stages of developing colorectal cancer. Besides, *NDRG4* and *VIM* could also be considered as significant diagnostic marker genes in CRC and adenoma, respectively.

Hence, this meta- analysis could be a helpful source for scientists to compare the diagnostic performance and accuracy of hypermethylated genes and could provide valuable insights into design for further proof-of-concept studies. Although in our meta-analysis each gene was calculated separately in the process of colorectal cancer, the results could be used for singular or combined, multi-marker assays.

## Supporting information

S1 File Performance of single-gene stool DNA methylation biomarker tests. (PDF)

S2 File Performance of a certain gene in single-gene stool-based DNA methylation biomarker tests. (PDF)

S3 File Graphical display of quality assessment results. (PDF)

S1 Table Characteristics of the 38 included studies. (XLSX)

S2 Table Quality of included studies. (PDF)

S3 Table PRISMA 2009 checklist. (PDF)

S4 File Threshold effect study using Spearman correlation coefficient. (PDF)

S4 Table Meta-analysis on genetic association studies form. (PDF)
