# Supplementary material for: The importance of stool DNA methylation in colorectal cancer diagnosis: A meta-analysis
Source: PLoS One. 2018 Jul 19;13(7):e0200735. doi: 10.1371/journal.pone.0200735 (PMC6053185; doi:10.1371/journal.pone.0200735)
Supplement: S1 File — (PDF) [file pone.0200735.s001.pdf]

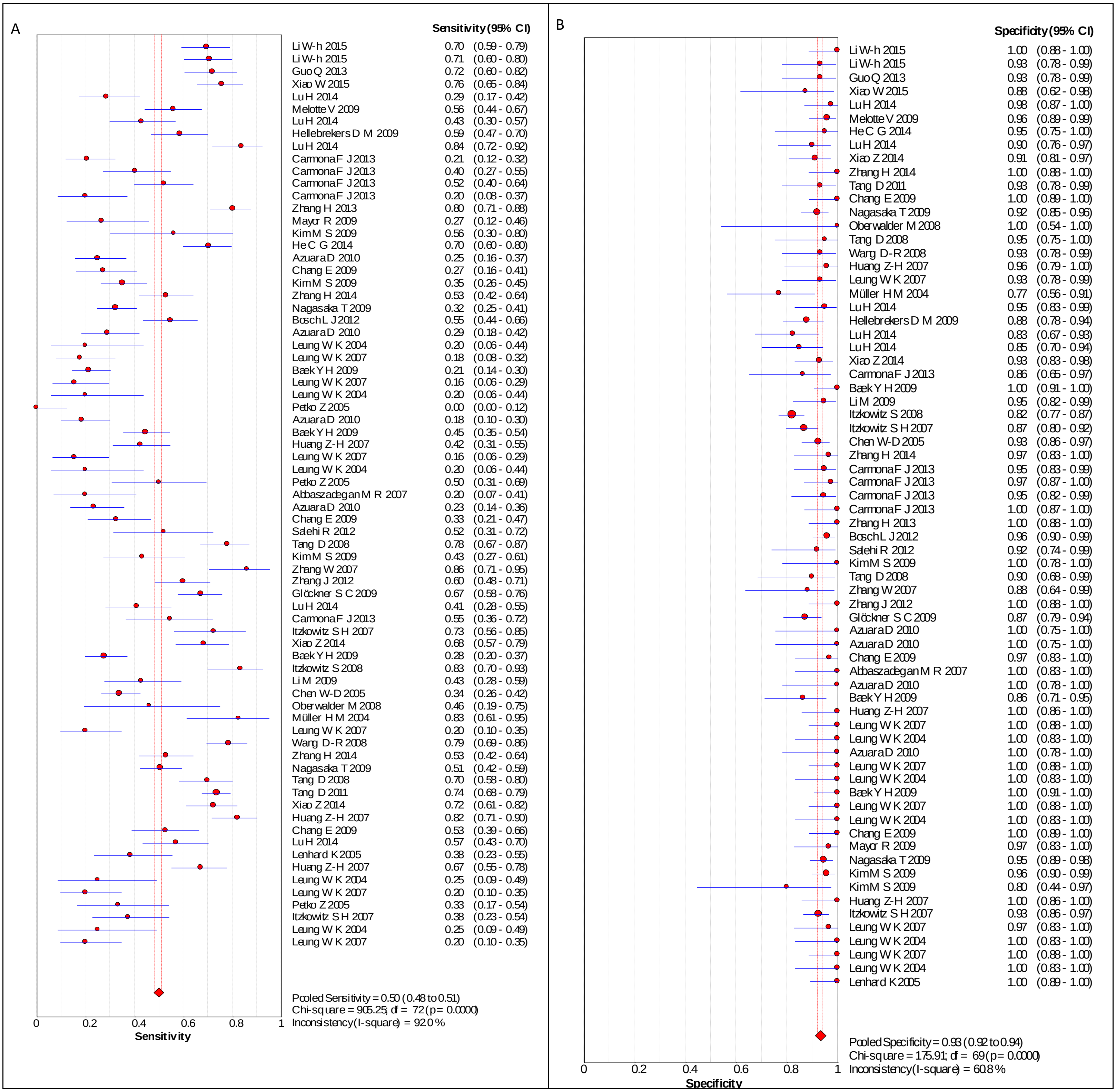
A
Sensitivity (95% CI)
Li W-h 2015 0.70 (0.59 - 0.79)
Li W-h 2015 0.71 (0.60 - 0.80)
Guo Q 2013 0.72 (0.60 - 0.82)
Xiao W 2015 0.76 (0.65 - 0.84)
Lu H 2014 0.29 (0.17 - 0.42)
Melotte V 2009 0.56 (0.44 - 0.67)
Lu H 2014 0.43 (0.30 - 0.57)
Hellebrekers D M 2009 0.59 (0.47 - 0.70)
Lu H 2014 0.84 (0.72 - 0.92)
Carmona F J 2013 0.21 (0.12 - 0.32)
Carmona F J 2013 0.40 (0.27 - 0.55)
Carmona F J 2013 0.52 (0.40 - 0.64)
Carmona F J 2013 0.20 (0.08 - 0.37)
Zhang H 2013 0.80 (0.71 - 0.88)
Mayor R 2009 0.27 (0.12 - 0.46)
Kim M S 2009 0.56 (0.30 - 0.80)
He C G 2014 0.70 (0.60 - 0.80)
Azuara D 2010 0.25 (0.16 - 0.37)
Chang E 2009 0.27 (0.16 - 0.41)
Kim M S 2009 0.35 (0.26 - 0.45)
Zhang H 2014 0.53 (0.42 - 0.64)
Nagasaka T 2009 0.32 (0.25 - 0.41)
Bosch L J 2012 0.55 (0.44 - 0.66)
Azuara D 2010 0.29 (0.18 - 0.42)
Leung W K 2004 0.20 (0.06 - 0.44)
Leung W K 2007 0.18 (0.08 - 0.32)
Baek Y H 2009 0.21 (0.14 - 0.30)
Leung W K 2007 0.16 (0.06 - 0.29)
Leung W K 2004 0.20 (0.06 - 0.44)
Petko Z 2005 0.00 (0.00 - 0.12)
Azuara D 2010 0.18 (0.10 - 0.30)
Baek Y H 2009 0.45 (0.35 - 0.54)
Huang Z-H 2007 0.42 (0.31 - 0.55)
Leung W K 2007 0.16 (0.06 - 0.29)
Leung W K 2004 0.20 (0.06 - 0.44)
Petko Z 2005 0.50 (0.31 - 0.69)
Abbaszadegan M R 2007 0.20 (0.07 - 0.41)
Azuara D 2010 0.23 (0.14 - 0.36)
Chang E 2009 0.33 (0.21 - 0.47)
Salehi R 2012 0.52 (0.31 - 0.72)
Tang D 2008 0.78 (0.67 - 0.87)
Kim M S 2009 0.43 (0.27 - 0.61)
Zhang W 2007 0.86 (0.71 - 0.95)
Zhang J 2012 0.60 (0.48 - 0.71)
Glöckner S C 2009 0.67 (0.58 - 0.76)
Lu H 2014 0.41 (0.28 - 0.55)
Carmona F J 2013 0.55 (0.36 - 0.72)
Itzkowitz S H 2007 0.73 (0.56 - 0.85)
Xiao Z 2014 0.68 (0.57 - 0.79)
Baek Y H 2009 0.28 (0.20 - 0.37)
Itzkowitz S 2008 0.83 (0.70 - 0.93)
Li M 2009 0.43 (0.28 - 0.59)
Chen W-D 2005 0.34 (0.26 - 0.42)
Oberwalder M 2008 0.46 (0.19 - 0.75)
Müller H M 2004 0.83 (0.61 - 0.95)
Leung W K 2007 0.20 (0.10 - 0.35)
Wang D-R 2008 0.79 (0.69 - 0.86)
Zhang H 2014 0.53 (0.42 - 0.64)
Nagasaka T 2009 0.51 (0.42 - 0.59)
Tang D 2008 0.70 (0.58 - 0.80)
Tang D 2011 0.74 (0.68 - 0.79)
Xiao Z 2014 0.72 (0.61 - 0.82)
Huang Z-H 2007 0.82 (0.71 - 0.90)
Chang E 2009 0.53 (0.39 - 0.66)
Lu H 2014 0.57 (0.43 - 0.70)
Lenhard K 2005 0.38 (0.23 - 0.55)
Huang Z-H 2007 0.67 (0.55 - 0.78)
Leung W K 2004 0.25 (0.09 - 0.49)
Leung W K 2007 0.20 (0.10 - 0.35)
Petko Z 2005 0.33 (0.17 - 0.54)
Itzkowitz S H 2007 0.38 (0.23 - 0.54)
Leung W K 2004 0.25 (0.09 - 0.49)
Leung W K 2007 0.20 (0.10 - 0.35)
Pooled Sensitivity = 0.50 (0.48 to 0.51)
Chi-square = 905.25; df = 72 (p = 0.0000)
Inconsistency (I-square) = 92.0 %
0 0.2 0.4 0.6 0.8 1
Sensitivity
B
Specificity (95% CI)
Li W-h 2015 1.00 (0.88 - 1.00)
Li W-h 2015 0.93 (0.78 - 0.99)
Guo Q 2013 0.93 (0.78 - 0.99)
Xiao W 2015 0.88 (0.62 - 0.98)
Lu H 2014 0.98 (0.87 - 1.00)
Melotte V 2009 0.96 (0.89 - 0.99)
He C G 2014 0.95 (0.75 - 1.00)
Lu H 2014 0.90 (0.76 - 0.97)
Xiao Z 2014 0.91 (0.81 - 0.97)
Zhang H 2014 1.00 (0.88 - 1.00)
Tang D 2011 0.93 (0.78 - 0.99)
Chang E 2009 1.00 (0.89 - 1.00)
Nagasaka T 2009 0.92 (0.85 - 0.96)
Oberwalder M 2008 1.00 (0.54 - 1.00)
Tang D 2008 0.95 (0.75 - 1.00)
Wang D-R 2008 0.93 (0.78 - 0.99)
Huang Z-H 2007 0.96 (0.79 - 1.00)
Leung W K 2007 0.93 (0.78 - 0.99)
Müller H M 2004 0.77 (0.56 - 0.91)
Lu H 2014 0.95 (0.83 - 0.99)
Hellebrekers D M 2009 0.88 (0.78 - 0.94)
Lu H 2014 0.83 (0.67 - 0.93)
Lu H 2014 0.85 (0.70 - 0.94)
Xiao Z 2014 0.93 (0.83 - 0.98)
Carmona F J 2013 0.86 (0.65 - 0.97)
Baek Y H 2009 1.00 (0.91 - 1.00)
Li M 2009 0.95 (0.82 - 0.99)
Itzkowitz S 2008 0.82 (0.77 - 0.87)
Itzkowitz S H 2007 0.87 (0.80 - 0.92)
Chen W-D 2005 0.93 (0.86 - 0.97)
Zhang H 2014 0.97 (0.83 - 1.00)
Carmona F J 2013 0.95 (0.83 - 0.99)
Carmona F J 2013 0.97 (0.87 - 1.00)
Carmona F J 2013 0.95 (0.82 - 0.99)
Carmona F J 2013 1.00 (0.87 - 1.00)
Zhang H 2013 1.00 (0.88 - 1.00)
Bosch L J 2012 0.96 (0.90 - 0.99)
Salehi R 2012 0.92 (0.74 - 0.99)
Kim M S 2009 1.00 (0.78 - 1.00)
Tang D 2008 0.90 (0.68 - 0.99)
Zhang W 2007 0.88 (0.64 - 0.99)
Zhang J 2012 1.00 (0.88 - 1.00)
Glöckner S C 2009 0.87 (0.79 - 0.94)
Azuara D 2010 1.00 (0.75 - 1.00)
Azuara D 2010 1.00 (0.75 - 1.00)
Chang E 2009 0.97 (0.83 - 1.00)
Abbaszadegan M R 2007 1.00 (0.83 - 1.00)
Azuara D 2010 1.00 (0.78 - 1.00)
Baek Y H 2009 0.86 (0.71 - 0.95)
Huang Z-H 2007 1.00 (0.86 - 1.00)
Leung W K 2007 1.00 (0.88 - 1.00)
Leung W K 2004 1.00 (0.83 - 1.00)
Azuara D 2010 1.00 (0.78 - 1.00)
Leung W K 2007 1.00 (0.88 - 1.00)
Leung W K 2004 1.00 (0.83 - 1.00)
Baek Y H 2009 1.00 (0.91 - 1.00)
Leung W K 2007 1.00 (0.88 - 1.00)
Leung W K 2004 1.00 (0.83 - 1.00)
Chang E 2009 1.00 (0.89 - 1.00)
Mayor R 2009 0.97 (0.83 - 1.00)
Nagasaka T 2009 0.95 (0.89 - 0.98)
Kim M S 2009 0.96 (0.90 - 0.99)
Kim M S 2009 0.80 (0.44 - 0.97)
Huang Z-H 2007 1.00 (0.86 - 1.00)
Itzkowitz S H 2007 0.93 (0.86 - 0.97)
Leung W K 2007 0.97 (0.83 - 1.00)
Leung W K 2004 1.00 (0.83 - 1.00)
Leung W K 2007 1.00 (0.88 - 1.00)
Leung W K 2004 1.00 (0.83 - 1.00)
Lenhard K 2005 1.00 (0.89 - 1.00)
Pooled Specificity = 0.93 (0.92 to 0.94)
Chi-square = 175.91; df = 69 (p = 0.0000)
Inconsistency (I-square) = 60.8 %
0 0.2 0.4 0.6 0.8 1
Specificity

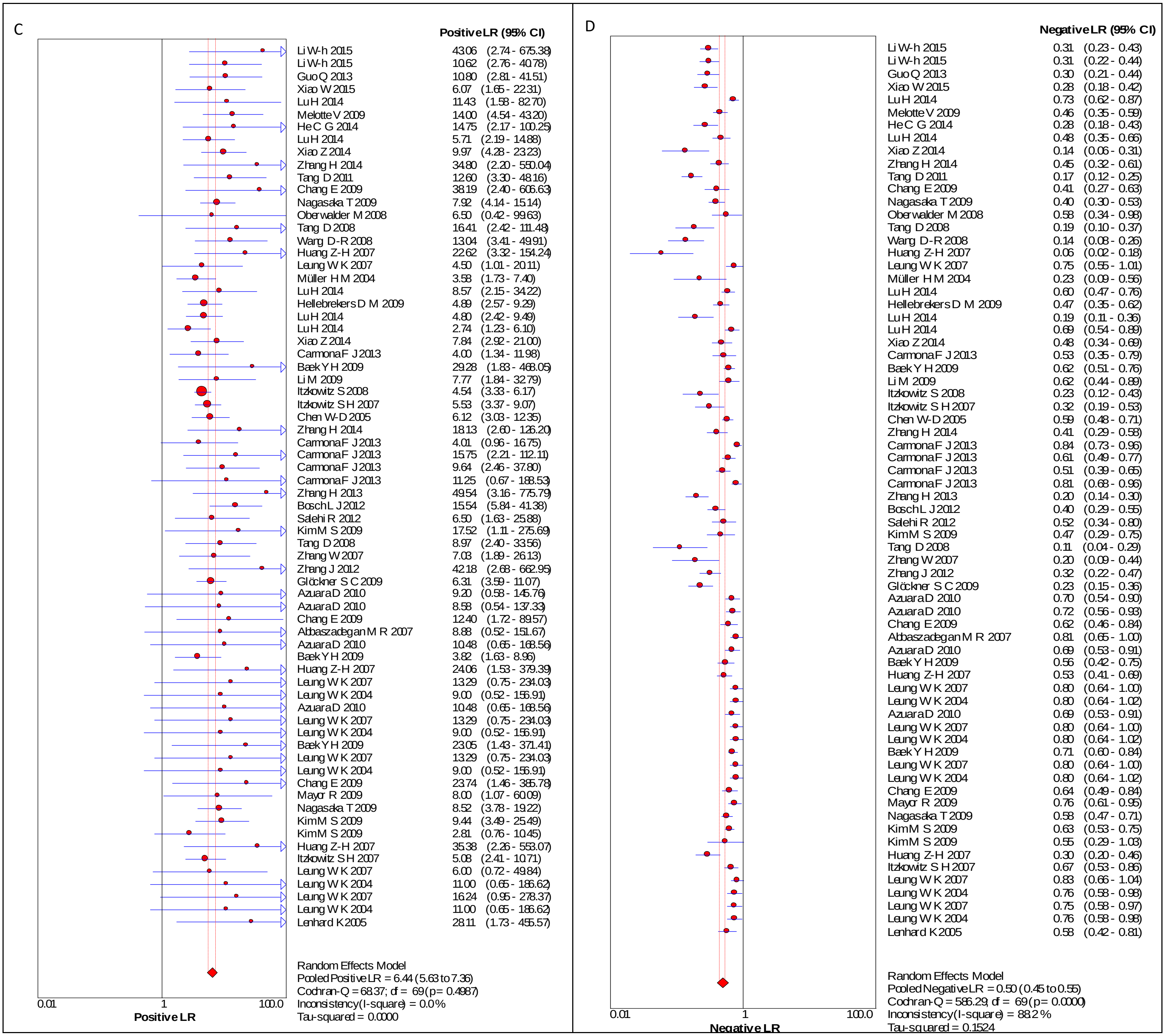

C
Positive LR (95% CI)
Li W-h 2015 43.06 (2.74 - 675.38)
Li W-h 2015 10.62 (2.76 - 40.78)
Guo Q 2013 10.80 (2.81 - 41.51)
Xiao W 2015 6.07 (1.65 - 22.31)
Lu H 2014 11.43 (1.58 - 82.70)
Melotte V 2009 14.00 (4.54 - 43.20)
He C G 2014 14.75 (2.17 - 100.25)
Lu H 2014 5.71 (2.19 - 14.88)
Xiao Z 2014 9.97 (4.28 - 23.23)
Zhang H 2014 34.80 (2.20 - 550.04)
Tang D 2011 12.60 (3.30 - 48.16)
Chang E 2009 38.19 (2.40 - 606.63)
Nagasaka T 2009 7.92 (4.14 - 15.14)
Oberwalder M 2008 6.50 (0.42 - 99.63)
Tang D 2008 16.41 (2.42 - 111.48)
Wang D-R 2008 13.04 (3.41 - 49.91)
Huang Z-H 2007 22.62 (3.32 - 154.24)
Leung W K 2007 4.50 (1.01 - 20.11)
Müller H M 2004 3.58 (1.73 - 7.40)
Lu H 2014 8.57 (2.15 - 34.22)
Hellebrekers D M 2009 4.89 (2.57 - 9.29)
Lu H 2014 4.80 (2.42 - 9.49)
Lu H 2014 2.74 (1.23 - 6.10)
Xiao Z 2014 7.84 (2.92 - 21.00)
Carmona F J 2013 4.00 (1.34 - 11.98)
Baek Y H 2009 29.28 (1.83 - 468.05)
Li M 2009 7.77 (1.84 - 32.79)
Itzkowitz S 2008 4.54 (3.33 - 6.17)
Itzkowitz S H 2007 5.53 (3.37 - 9.07)
Chen W-D 2005 6.12 (3.03 - 12.35)
Zhang H 2014 18.13 (2.60 - 126.20)
Carmona F J 2013 4.01 (0.96 - 16.75)
Carmona F J 2013 15.75 (2.21 - 112.11)
Carmona F J 2013 9.64 (2.46 - 37.80)
Carmona F J 2013 11.25 (0.67 - 188.53)
Zhang H 2013 49.54 (3.16 - 775.79)
Bosch L J 2012 15.54 (5.84 - 41.38)
Salehi R 2012 6.50 (1.63 - 25.88)
Kim M S 2009 17.52 (1.11 - 275.69)
Tang D 2008 8.97 (2.40 - 33.56)
Zhang W 2007 7.03 (1.89 - 26.13)
Zhang J 2012 42.18 (2.68 - 662.95)
Glöckner S C 2009 6.31 (3.59 - 11.07)
Azuara D 2010 9.20 (0.58 - 145.76)
Azuara D 2010 8.58 (0.54 - 137.33)
Chang E 2009 12.40 (1.72 - 89.57)
Abbaszadegan M R 2007 8.88 (0.52 - 151.67)
Azuara D 2010 10.48 (0.65 - 168.56)
Baek Y H 2009 3.82 (1.63 - 8.96)
Huang Z-H 2007 24.06 (1.53 - 379.39)
Leung W K 2007 13.29 (0.75 - 234.03)
Leung W K 2004 9.00 (0.52 - 156.91)
Azuara D 2010 10.48 (0.65 - 168.56)
Leung W K 2007 13.29 (0.75 - 234.03)
Leung W K 2004 9.00 (0.52 - 156.91)
Baek Y H 2009 23.05 (1.43 - 371.41)
Leung W K 2007 13.29 (0.75 - 234.03)
Leung W K 2004 9.00 (0.52 - 156.91)
Chang E 2009 23.74 (1.46 - 385.78)
Mayor R 2009 8.00 (1.07 - 60.09)
Nagasaka T 2009 8.52 (3.78 - 19.22)
Kim M S 2009 9.44 (3.49 - 25.49)
Kim M S 2009 2.81 (0.76 - 10.45)
Huang Z-H 2007 35.38 (2.26 - 553.07)
Itzkowitz S H 2007 5.08 (2.41 - 10.71)
Leung W K 2007 6.00 (0.72 - 49.84)
Leung W K 2004 11.00 (0.65 - 186.62)
Leung W K 2007 16.24 (0.95 - 278.37)
Leung W K 2004 11.00 (0.65 - 186.62)
Lenhard K 2005 28.11 (1.73 - 455.57)
Random Effects Model
Pooled Positive LR = 6.44 (5.63 to 7.36)
Cochran-Q = 68.37; df = 69 (p = 0.4987)
Inconsistency (I-square) = 0.0 %
Tau-squared = 0.0000
0.01
1
100.0
Positive LR
D
Negative LR (95% CI)
Li W-h 2015 0.31 (0.23 - 0.43)
Li W-h 2015 0.31 (0.22 - 0.44)
Guo Q 2013 0.30 (0.21 - 0.44)
Xiao W 2015 0.28 (0.18 - 0.42)
Lu H 2014 0.73 (0.62 - 0.87)
Melotte V 2009 0.46 (0.35 - 0.59)
He C G 2014 0.28 (0.18 - 0.43)
Lu H 2014 0.48 (0.35 - 0.66)
Xiao Z 2014 0.14 (0.06 - 0.31)
Zhang H 2014 0.45 (0.32 - 0.61)
Tang D 2011 0.17 (0.12 - 0.25)
Chang E 2009 0.41 (0.27 - 0.63)
Nagasaka T 2009 0.40 (0.30 - 0.53)
Oberwalder M 2008 0.58 (0.34 - 0.98)
Tang D 2008 0.19 (0.10 - 0.37)
Wang D-R 2008 0.14 (0.08 - 0.26)
Huang Z-H 2007 0.06 (0.02 - 0.18)
Leung W K 2007 0.75 (0.55 - 1.01)
Müller H M 2004 0.23 (0.09 - 0.56)
Lu H 2014 0.60 (0.47 - 0.76)
Hellebrekers D M 2009 0.47 (0.35 - 0.62)
Lu H 2014 0.19 (0.11 - 0.36)
Lu H 2014 0.69 (0.54 - 0.89)
Xiao Z 2014 0.48 (0.34 - 0.69)
Carmona F J 2013 0.53 (0.35 - 0.79)
Baek Y H 2009 0.62 (0.51 - 0.76)
Li M 2009 0.62 (0.44 - 0.89)
Itzkowitz S 2008 0.23 (0.12 - 0.43)
Itzkowitz S H 2007 0.32 (0.19 - 0.53)
Chen W-D 2005 0.59 (0.48 - 0.71)
Zhang H 2014 0.41 (0.29 - 0.58)
Carmona F J 2013 0.84 (0.73 - 0.96)
Carmona F J 2013 0.61 (0.49 - 0.77)
Carmona F J 2013 0.51 (0.39 - 0.65)
Carmona F J 2013 0.81 (0.68 - 0.96)
Zhang H 2013 0.20 (0.14 - 0.30)
Bosch L J 2012 0.40 (0.29 - 0.55)
Salehi R 2012 0.52 (0.34 - 0.80)
Kim M S 2009 0.47 (0.29 - 0.75)
Tang D 2008 0.11 (0.04 - 0.29)
Zhang W 2007 0.20 (0.09 - 0.44)
Zhang J 2012 0.32 (0.22 - 0.47)
Glöckner S C 2009 0.23 (0.15 - 0.36)
Azuara D 2010 0.70 (0.54 - 0.90)
Azuara D 2010 0.72 (0.56 - 0.93)
Chang E 2009 0.62 (0.46 - 0.84)
Abbaszadegan M R 2007 0.81 (0.65 - 1.00)
Azuara D 2010 0.69 (0.53 - 0.91)
Baek Y H 2009 0.56 (0.42 - 0.75)
Huang Z-H 2007 0.53 (0.41 - 0.69)
Leung W K 2007 0.80 (0.64 - 1.00)
Leung W K 2004 0.80 (0.64 - 1.02)
Azuara D 2010 0.69 (0.53 - 0.91)
Leung W K 2007 0.80 (0.64 - 1.00)
Leung W K 2004 0.80 (0.64 - 1.02)
Baek Y H 2009 0.71 (0.60 - 0.84)
Leung W K 2007 0.80 (0.64 - 1.00)
Leung W K 2004 0.80 (0.64 - 1.02)
Chang E 2009 0.64 (0.49 - 0.84)
Mayor R 2009 0.76 (0.61 - 0.95)
Nagasaka T 2009 0.58 (0.47 - 0.71)
Kim M S 2009 0.63 (0.53 - 0.75)
Kim M S 2009 0.55 (0.29 - 1.03)
Huang Z-H 2007 0.30 (0.20 - 0.46)
Itzkowitz S H 2007 0.67 (0.53 - 0.86)
Leung W K 2007 0.83 (0.66 - 1.04)
Leung W K 2004 0.76 (0.58 - 0.98)
Leung W K 2007 0.75 (0.58 - 0.97)
Leung W K 2004 0.76 (0.58 - 0.98)
Lenhard K 2005 0.58 (0.42 - 0.81)
Random Effects Model
Pooled Negative LR = 0.50 (0.45 to 0.55)
Cochran-Q = 586.29; df = 69 (p = 0.0000)
Inconsistency (I-square) = 88.2 %
Tau-squared = 0.1524
0.01
1
100.0
Negative LR

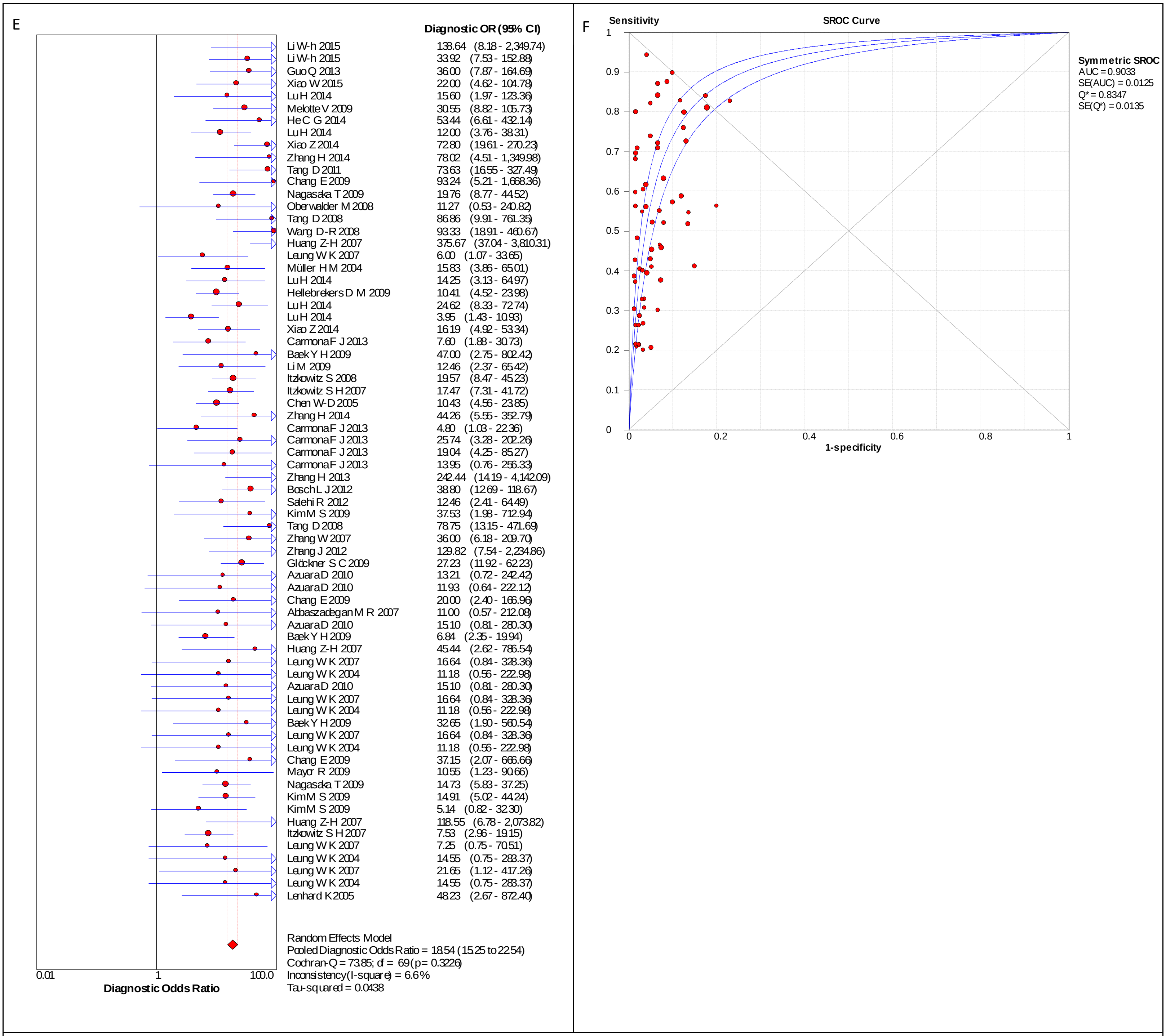

Figure S1.1. The summary performance of single-gene stool-based DNA methylation biomarker tests in all studies in CRC (A) sensitivity, (B) specificity, (C) positive likelihood ratios, (D) negative likelihood ratios, (E) diagnostic odds ratio, (F) summary ROC curves.

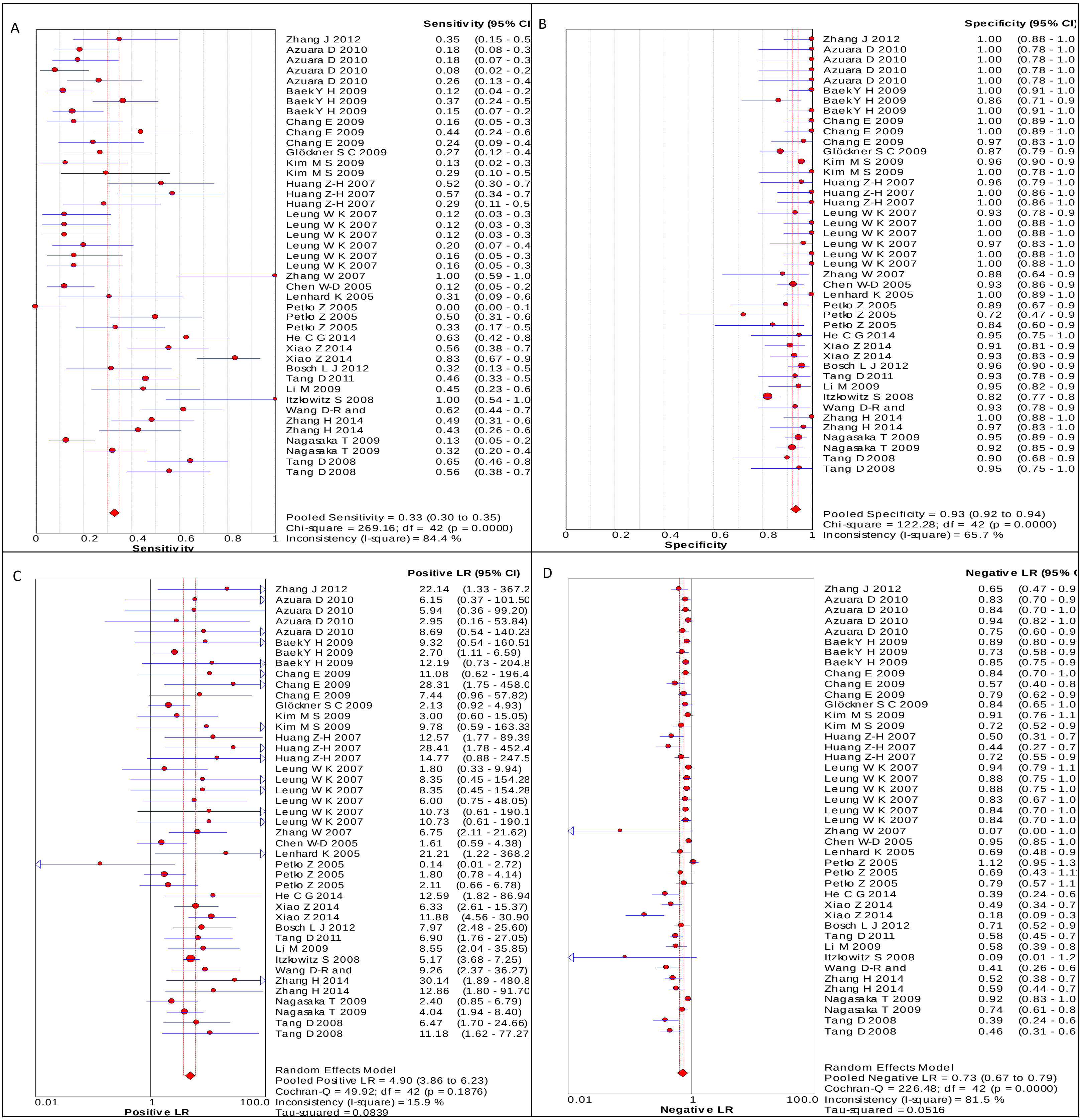
A
Sensitivity (95% CI
Zhang J 2012 0.35 (0.15 - 0.5
Azuara D 2010 0.18 (0.08 - 0.3
Azuara D 2010 0.18 (0.07 - 0.3
Azuara D 2010 0.08 (0.02 - 0.2
Azuara D 2010 0.26 (0.13 - 0.4
BaekY H 2009 0.12 (0.04 - 0.2
BaekY H 2009 0.37 (0.24 - 0.5
BaekY H 2009 0.15 (0.07 - 0.2
Chang E 2009 0.16 (0.05 - 0.3
Chang E 2009 0.44 (0.24 - 0.6
Chang E 2009 0.24 (0.09 - 0.4
Glöckner S C 2009 0.27 (0.12 - 0.4
Kim M S 2009 0.13 (0.02 - 0.3
Kim M S 2009 0.29 (0.10 - 0.5
Huang Z-H 2007 0.52 (0.30 - 0.7
Huang Z-H 2007 0.57 (0.34 - 0.7
Huang Z-H 2007 0.29 (0.11 - 0.5
Leung W K 2007 0.12 (0.03 - 0.3
Leung W K 2007 0.12 (0.03 - 0.3
Leung W K 2007 0.12 (0.03 - 0.3
Leung W K 2007 0.20 (0.07 - 0.4
Leung W K 2007 0.16 (0.05 - 0.3
Leung W K 2007 0.16 (0.05 - 0.3
Zhang W 2007 1.00 (0.59 - 1.0
Chen W-D 2005 0.12 (0.05 - 0.2
Lenhard K 2005 0.31 (0.09 - 0.6
Petko Z 2005 0.00 (0.00 - 0.1
Petko Z 2005 0.50 (0.31 - 0.6
Petko Z 2005 0.33 (0.17 - 0.5
He C G 2014 0.63 (0.42 - 0.8
Xiao Z 2014 0.56 (0.38 - 0.7
Xiao Z 2014 0.83 (0.67 - 0.9
Bosch L J 2012 0.32 (0.13 - 0.5
Tang D 2011 0.46 (0.33 - 0.5
Li M 2009 0.45 (0.23 - 0.6
Itzkowitz S 2008 1.00 (0.54 - 1.0
Wang D-R and 0.62 (0.44 - 0.7
Zhang H 2014 0.49 (0.31 - 0.6
Zhang H 2014 0.43 (0.26 - 0.6
Nagasaka T 2009 0.13 (0.05 - 0.2
Nagasaka T 2009 0.32 (0.20 - 0.4
Tang D 2008 0.65 (0.46 - 0.8
Tang D 2008 0.56 (0.38 - 0.7
Pooled Sensitivity = 0.33 (0.30 to 0.35)
Chi-square = 269.16; df = 42 (p = 0.0000)
Inconsistency (I-square) = 84.4 %
0 0.2 0.4 0.6 0.8 1
Sensitivity
B
Specificity (95% CI)
Zhang J 2012 1.00 (0.88 - 1.0
Azuara D 2010 1.00 (0.78 - 1.0
Azuara D 2010 1.00 (0.78 - 1.0
Azuara D 2010 1.00 (0.78 - 1.0
Azuara D 2010 1.00 (0.78 - 1.0
BaekY H 2009 1.00 (0.91 - 1.0
BaekY H 2009 0.86 (0.71 - 0.9
BaekY H 2009 1.00 (0.91 - 1.0
Chang E 2009 1.00 (0.89 - 1.0
Chang E 2009 1.00 (0.89 - 1.0
Chang E 2009 0.97 (0.83 - 1.0
Glöckner S C 2009 0.87 (0.79 - 0.9
Kim M S 2009 0.96 (0.90 - 0.9
Kim M S 2009 1.00 (0.78 - 1.0
Huang Z-H 2007 0.96 (0.79 - 1.0
Huang Z-H 2007 1.00 (0.86 - 1.0
Huang Z-H 2007 1.00 (0.86 - 1.0
Leung W K 2007 0.93 (0.78 - 0.9
Leung W K 2007 1.00 (0.88 - 1.0
Leung W K 2007 1.00 (0.88 - 1.0
Leung W K 2007 0.97 (0.83 - 1.0
Leung W K 2007 1.00 (0.88 - 1.0
Leung W K 2007 1.00 (0.88 - 1.0
Zhang W 2007 0.88 (0.64 - 0.9
Chen W-D 2005 0.93 (0.86 - 0.9
Lenhard K 2005 1.00 (0.89 - 1.0
Petko Z 2005 0.89 (0.67 - 0.9
Petko Z 2005 0.72 (0.47 - 0.9
Petko Z 2005 0.84 (0.60 - 0.9
He C G 2014 0.95 (0.75 - 1.0
Xiao Z 2014 0.91 (0.81 - 0.9
Xiao Z 2014 0.93 (0.83 - 0.9
Bosch L J 2012 0.96 (0.90 - 0.9
Tang D 2011 0.93 (0.78 - 0.9
Li M 2009 0.95 (0.82 - 0.9
Itzkowitz S 2008 0.82 (0.77 - 0.8
Wang D-R and 0.93 (0.78 - 0.9
Zhang H 2014 1.00 (0.88 - 1.0
Zhang H 2014 0.97 (0.83 - 1.0
Nagasaka T 2009 0.95 (0.89 - 0.9
Nagasaka T 2009 0.92 (0.85 - 0.9
Tang D 2008 0.90 (0.68 - 0.9
Tang D 2008 0.95 (0.75 - 1.0
Pooled Specificity = 0.93 (0.92 to 0.94)
Chi-square = 122.28; df = 42 (p = 0.0000)
Inconsistency (I-square) = 65.7 %
0 0.2 0.4 0.6 0.8 1
Specificity
C
Positive LR (95% CI)
Zhang J 2012 22.14 (1.33 - 367.2
Azuara D 2010 6.15 (0.37 - 101.50
Azuara D 2010 5.94 (0.36 - 99.20)
Azuara D 2010 2.95 (0.16 - 53.84)
Azuara D 2010 8.69 (0.54 - 140.23
BaekY H 2009 9.32 (0.54 - 160.51
BaekY H 2009 2.70 (1.11 - 6.59)
BaekY H 2009 12.19 (0.73 - 204.8
Chang E 2009 11.08 (0.62 - 196.4
Chang E 2009 28.31 (1.75 - 458.0
Chang E 2009 7.44 (0.96 - 57.82)
Glöckner S C 2009 2.13 (0.92 - 4.93)
Kim M S 2009 3.00 (0.60 - 15.05)
Kim M S 2009 9.78 (0.59 - 163.33
Huang Z-H 2007 12.57 (1.77 - 89.39
Huang Z-H 2007 28.41 (1.78 - 452.4
Huang Z-H 2007 14.77 (0.88 - 247.5
Leung W K 2007 1.80 (0.33 - 9.94)
Leung W K 2007 8.35 (0.45 - 154.28
Leung W K 2007 8.35 (0.45 - 154.28
Leung W K 2007 6.00 (0.75 - 48.05)
Leung W K 2007 10.73 (0.61 - 190.1
Leung W K 2007 10.73 (0.61 - 190.1
Zhang W 2007 6.75 (2.11 - 21.62)
Chen W-D 2005 1.61 (0.59 - 4.38)
Lenhard K 2005 21.21 (1.22 - 368.2
Petko Z 2005 0.14 (0.01 - 2.72)
Petko Z 2005 1.80 (0.78 - 4.14)
Petko Z 2005 2.11 (0.66 - 6.78)
He C G 2014 12.59 (1.82 - 86.94
Xiao Z 2014 6.33 (2.61 - 15.37)
Xiao Z 2014 11.88 (4.56 - 30.90
Bosch L J 2012 7.97 (2.48 - 25.60)
Tang D 2011 6.90 (1.76 - 27.05)
Li M 2009 8.55 (2.04 - 35.85)
Itzkowitz S 2008 5.17 (3.68 - 7.25)
Wang D-R and 9.26 (2.37 - 36.27)
Zhang H 2014 30.14 (1.89 - 480.8
Zhang H 2014 12.86 (1.80 - 91.70
Nagasaka T 2009 2.40 (0.85 - 6.79)
Nagasaka T 2009 4.04 (1.94 - 8.40)
Tang D 2008 6.47 (1.70 - 24.66)
Tang D 2008 11.18 (1.62 - 77.27
Random Effects Model
Pooled Positive LR = 4.90 (3.86 to 6.23)
Cochran-Q = 49.92; df = 42 (p = 0.1876)
Inconsistency (I-square) = 15.9 %
Tau-squared = 0.0839
0.01 1 100.0
Positive LR
D
Negative LR (95% (
Zhang J 2012 0.65 (0.47 - 0.9
Azuara D 2010 0.83 (0.70 - 0.9
Azuara D 2010 0.84 (0.70 - 1.0
Azuara D 2010 0.94 (0.82 - 1.0
Azuara D 2010 0.75 (0.60 - 0.9
BaekY H 2009 0.89 (0.80 - 0.9
BaekY H 2009 0.73 (0.58 - 0.9
BaekY H 2009 0.85 (0.75 - 0.9
Chang E 2009 0.84 (0.70 - 1.0
Chang E 2009 0.57 (0.40 - 0.8
Chang E 2009 0.79 (0.62 - 0.9
Glöckner S C 2009 0.84 (0.65 - 1.0
Kim M S 2009 0.91 (0.76 - 1.1
Kim M S 2009 0.72 (0.52 - 0.9
Huang Z-H 2007 0.50 (0.31 - 0.7
Huang Z-H 2007 0.44 (0.27 - 0.7
Huang Z-H 2007 0.72 (0.55 - 0.9
Leung W K 2007 0.94 (0.79 - 1.1
Leung W K 2007 0.88 (0.75 - 1.0
Leung W K 2007 0.88 (0.75 - 1.0
Leung W K 2007 0.83 (0.67 - 1.0
Leung W K 2007 0.84 (0.70 - 1.0
Leung W K 2007 0.84 (0.70 - 1.0
Zhang W 2007 0.07 (0.00 - 1.0
Chen W-D 2005 0.95 (0.85 - 1.0
Lenhard K 2005 0.69 (0.48 - 0.9
Petko Z 2005 1.12 (0.95 - 1.3
Petko Z 2005 0.69 (0.43 - 1.1
Petko Z 2005 0.79 (0.57 - 1.1
He C G 2014 0.39 (0.24 - 0.6
Xiao Z 2014 0.49 (0.34 - 0.7
Xiao Z 2014 0.18 (0.09 - 0.3
Bosch L J 2012 0.71 (0.52 - 0.9
Tang D 2011 0.58 (0.45 - 0.7
Li M 2009 0.58 (0.39 - 0.8
Itzkowitz S 2008 0.09 (0.01 - 1.2
Wang D-R and 0.41 (0.26 - 0.6
Zhang H 2014 0.52 (0.38 - 0.7
Zhang H 2014 0.59 (0.44 - 0.7
Nagasaka T 2009 0.92 (0.83 - 1.0
Nagasaka T 2009 0.74 (0.61 - 0.8
Tang D 2008 0.39 (0.24 - 0.6
Tang D 2008 0.46 (0.31 - 0.6
Random Effects Model
Pooled Negative LR = 0.73 (0.67 to 0.79)
Cochran-Q = 226.48; df = 42 (p = 0.0000)
Inconsistency (I-square) = 81.5 %
Tau-squared = 0.0516
0.01 1 100.0
Negative LR

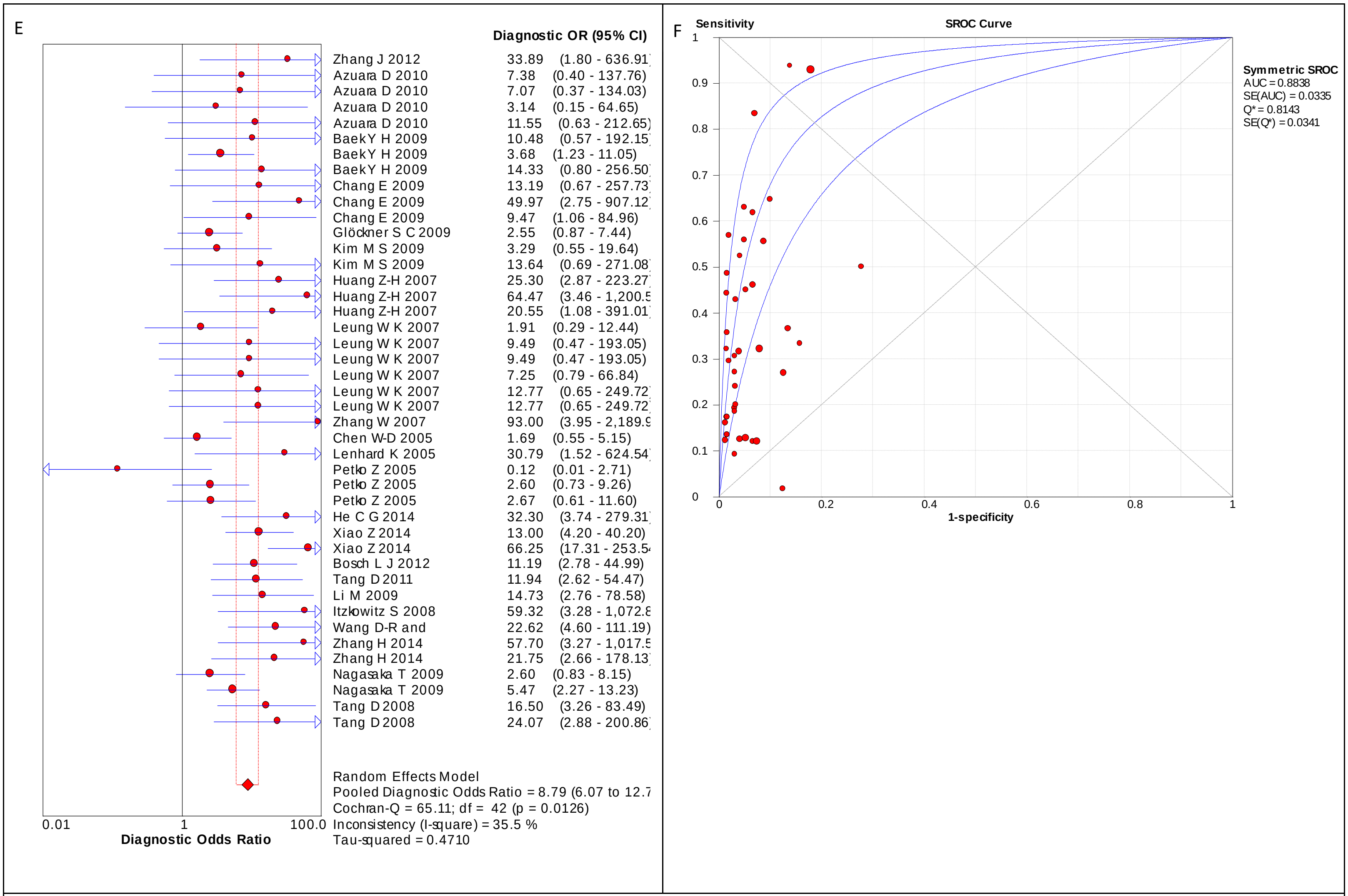

Figure S1.2. The summary performance of single-gene stool-based DNA methylation biomarker tests in all studies in TA (A) sensitivity, (B) specificity, (C) positive likelihood ratios, (D) negative likelihood ratios, (E) diagnostic odds ratio, (F) summary ROC curves.

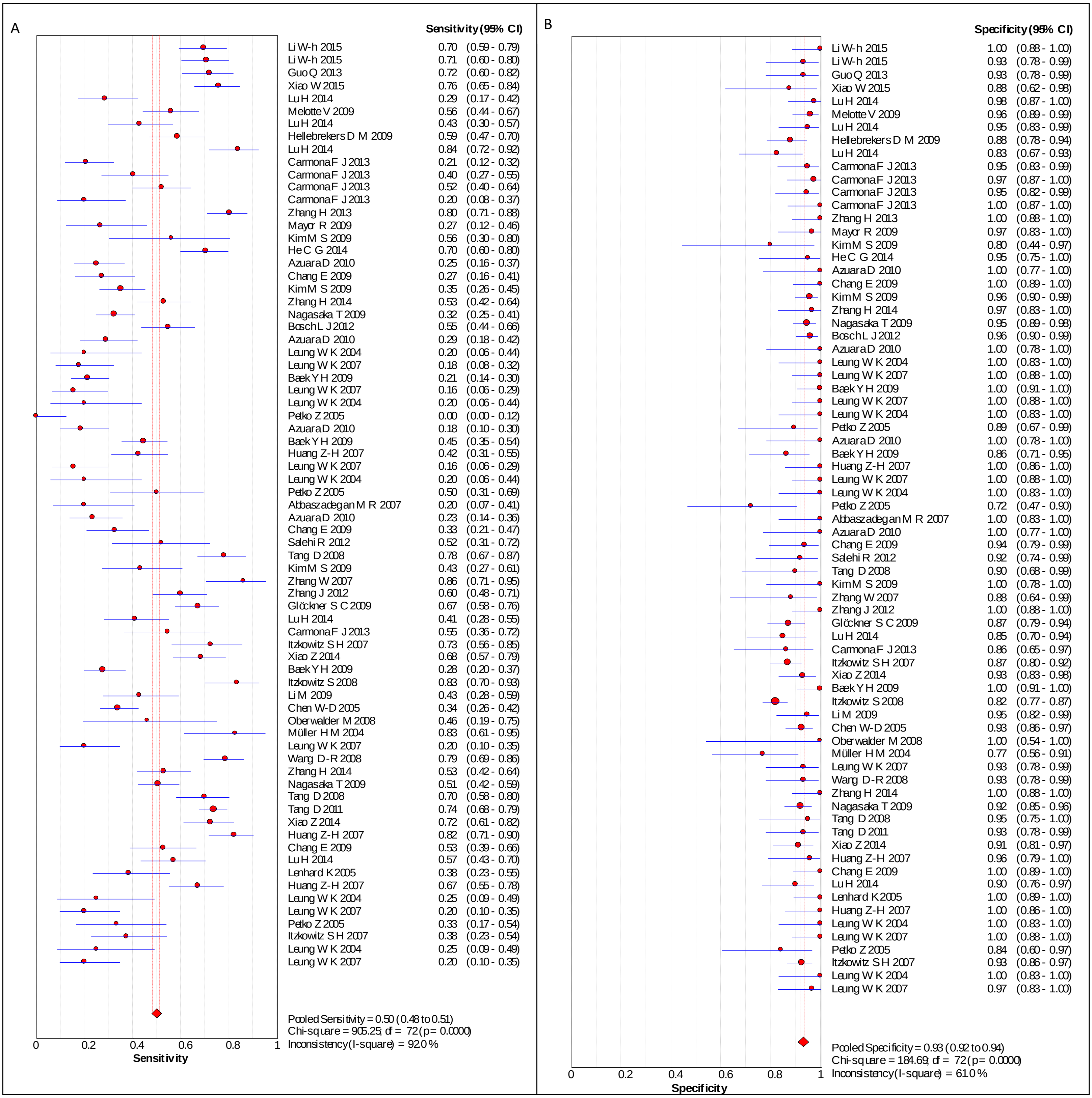
A
Sensitivity (95% CI)
Li W-h 2015 0.70 (0.59 - 0.79)
Li W-h 2015 0.71 (0.60 - 0.80)
Guo Q 2013 0.72 (0.60 - 0.82)
Xiao W 2015 0.76 (0.65 - 0.84)
Lu H 2014 0.29 (0.17 - 0.42)
Melotte V 2009 0.56 (0.44 - 0.67)
Lu H 2014 0.43 (0.30 - 0.57)
Hellebrekers D M 2009 0.59 (0.47 - 0.70)
Lu H 2014 0.84 (0.72 - 0.92)
Carmona F J 2013 0.21 (0.12 - 0.32)
Carmona F J 2013 0.40 (0.27 - 0.55)
Carmona F J 2013 0.52 (0.40 - 0.64)
Carmona F J 2013 0.20 (0.08 - 0.37)
Zhang H 2013 0.80 (0.71 - 0.88)
Mayor R 2009 0.27 (0.12 - 0.46)
Kim M S 2009 0.56 (0.30 - 0.80)
He C G 2014 0.70 (0.60 - 0.80)
Azuara D 2010 0.25 (0.16 - 0.37)
Chang E 2009 0.27 (0.16 - 0.41)
Kim M S 2009 0.35 (0.26 - 0.45)
Zhang H 2014 0.53 (0.42 - 0.64)
Nagasaka T 2009 0.32 (0.25 - 0.41)
Bosch L J 2012 0.55 (0.44 - 0.66)
Azuara D 2010 0.29 (0.18 - 0.42)
Leung W K 2004 0.20 (0.06 - 0.44)
Leung W K 2007 0.18 (0.08 - 0.32)
Baek Y H 2009 0.21 (0.14 - 0.30)
Leung W K 2007 0.16 (0.06 - 0.29)
Leung W K 2004 0.20 (0.06 - 0.44)
Petko Z 2005 0.00 (0.00 - 0.12)
Azuara D 2010 0.18 (0.10 - 0.30)
Baek Y H 2009 0.45 (0.35 - 0.54)
Huang Z-H 2007 0.42 (0.31 - 0.55)
Leung W K 2007 0.16 (0.06 - 0.29)
Leung W K 2004 0.20 (0.06 - 0.44)
Petko Z 2005 0.50 (0.31 - 0.69)
Abbaszadegan M R 2007 0.20 (0.07 - 0.41)
Azuara D 2010 0.23 (0.14 - 0.36)
Chang E 2009 0.33 (0.21 - 0.47)
Salehi R 2012 0.52 (0.31 - 0.72)
Tang D 2008 0.78 (0.67 - 0.87)
Kim M S 2009 0.43 (0.27 - 0.61)
Zhang W 2007 0.86 (0.71 - 0.95)
Zhang J 2012 0.60 (0.48 - 0.71)
Glöckner S C 2009 0.67 (0.58 - 0.76)
Lu H 2014 0.41 (0.28 - 0.55)
Carmona F J 2013 0.55 (0.36 - 0.72)
Itzkowitz S H 2007 0.73 (0.56 - 0.85)
Xiao Z 2014 0.68 (0.57 - 0.79)
Baek Y H 2009 0.28 (0.20 - 0.37)
Itzkowitz S 2008 0.83 (0.70 - 0.93)
Li M 2009 0.43 (0.28 - 0.59)
Chen W-D 2005 0.34 (0.26 - 0.42)
Oberwalder M 2008 0.46 (0.19 - 0.75)
Müller H M 2004 0.83 (0.61 - 0.95)
Leung W K 2007 0.20 (0.10 - 0.35)
Wang D-R 2008 0.79 (0.69 - 0.86)
Zhang H 2014 0.53 (0.42 - 0.64)
Nagasaka T 2009 0.51 (0.42 - 0.59)
Tang D 2008 0.70 (0.58 - 0.80)
Tang D 2011 0.74 (0.68 - 0.79)
Xiao Z 2014 0.72 (0.61 - 0.82)
Huang Z-H 2007 0.82 (0.71 - 0.90)
Chang E 2009 0.53 (0.39 - 0.66)
Lu H 2014 0.57 (0.43 - 0.70)
Lenhard K 2005 0.38 (0.23 - 0.55)
Huang Z-H 2007 0.67 (0.55 - 0.78)
Leung W K 2004 0.25 (0.09 - 0.49)
Leung W K 2007 0.20 (0.10 - 0.35)
Petko Z 2005 0.33 (0.17 - 0.54)
Itzkowitz S H 2007 0.38 (0.23 - 0.54)
Leung W K 2004 0.25 (0.09 - 0.49)
Leung W K 2007 0.20 (0.10 - 0.35)
Pooled Sensitivity = 0.50 (0.48 to 0.51)
Chi-square = 905.25; df = 72 (p = 0.0000)
Inconsistency (I-square) = 92.0 %
0 0.2 0.4 0.6 0.8 1
Sensitivity
B
Specificity (95% CI)
Li W-h 2015 1.00 (0.88 - 1.00)
Li W-h 2015 0.93 (0.78 - 0.99)
Guo Q 2013 0.93 (0.78 - 0.99)
Xiao W 2015 0.88 (0.62 - 0.98)
Lu H 2014 0.98 (0.87 - 1.00)
Melotte V 2009 0.96 (0.89 - 0.99)
Lu H 2014 0.95 (0.83 - 0.99)
Hellebrekers D M 2009 0.88 (0.78 - 0.94)
Lu H 2014 0.83 (0.67 - 0.93)
Carmona F J 2013 0.95 (0.83 - 0.99)
Carmona F J 2013 0.97 (0.87 - 1.00)
Carmona F J 2013 0.95 (0.82 - 0.99)
Carmona F J 2013 1.00 (0.87 - 1.00)
Zhang H 2013 1.00 (0.88 - 1.00)
Mayor R 2009 0.97 (0.83 - 1.00)
Kim M S 2009 0.80 (0.44 - 0.97)
He C G 2014 0.95 (0.75 - 1.00)
Azuara D 2010 1.00 (0.77 - 1.00)
Chang E 2009 1.00 (0.89 - 1.00)
Kim M S 2009 0.96 (0.90 - 0.99)
Zhang H 2014 0.97 (0.83 - 1.00)
Nagasaka T 2009 0.95 (0.89 - 0.98)
Bosch L J 2012 0.96 (0.90 - 0.99)
Azuara D 2010 1.00 (0.78 - 1.00)
Leung W K 2004 1.00 (0.83 - 1.00)
Leung W K 2007 1.00 (0.88 - 1.00)
Baek Y H 2009 1.00 (0.91 - 1.00)
Leung W K 2007 1.00 (0.88 - 1.00)
Leung W K 2004 1.00 (0.83 - 1.00)
Petko Z 2005 0.89 (0.67 - 0.99)
Azuara D 2010 1.00 (0.78 - 1.00)
Baek Y H 2009 0.86 (0.71 - 0.95)
Huang Z-H 2007 1.00 (0.86 - 1.00)
Leung W K 2007 1.00 (0.88 - 1.00)
Leung W K 2004 1.00 (0.83 - 1.00)
Petko Z 2005 0.72 (0.47 - 0.90)
Abbaszadegan M R 2007 1.00 (0.83 - 1.00)
Azuara D 2010 1.00 (0.77 - 1.00)
Chang E 2009 0.94 (0.79 - 0.99)
Salehi R 2012 0.92 (0.74 - 0.99)
Tang D 2008 0.90 (0.68 - 0.99)
Kim M S 2009 1.00 (0.78 - 1.00)
Zhang W 2007 0.88 (0.64 - 0.99)
Zhang J 2012 1.00 (0.88 - 1.00)
Glöckner S C 2009 0.87 (0.79 - 0.94)
Lu H 2014 0.85 (0.70 - 0.94)
Carmona F J 2013 0.86 (0.65 - 0.97)
Itzkowitz S H 2007 0.87 (0.80 - 0.92)
Xiao Z 2014 0.93 (0.83 - 0.98)
Baek Y H 2009 1.00 (0.91 - 1.00)
Itzkowitz S 2008 0.82 (0.77 - 0.87)
Li M 2009 0.95 (0.82 - 0.99)
Chen W-D 2005 0.93 (0.86 - 0.97)
Oberwalder M 2008 1.00 (0.54 - 1.00)
Müller H M 2004 0.77 (0.56 - 0.91)
Leung W K 2007 0.93 (0.78 - 0.99)
Wang D-R 2008 0.93 (0.78 - 0.99)
Zhang H 2014 1.00 (0.88 - 1.00)
Nagasaka T 2009 0.92 (0.85 - 0.96)
Tang D 2008 0.95 (0.75 - 1.00)
Tang D 2011 0.93 (0.78 - 0.99)
Xiao Z 2014 0.91 (0.81 - 0.97)
Huang Z-H 2007 0.96 (0.79 - 1.00)
Chang E 2009 1.00 (0.89 - 1.00)
Lu H 2014 0.90 (0.76 - 0.97)
Lenhard K 2005 1.00 (0.89 - 1.00)
Huang Z-H 2007 1.00 (0.86 - 1.00)
Leung W K 2004 1.00 (0.83 - 1.00)
Leung W K 2007 1.00 (0.88 - 1.00)
Petko Z 2005 0.84 (0.60 - 0.97)
Itzkowitz S H 2007 0.93 (0.86 - 0.97)
Leung W K 2004 1.00 (0.83 - 1.00)
Leung W K 2007 0.97 (0.83 - 1.00)
Pooled Specificity = 0.93 (0.92 to 0.94)
Chi-square = 184.69; df = 72 (p = 0.0000)
Inconsistency (I-square) = 61.0 %
0 0.2 0.4 0.6 0.8 1
Specificity

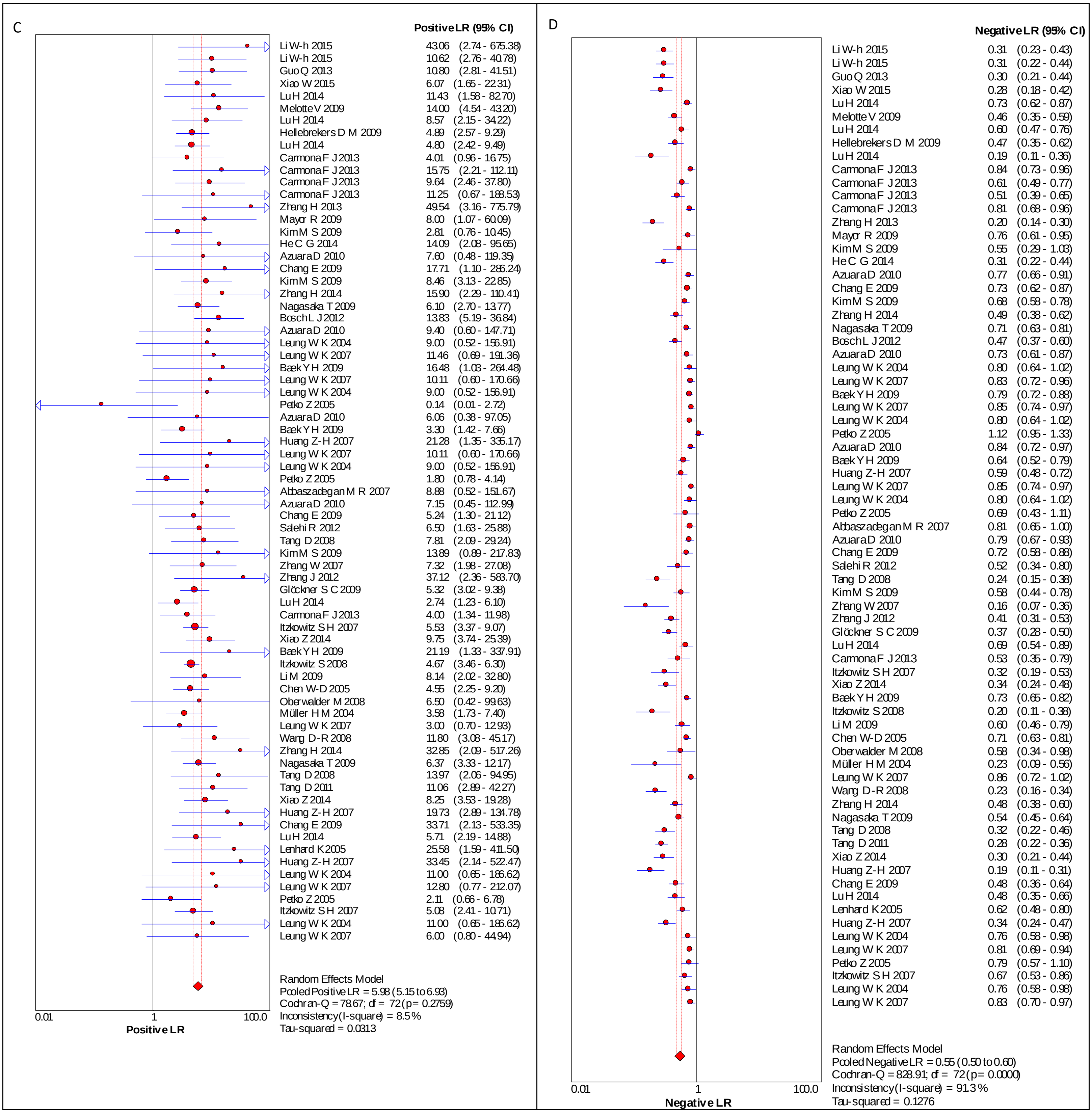
C
Positive LR (95% CI)
Li W-h 2015 43.06 (2.74 - 675.38)
Li W-h 2015 10.62 (2.76 - 40.78)
Guo Q 2013 10.80 (2.81 - 41.51)
Xiao W 2015 6.07 (1.65 - 22.31)
Lu H 2014 11.43 (1.58 - 82.70)
Melotte V 2009 14.00 (4.54 - 43.20)
Lu H 2014 8.57 (2.15 - 34.22)
Hellebrekers D M 2009 4.89 (2.57 - 9.29)
Lu H 2014 4.80 (2.42 - 9.49)
Carmona F J 2013 4.01 (0.96 - 16.75)
Carmona F J 2013 15.75 (2.21 - 112.11)
Carmona F J 2013 9.64 (2.46 - 37.80)
Carmona F J 2013 11.25 (0.67 - 188.53)
Zhang H 2013 49.54 (3.16 - 775.79)
Mayor R 2009 8.00 (1.07 - 60.09)
Kim M S 2009 2.81 (0.76 - 10.45)
He C G 2014 14.09 (2.08 - 95.65)
Azuara D 2010 7.60 (0.48 - 119.35)
Chang E 2009 17.71 (1.10 - 286.24)
Kim M S 2009 8.46 (3.13 - 22.85)
Zhang H 2014 15.90 (2.29 - 110.41)
Nagasaka T 2009 6.10 (2.70 - 13.77)
Bosch L J 2012 13.83 (5.19 - 36.84)
Azuara D 2010 9.40 (0.60 - 147.71)
Leung W K 2004 9.00 (0.52 - 156.91)
Leung W K 2007 11.46 (0.69 - 191.36)
Baek Y H 2009 16.48 (1.03 - 264.48)
Leung W K 2007 10.11 (0.60 - 170.66)
Leung W K 2004 9.00 (0.52 - 156.91)
Petko Z 2005 0.14 (0.01 - 2.72)
Azuara D 2010 6.06 (0.38 - 97.05)
Baek Y H 2009 3.30 (1.42 - 7.66)
Huang Z-H 2007 21.28 (1.35 - 335.17)
Leung W K 2007 10.11 (0.60 - 170.66)
Leung W K 2004 9.00 (0.52 - 156.91)
Petko Z 2005 1.80 (0.78 - 4.14)
Abbaszadegan M R 2007 8.88 (0.52 - 151.67)
Azuara D 2010 7.15 (0.45 - 112.99)
Chang E 2009 5.24 (1.30 - 21.12)
Salehi R 2012 6.50 (1.63 - 25.88)
Tang D 2008 7.81 (2.09 - 29.24)
Kim M S 2009 13.89 (0.89 - 217.83)
Zhang W 2007 7.32 (1.98 - 27.08)
Zhang J 2012 37.12 (2.36 - 583.70)
Glöckner S C 2009 5.32 (3.02 - 9.38)
Lu H 2014 2.74 (1.23 - 6.10)
Carmona F J 2013 4.00 (1.34 - 11.98)
Itzkowitz S H 2007 5.53 (3.37 - 9.07)
Xiao Z 2014 9.75 (3.74 - 25.39)
Baek Y H 2009 21.19 (1.33 - 337.91)
Itzkowitz S 2008 4.67 (3.46 - 6.30)
Li M 2009 8.14 (2.02 - 32.80)
Chen W-D 2005 4.55 (2.25 - 9.20)
Oberwalder M 2008 6.50 (0.42 - 99.63)
Müller H M 2004 3.58 (1.73 - 7.40)
Leung W K 2007 3.00 (0.70 - 12.93)
Wang D-R 2008 11.80 (3.08 - 45.17)
Zhang H 2014 32.85 (2.09 - 517.26)
Nagasaka T 2009 6.37 (3.33 - 12.17)
Tang D 2008 13.97 (2.06 - 94.95)
Tang D 2011 11.06 (2.89 - 42.27)
Xiao Z 2014 8.25 (3.53 - 19.28)
Huang Z-H 2007 19.73 (2.89 - 134.78)
Chang E 2009 33.71 (2.13 - 533.35)
Lu H 2014 5.71 (2.19 - 14.88)
Lenhard K 2005 25.58 (1.59 - 411.50)
Huang Z-H 2007 33.45 (2.14 - 522.47)
Leung W K 2004 11.00 (0.65 - 186.62)
Leung W K 2007 12.80 (0.77 - 212.07)
Petko Z 2005 2.11 (0.66 - 6.78)
Itzkowitz S H 2007 5.08 (2.41 - 10.71)
Leung W K 2004 11.00 (0.65 - 186.62)
Leung W K 2007 6.00 (0.80 - 44.94)
Random Effects Model
Pooled Positive LR = 5.98 (5.15 to 6.93)
Cochran-Q = 78.67; df = 72 (p = 0.2759)
Inconsistency (I-square) = 8.5 %
Tau-squared = 0.0313
0.01
1
100.0
Positive LR
D
Negative LR (95% CI)
Li W-h 2015 0.31 (0.23 - 0.43)
Li W-h 2015 0.31 (0.22 - 0.44)
Guo Q 2013 0.30 (0.21 - 0.44)
Xiao W 2015 0.28 (0.18 - 0.42)
Lu H 2014 0.73 (0.62 - 0.87)
Melotte V 2009 0.46 (0.35 - 0.59)
Lu H 2014 0.60 (0.47 - 0.76)
Hellebrekers D M 2009 0.47 (0.35 - 0.62)
Lu H 2014 0.19 (0.11 - 0.36)
Carmona F J 2013 0.84 (0.73 - 0.96)
Carmona F J 2013 0.61 (0.49 - 0.77)
Carmona F J 2013 0.51 (0.39 - 0.65)
Carmona F J 2013 0.81 (0.68 - 0.96)
Zhang H 2013 0.20 (0.14 - 0.30)
Mayor R 2009 0.76 (0.61 - 0.95)
Kim M S 2009 0.55 (0.29 - 1.03)
He C G 2014 0.31 (0.22 - 0.44)
Azuara D 2010 0.77 (0.66 - 0.91)
Chang E 2009 0.73 (0.62 - 0.87)
Kim M S 2009 0.68 (0.58 - 0.78)
Zhang H 2014 0.49 (0.38 - 0.62)
Nagasaka T 2009 0.71 (0.63 - 0.81)
Bosch L J 2012 0.47 (0.37 - 0.60)
Azuara D 2010 0.73 (0.61 - 0.87)
Leung W K 2004 0.80 (0.64 - 1.02)
Leung W K 2007 0.83 (0.72 - 0.96)
Baek Y H 2009 0.79 (0.72 - 0.88)
Leung W K 2007 0.85 (0.74 - 0.97)
Leung W K 2004 0.80 (0.64 - 1.02)
Petko Z 2005 1.12 (0.95 - 1.33)
Azuara D 2010 0.84 (0.72 - 0.97)
Baek Y H 2009 0.64 (0.52 - 0.79)
Huang Z-H 2007 0.59 (0.48 - 0.72)
Leung W K 2007 0.85 (0.74 - 0.97)
Leung W K 2004 0.80 (0.64 - 1.02)
Petko Z 2005 0.69 (0.43 - 1.11)
Abbaszadegan M R 2007 0.81 (0.65 - 1.00)
Azuara D 2010 0.79 (0.67 - 0.93)
Chang E 2009 0.72 (0.58 - 0.88)
Salehi R 2012 0.52 (0.34 - 0.80)
Tang D 2008 0.24 (0.15 - 0.38)
Kim M S 2009 0.58 (0.44 - 0.78)
Zhang W 2007 0.16 (0.07 - 0.36)
Zhang J 2012 0.41 (0.31 - 0.53)
Glöckner S C 2009 0.37 (0.28 - 0.50)
Lu H 2014 0.69 (0.54 - 0.89)
Carmona F J 2013 0.53 (0.35 - 0.79)
Itzkowitz S H 2007 0.32 (0.19 - 0.53)
Xiao Z 2014 0.34 (0.24 - 0.48)
Baek Y H 2009 0.73 (0.65 - 0.82)
Itzkowitz S 2008 0.20 (0.11 - 0.38)
Li M 2009 0.60 (0.46 - 0.79)
Chen W-D 2005 0.71 (0.63 - 0.81)
Oberwalder M 2008 0.58 (0.34 - 0.98)
Müller H M 2004 0.23 (0.09 - 0.56)
Leung W K 2007 0.86 (0.72 - 1.02)
Wang D-R 2008 0.23 (0.16 - 0.34)
Zhang H 2014 0.48 (0.38 - 0.60)
Nagasaka T 2009 0.54 (0.45 - 0.64)
Tang D 2008 0.32 (0.22 - 0.46)
Tang D 2011 0.28 (0.22 - 0.36)
Xiao Z 2014 0.30 (0.21 - 0.44)
Huang Z-H 2007 0.19 (0.11 - 0.31)
Chang E 2009 0.48 (0.36 - 0.64)
Lu H 2014 0.48 (0.35 - 0.66)
Lenhard K 2005 0.62 (0.48 - 0.80)
Huang Z-H 2007 0.34 (0.24 - 0.47)
Leung W K 2004 0.76 (0.58 - 0.98)
Leung W K 2007 0.81 (0.69 - 0.94)
Petko Z 2005 0.79 (0.57 - 1.10)
Itzkowitz S H 2007 0.67 (0.53 - 0.86)
Leung W K 2004 0.76 (0.58 - 0.98)
Leung W K 2007 0.83 (0.70 - 0.97)
Random Effects Model
Pooled Negative LR = 0.55 (0.50 to 0.60)
Cochran-Q = 828.91; df = 72 (p = 0.0000)
Inconsistency (I-square) = 91.3 %
Tau-squared = 0.1276
0.01
1
100.0
Negative LR

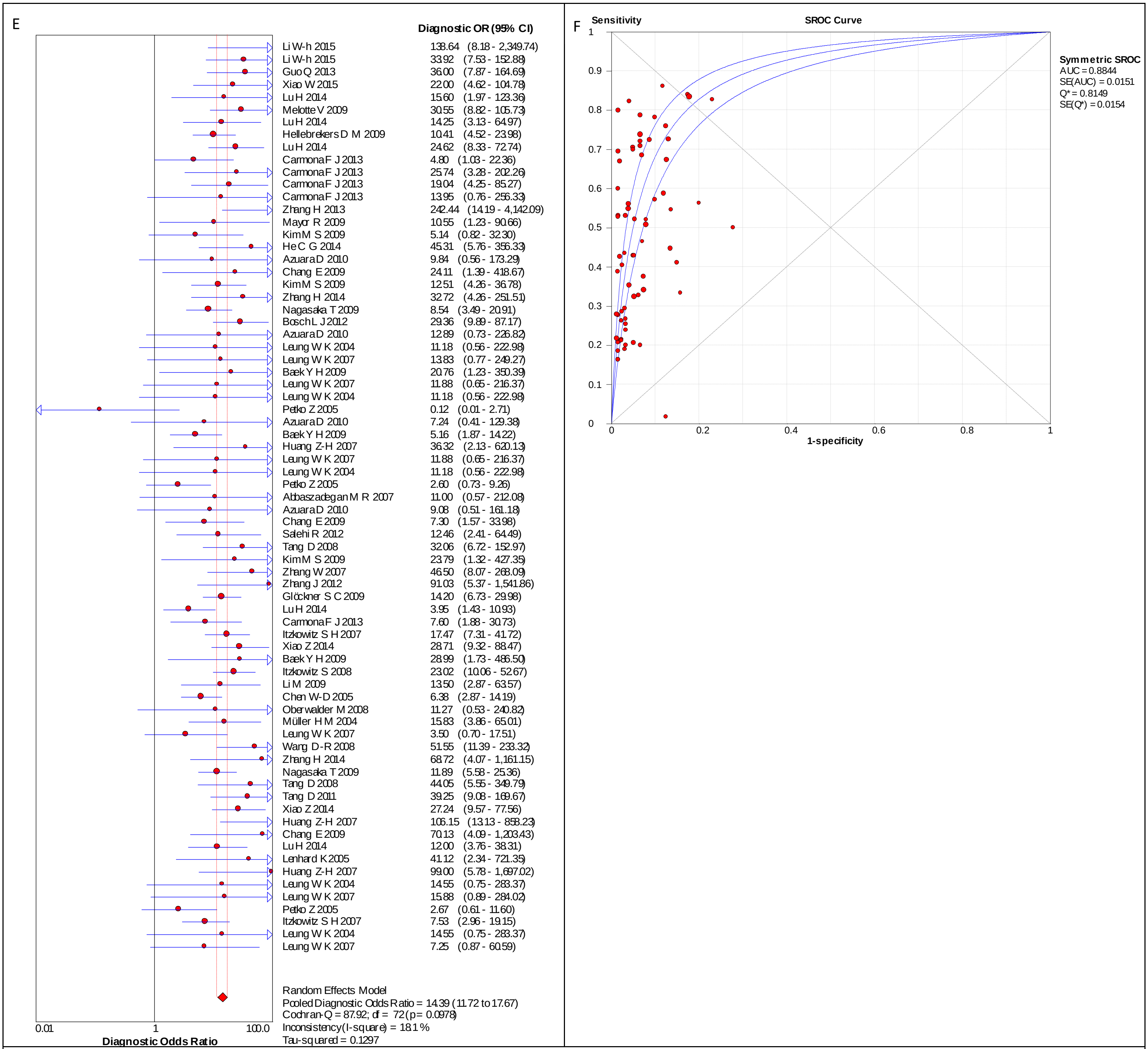

Figure S1.3. The summary performance of single-gene stool-based DNA methylation biomarker tests in all studies in TP (A) sensitivity, (B) specificity, (C) positive likelihood ratios, (D) negative likelihood ratios, (E) diagnostic odds ratio, (F) summary ROC curves.
