# Supplementary material for: The importance of stool DNA methylation in colorectal cancer diagnosis: A meta-analysis
Source: PLoS One. 2018 Jul 19;13(7):e0200735. doi: 10.1371/journal.pone.0200735 (PMC6053185; doi:10.1371/journal.pone.0200735)
Supplement: S2 File — (PDF) [file pone.0200735.s002.pdf]

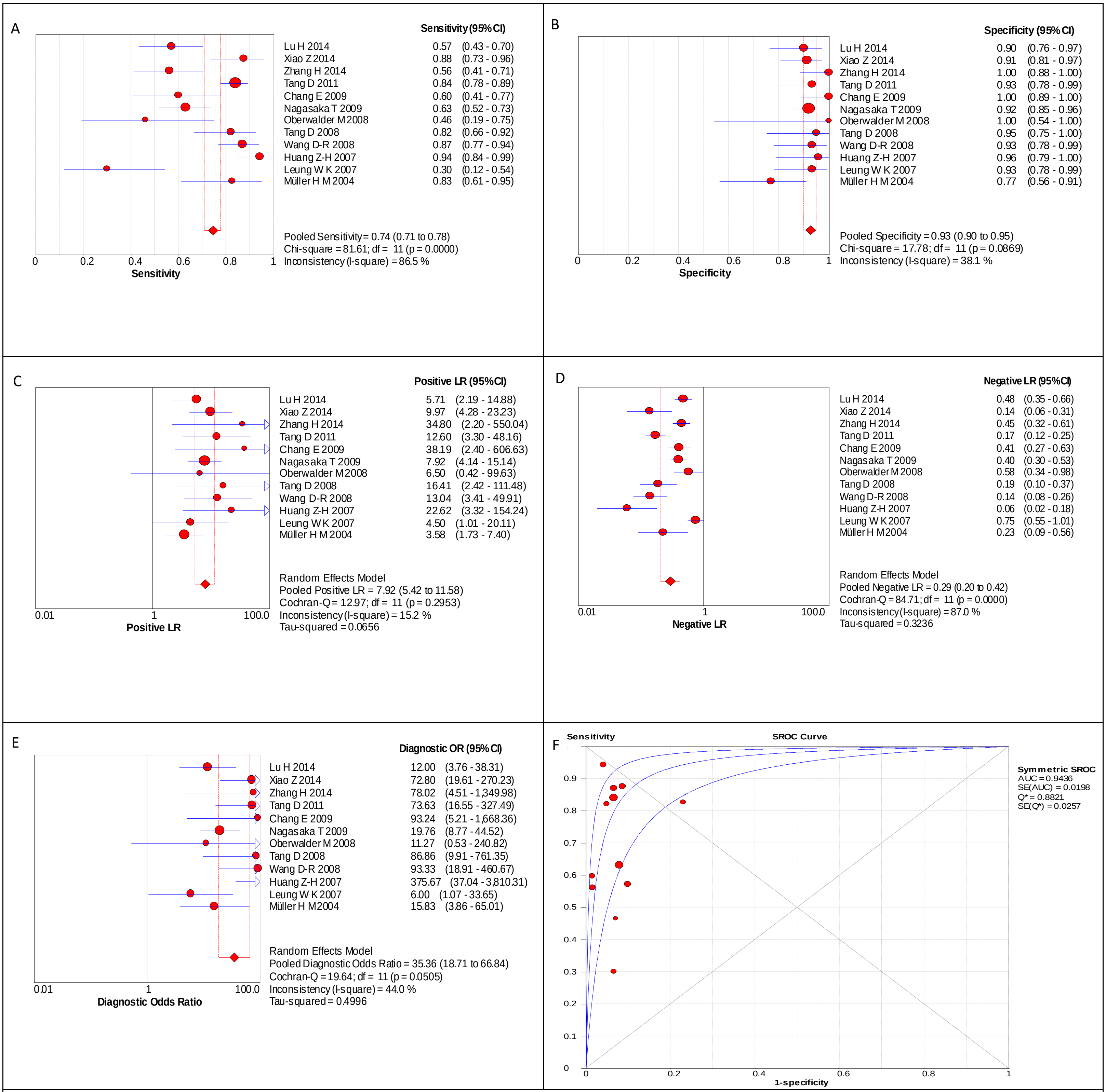

Figure S2.1. The summary of *SFRP2* gene in CRC (A) sensitivity, (B) specificity, (C) positive likelihood ratios, (D) negative likelihood ratios, (E) diagnostic odds ratio, (F) summary ROC curves.

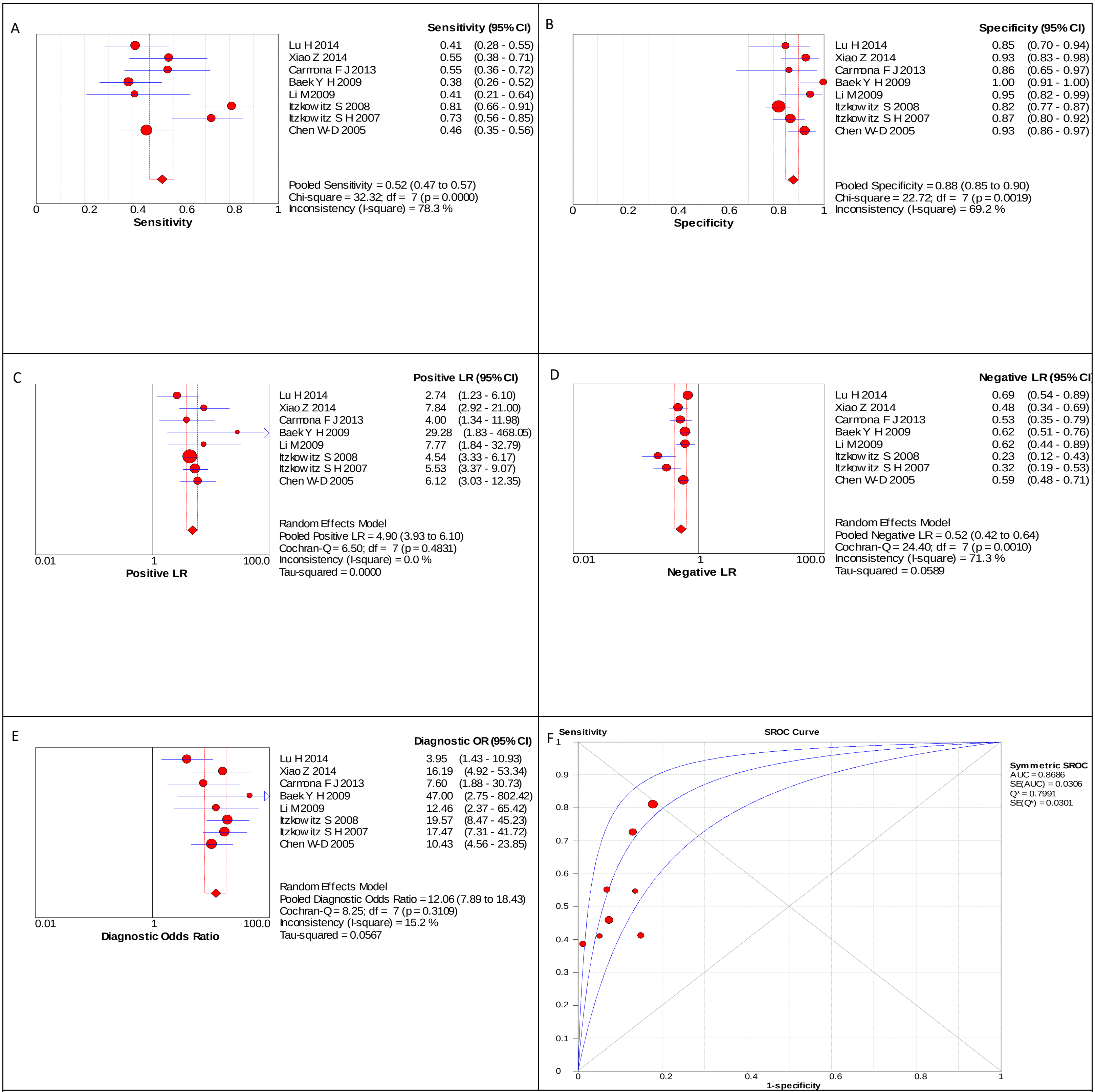

Figure S2.2. The summary of *VIM* gene in CRC (A) sensitivity, (B) specificity, (C) positive likelihood ratios, (D) negative likelihood ratios, (E) diagnostic odds ratio, (F) summary ROC curves.

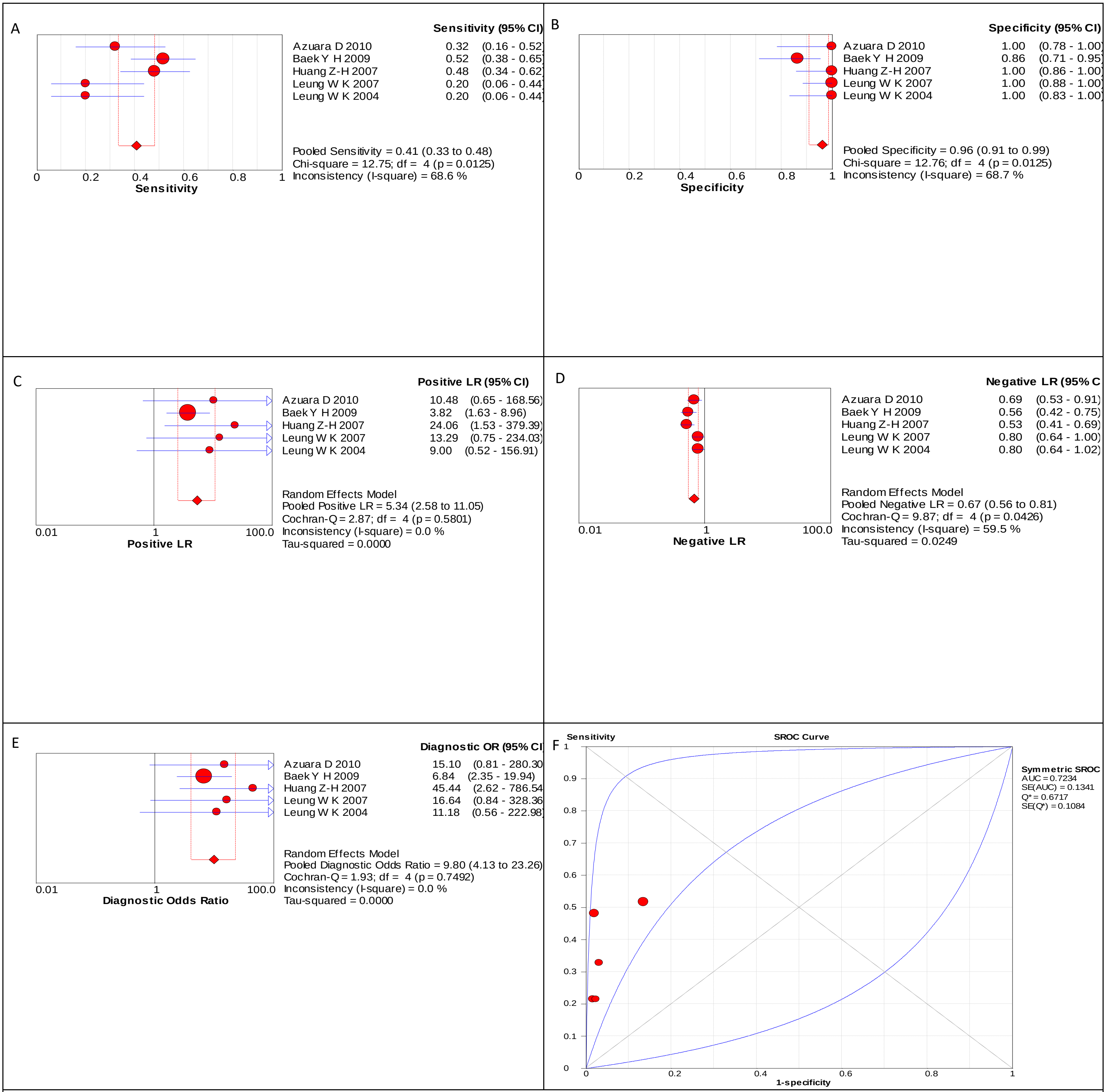

Figure S2.3. The summary of *MGMT* gene in CRC (A) sensitivity, (B) specificity, (C) positive likelihood ratios, (D) negative likelihood ratios, (E) diagnostic odds ratio, (F) summary ROC curves.

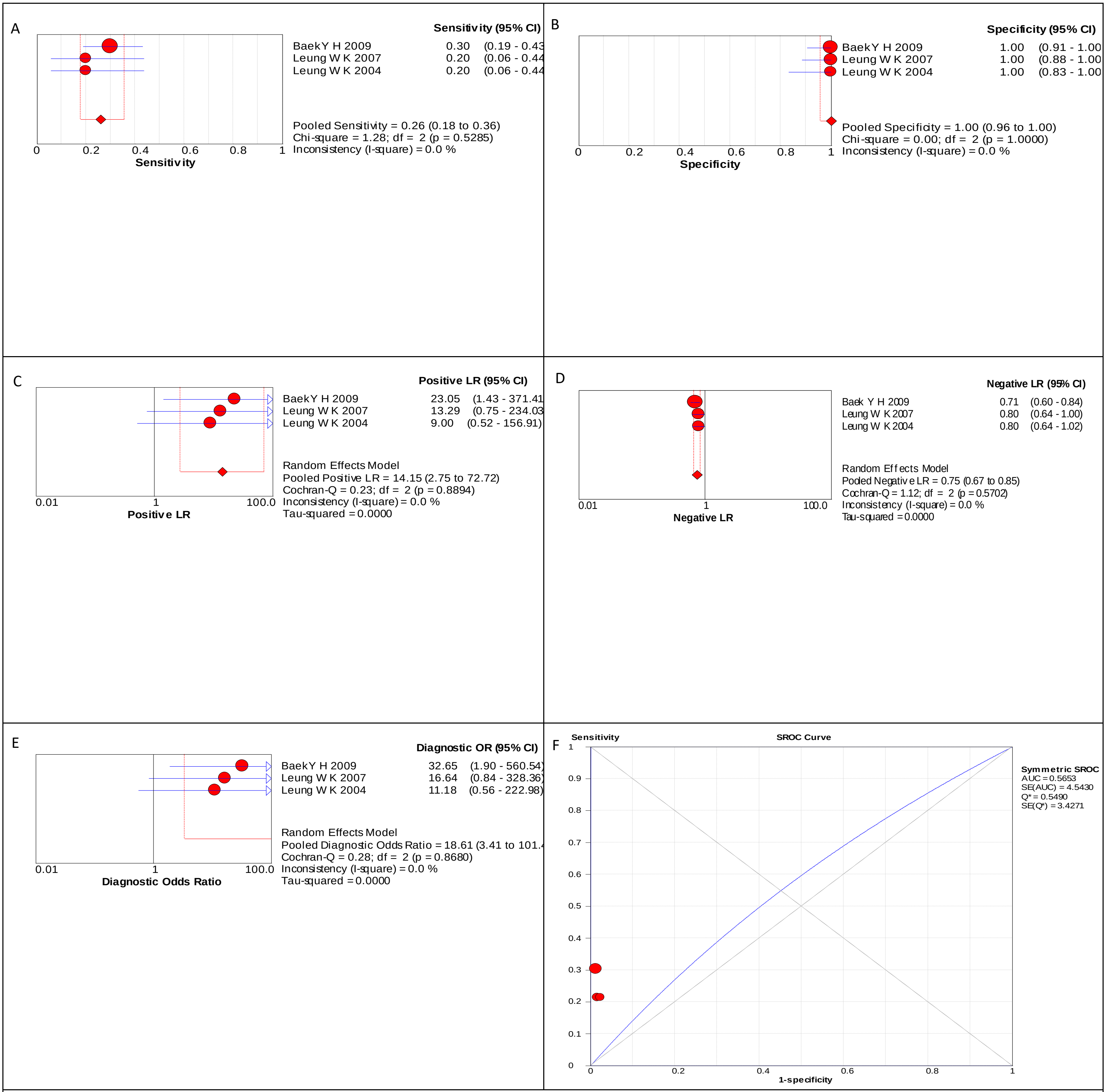

Figure S2.4. The summary of *MLH1* gene in CRC (A) sensitivity, (B) specificity, (C) positive likelihood ratios, (D) negative likelihood ratios, (E) diagnostic odds ratio, (F) summary ROC curves.

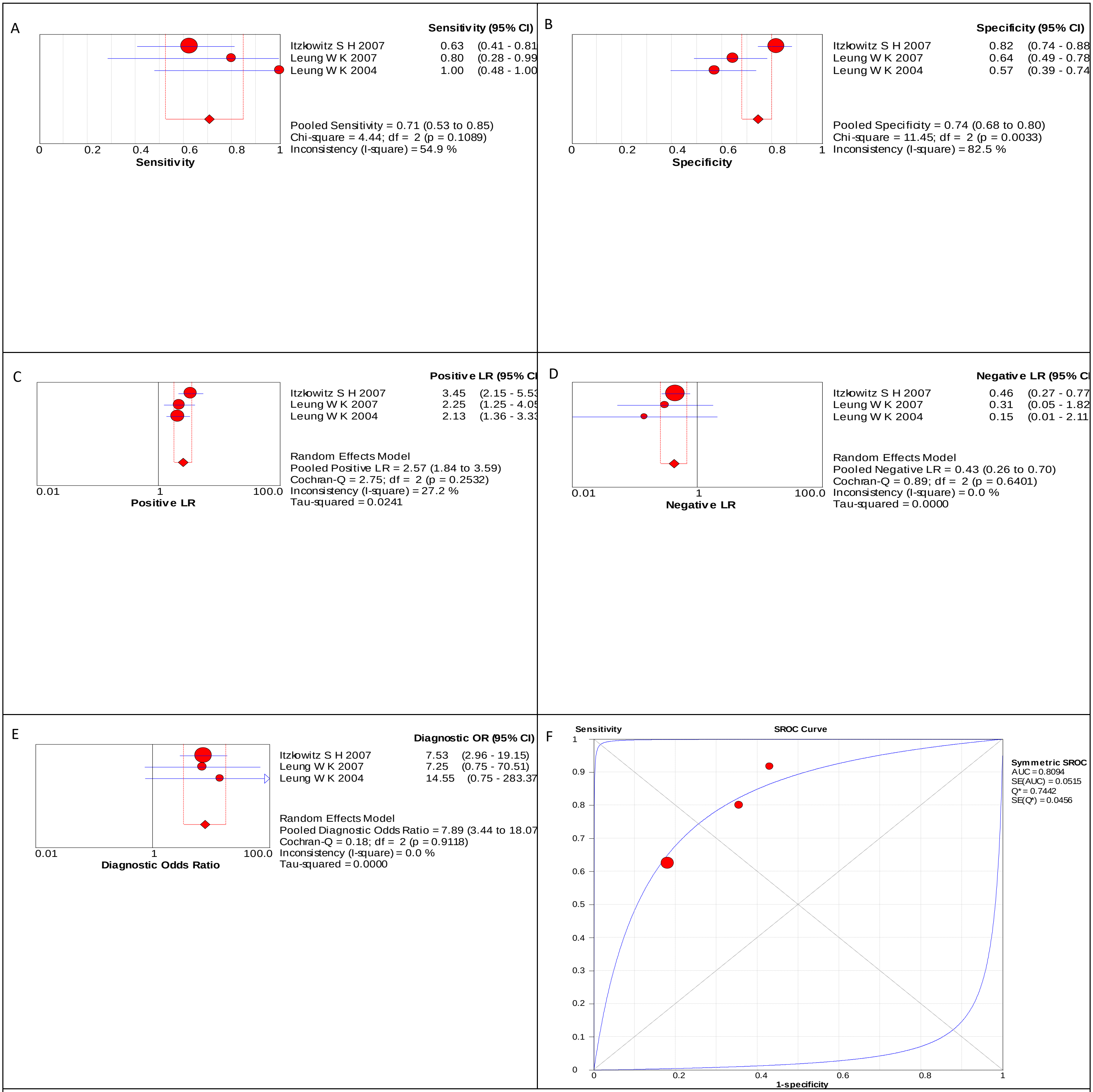

Figure S2.5. The summary *HLTF of* gene in CRC (A) sensitivity, (B) specificity, (C) positive likelihood ratios, (D) negative likelihood ratios, (E) diagnostic odds ratio, (F) summary ROC curves.

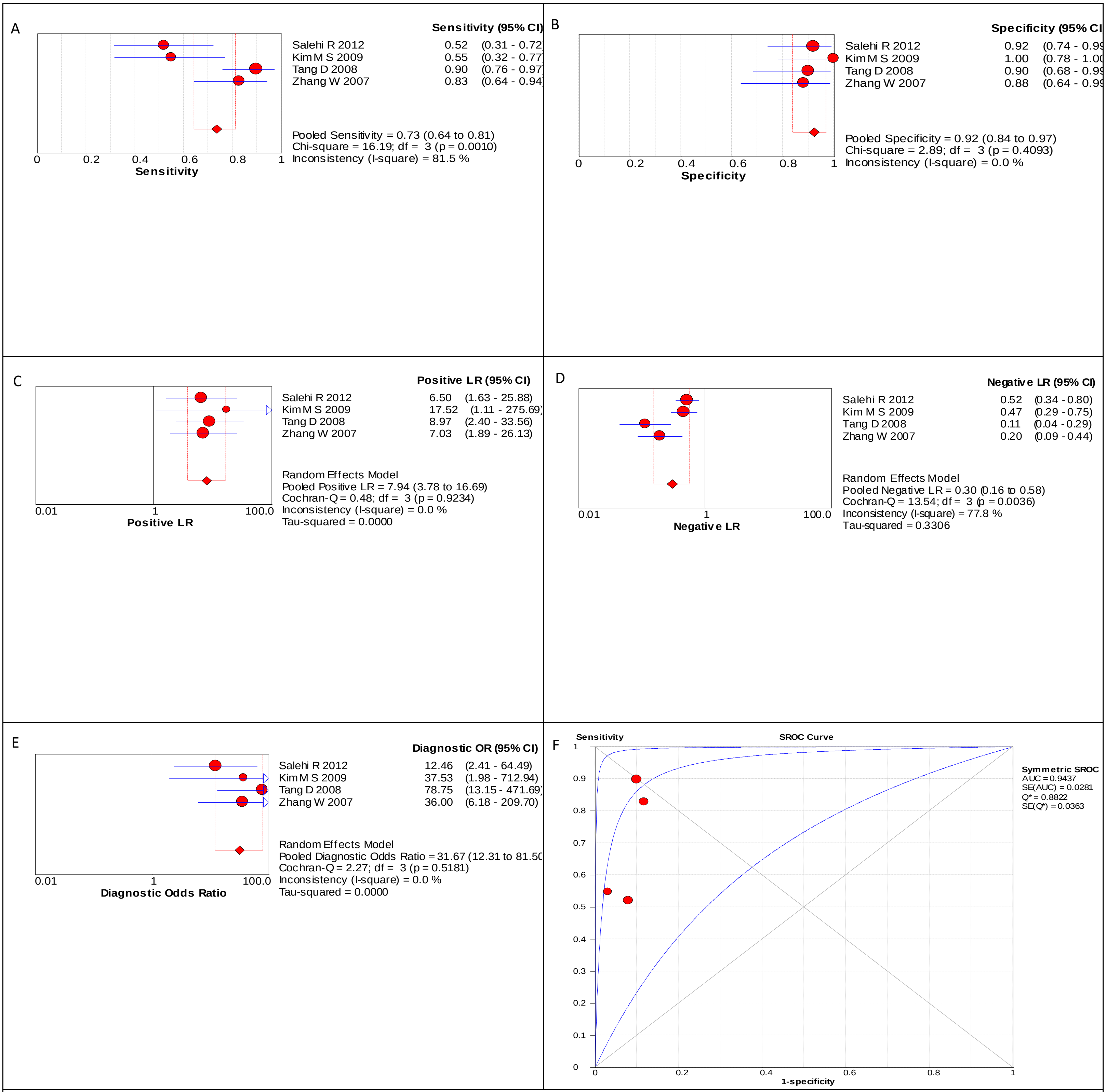

Figure S2.6. The summary *SFRP1 of* gene in CRC (A) sensitivity, (B) specificity, (C) positive likelihood ratios, (D) negative likelihood ratios, (E) diagnostic odds ratio, (F) summary ROC curves.

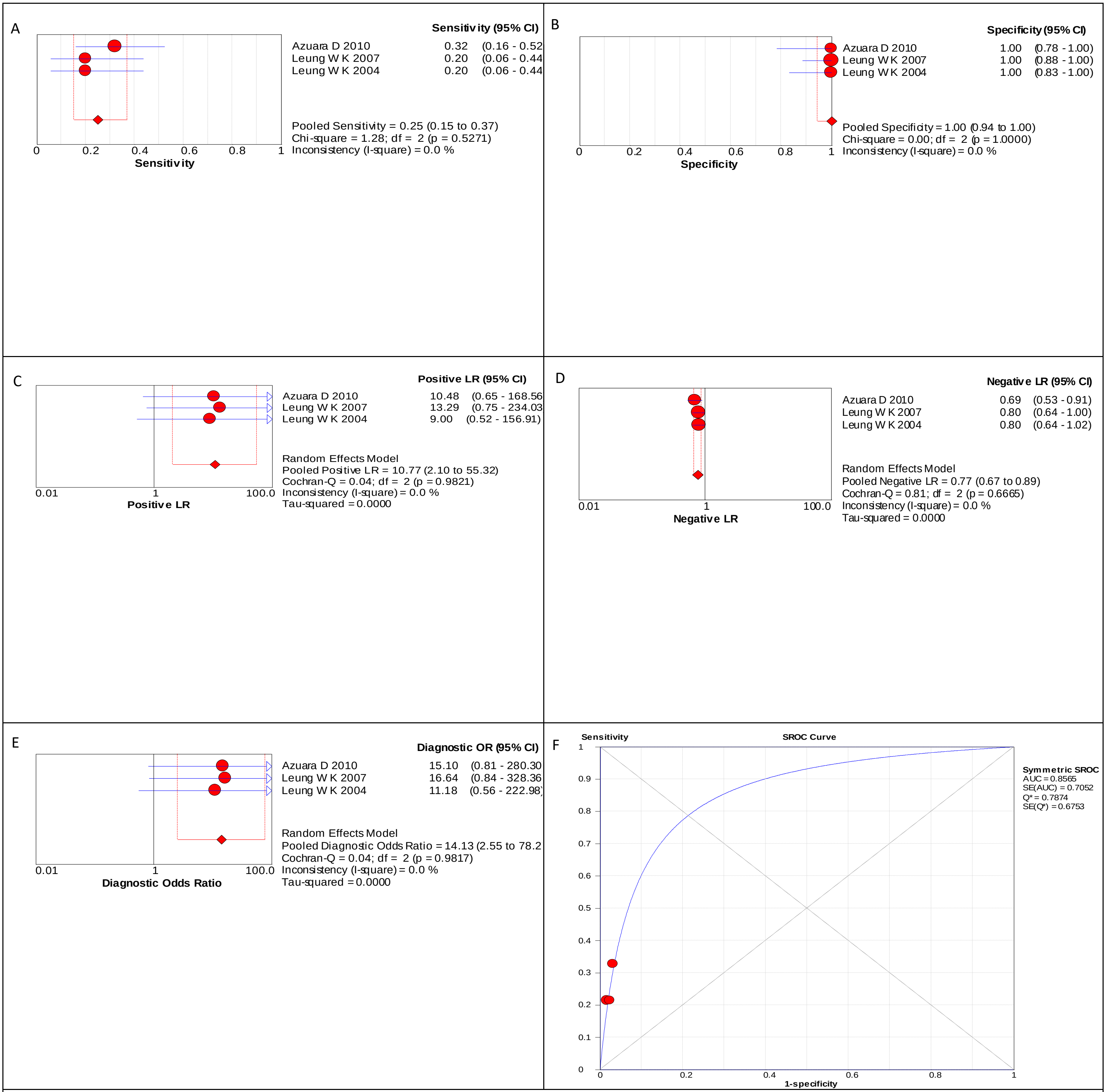

Figure S2.7. The summary *APC of* gene in CRC (A) sensitivity, (B) specificity, (C) positive likelihood ratios, (D) negative likelihood ratios, (E) diagnostic odds ratio, (F) summary ROC curves.

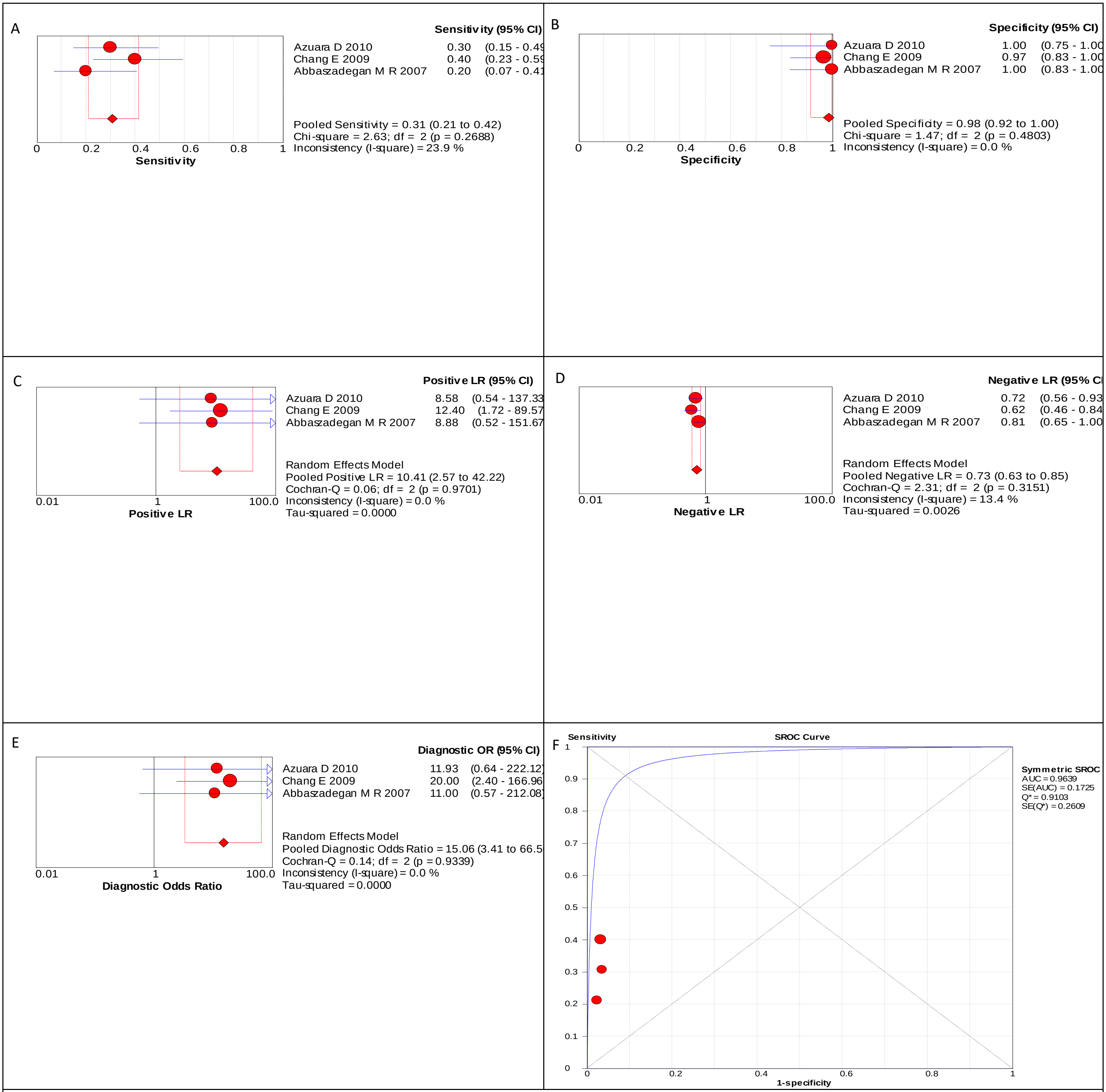

Figure S2.8. The summary *P16 of* gene in CRC (A) sensitivity, (B) specificity, (C) positive likelihood ratios, (D) negative likelihood ratios, (E) diagnostic odds ratio, (F) summary ROC curves.

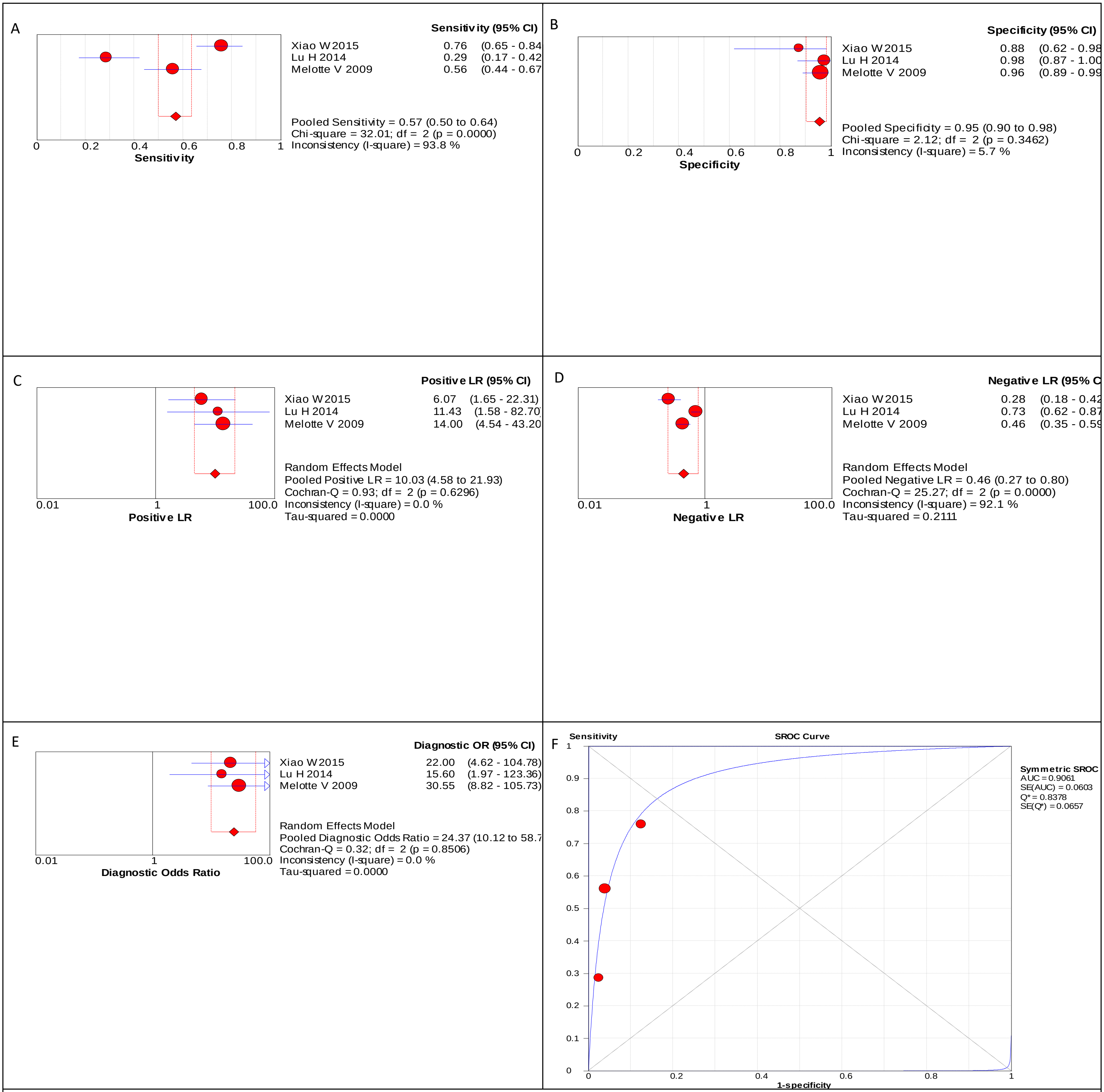

Figure S2.9. The summary *NDRG4 of* gene in CRC (A) sensitivity, (B) specificity, (C) positive likelihood ratios, (D) negative likelihood ratios, (E) diagnostic odds ratio, (F) summary ROC curves.

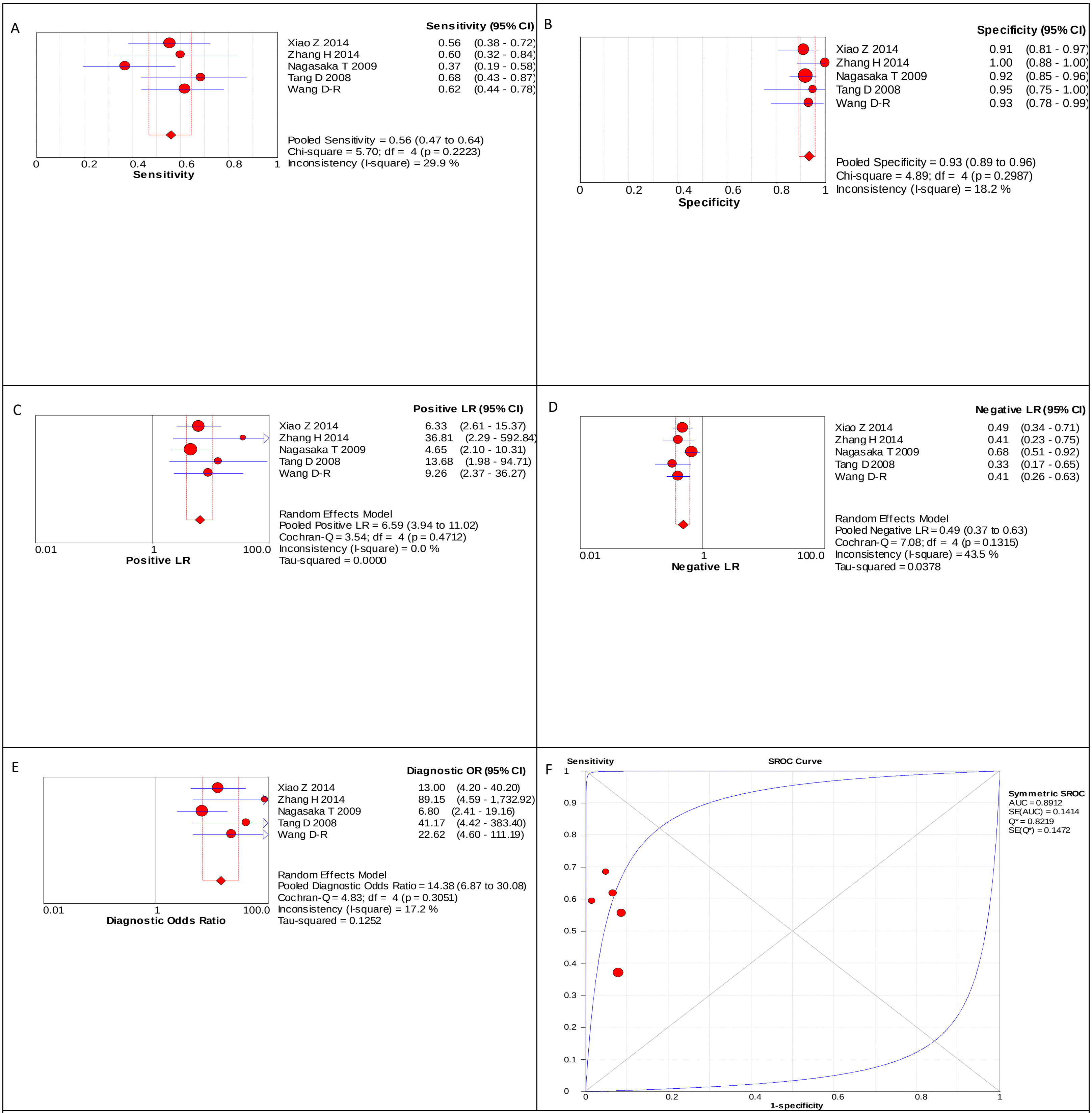

Figure S2.10. The summary *SFRP2 of* gene in AA (A) sensitivity, (B) specificity, (C) positive likelihood ratios, (D) negative likelihood ratios, (E) diagnostic odds ratio, (F) summary ROC curves.

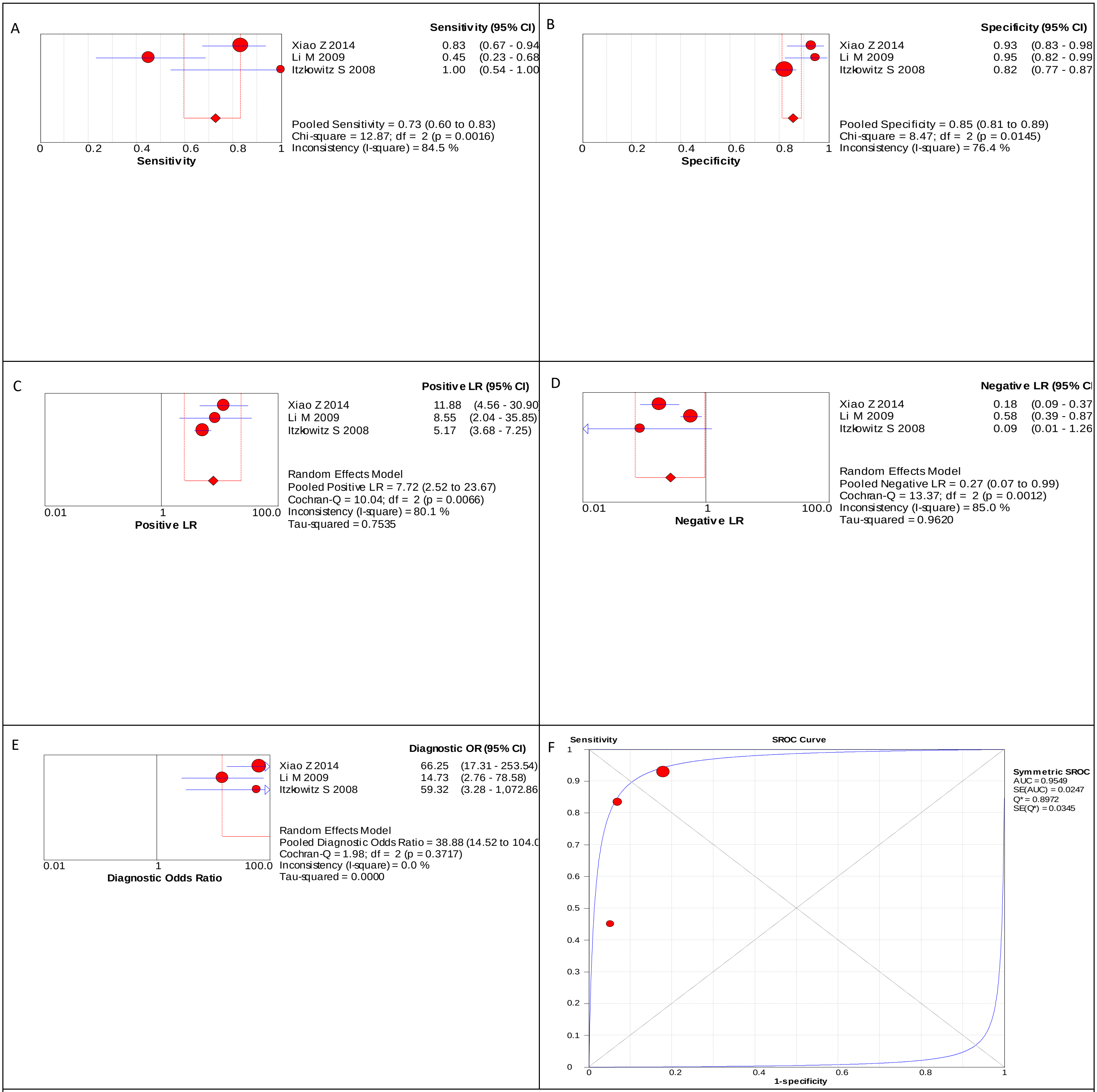

Figure S2.11. The summary *VIM of* gene in AA (A) sensitivity, (B) specificity, (C) positive likelihood ratios, (D) negative likelihood ratios, (E) diagnostic odds ratio, (F) summary ROC curves.

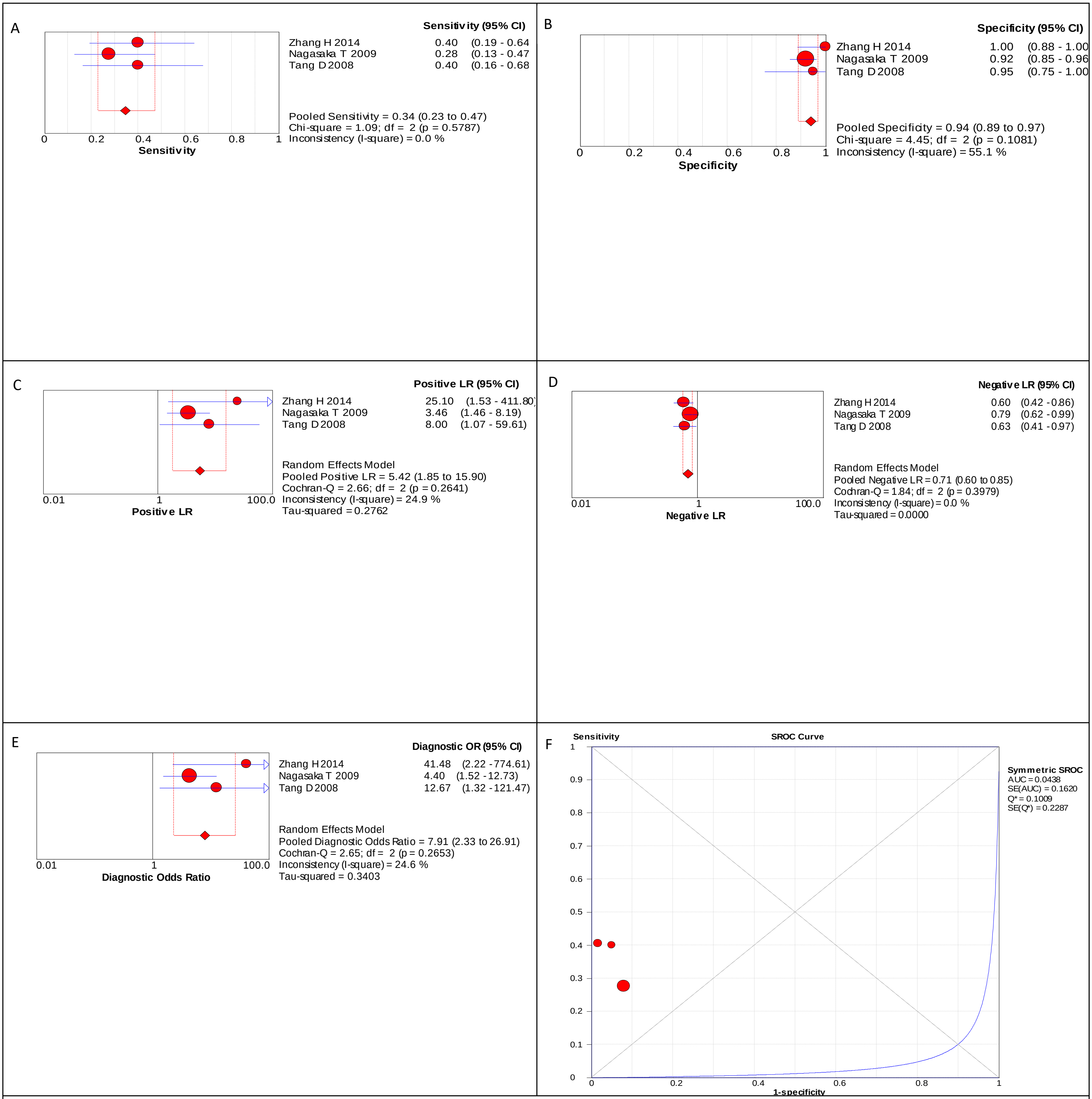

Figure S2.12. The summary *SFRP2 of* gene in NAA (A) sensitivity, (B) specificity, (C) positive likelihood ratios, (D) negative likelihood ratios, (E) diagnostic odds ratio, (F) summary ROC curves.

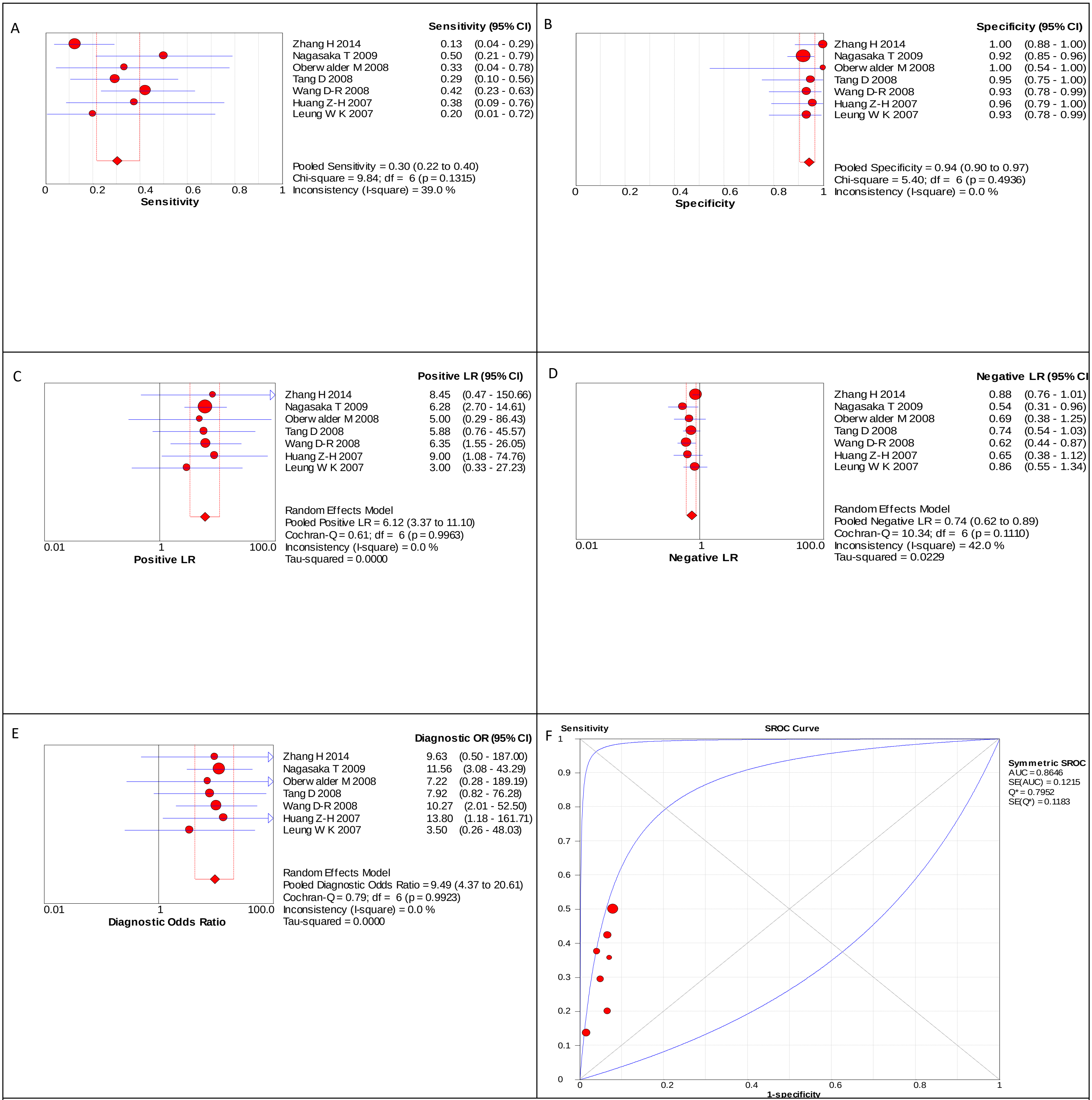

Figure S2.13. The summary *SFRP2 of* gene in HP (A) sensitivity, (B) specificity, (C) positive likelihood ratios, (D) negative likelihood ratios, (E) diagnostic odds ratio, (F) summary ROC curves.

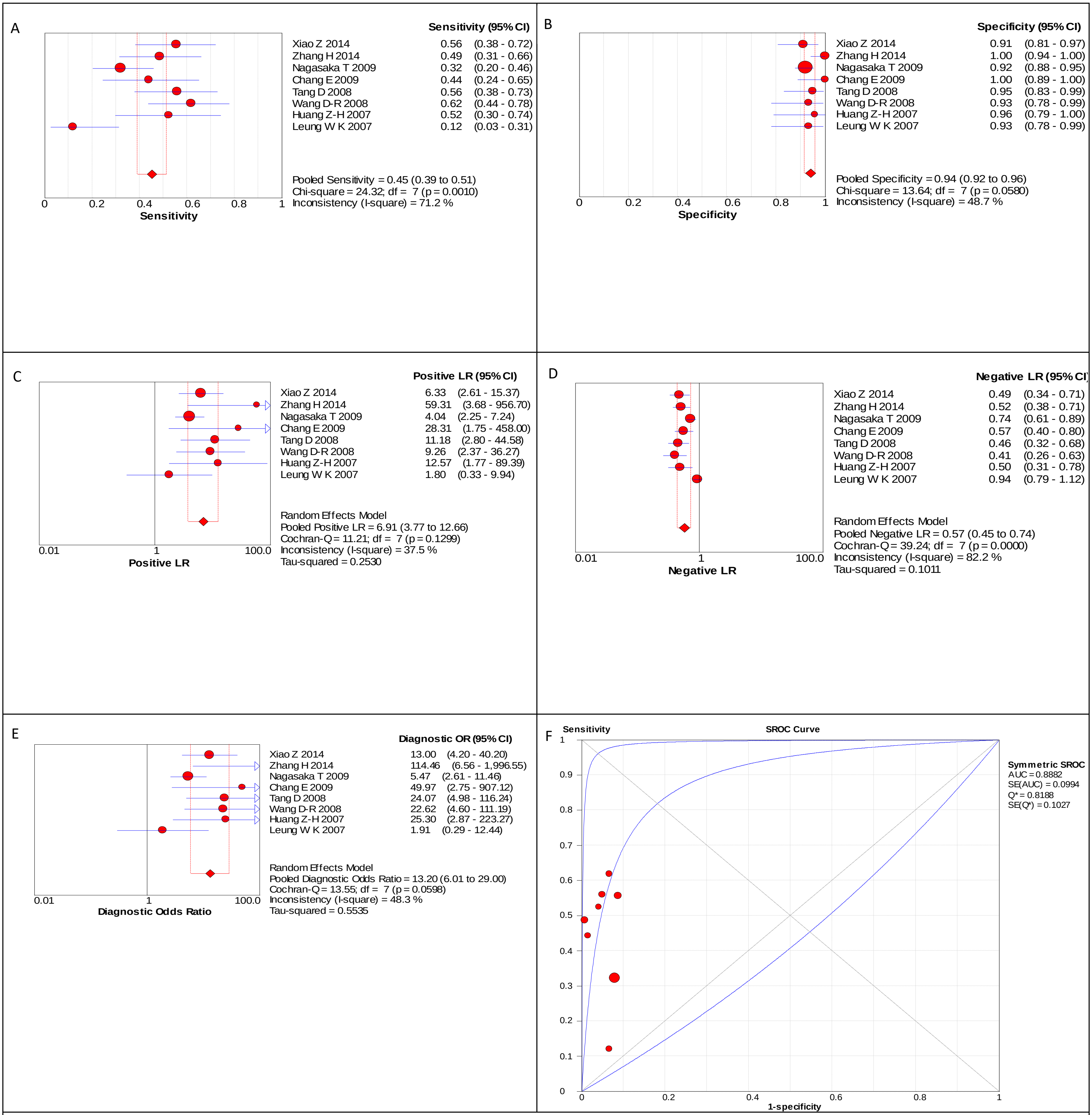

Figure S2.14. The summary *SFRP2 of* gene in TA (A) sensitivity, (B) specificity, (C) positive likelihood ratios, (D) negative likelihood ratios, (E) diagnostic odds ratio, (F) summary ROC curves.

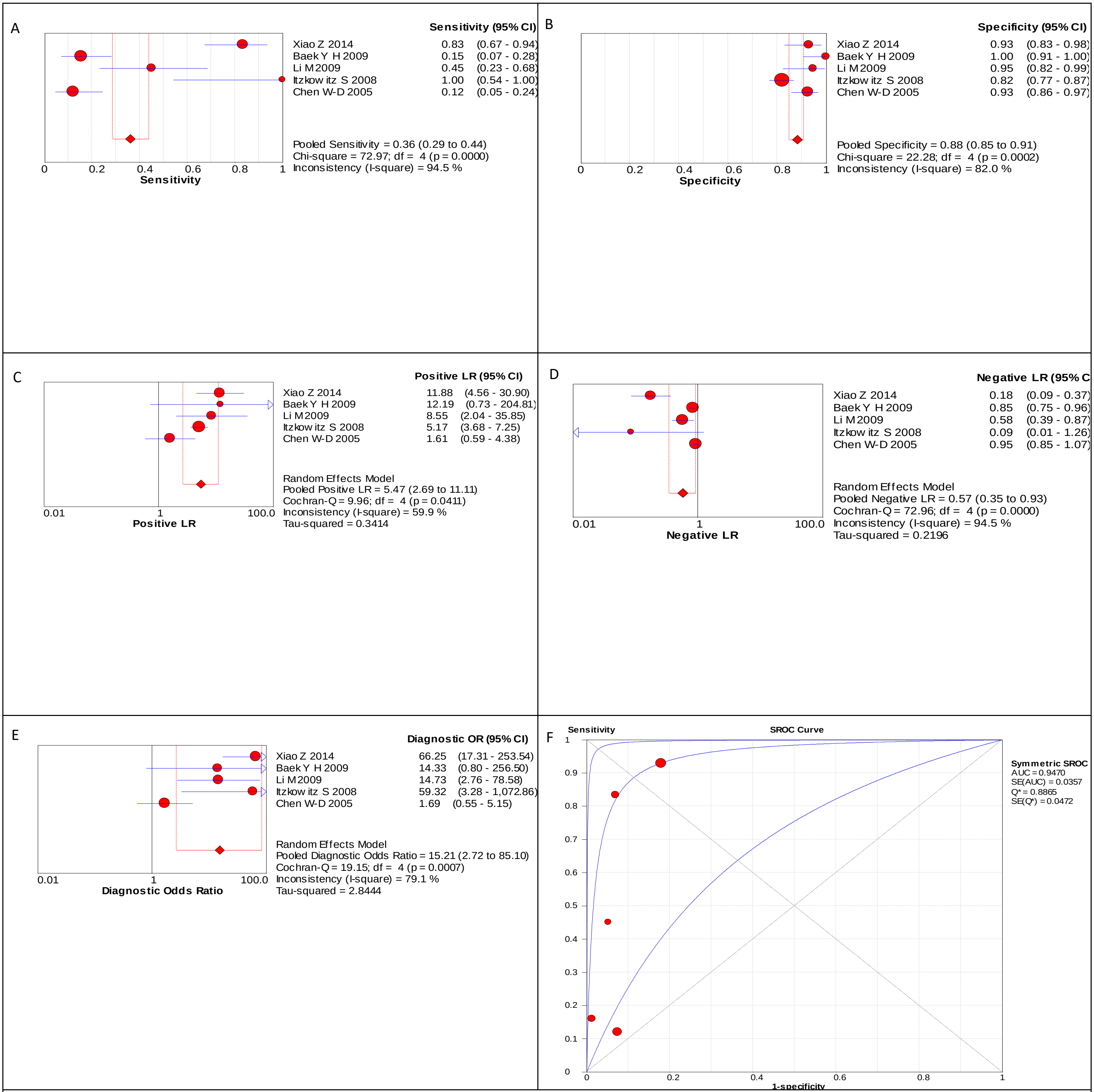

Figure S2.15. The summary *VIM of* gene in TA (A) sensitivity, (B) specificity, (C) positive likelihood ratios, (D) negative likelihood ratios, (E) diagnostic odds ratio, (F) summary ROC curves.

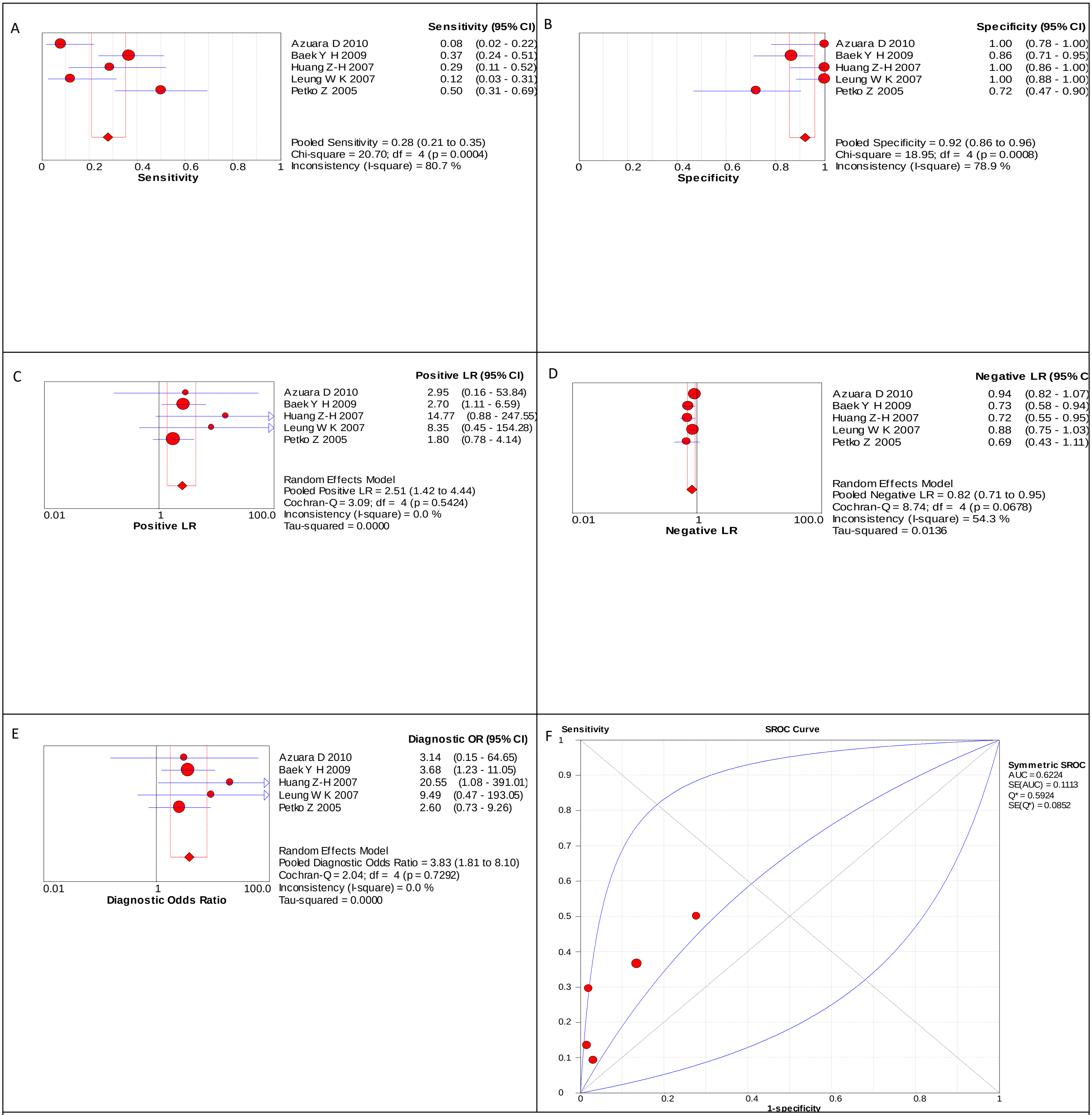

Figure S2.16. The summary *MGMT of* gene in TA (A) sensitivity, (B) specificity, (C) positive likelihood ratios, (D) negative likelihood ratios, (E) diagnostic odds ratio, (F) summary ROC curves.

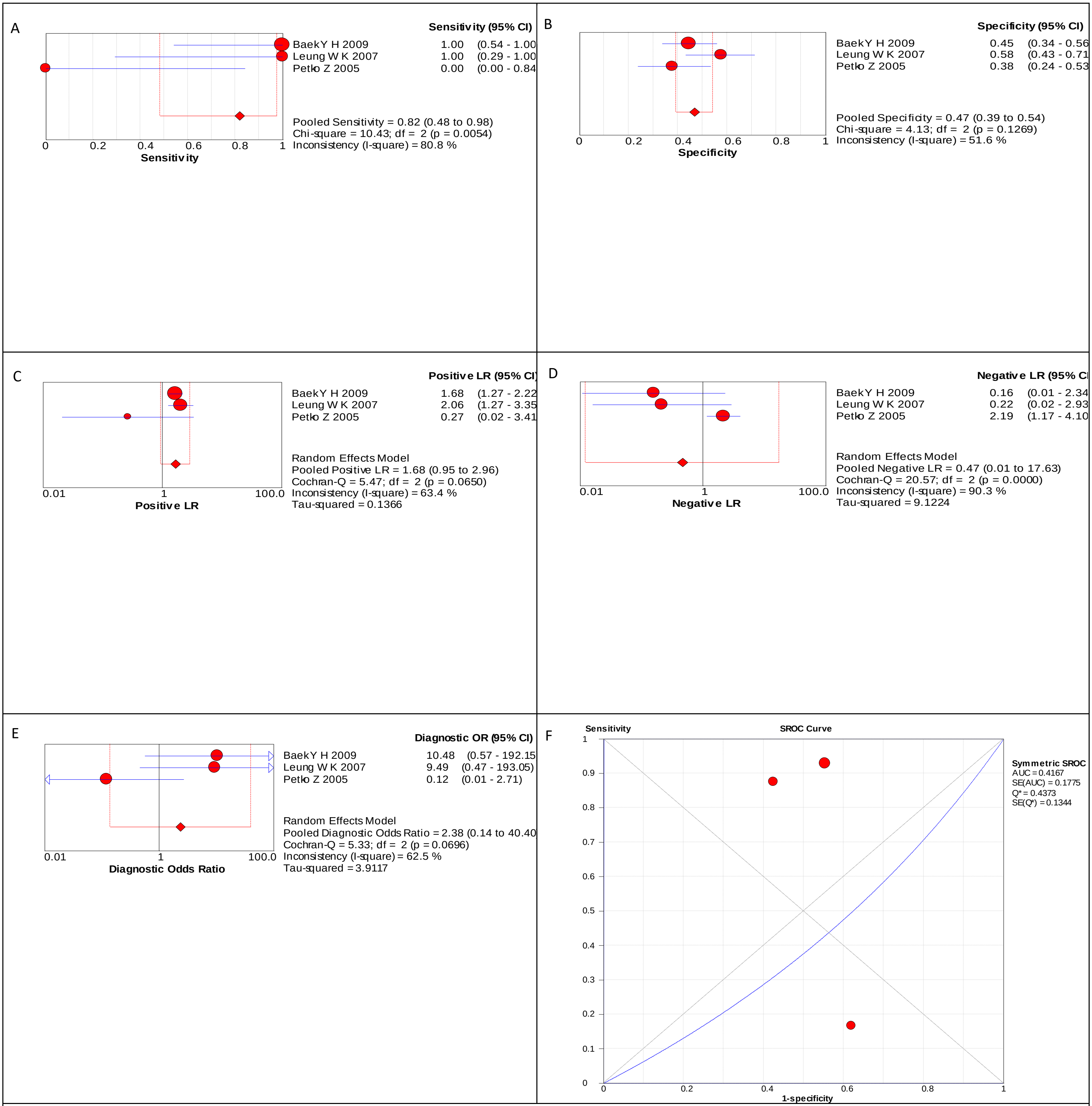

Figure S2.17. The summary *MLH1 of* gene in TA (A) sensitivity, (B) specificity, (C) positive likelihood ratios, (D) negative likelihood ratios, (E) diagnostic odds ratio, (F) summary ROC curves.

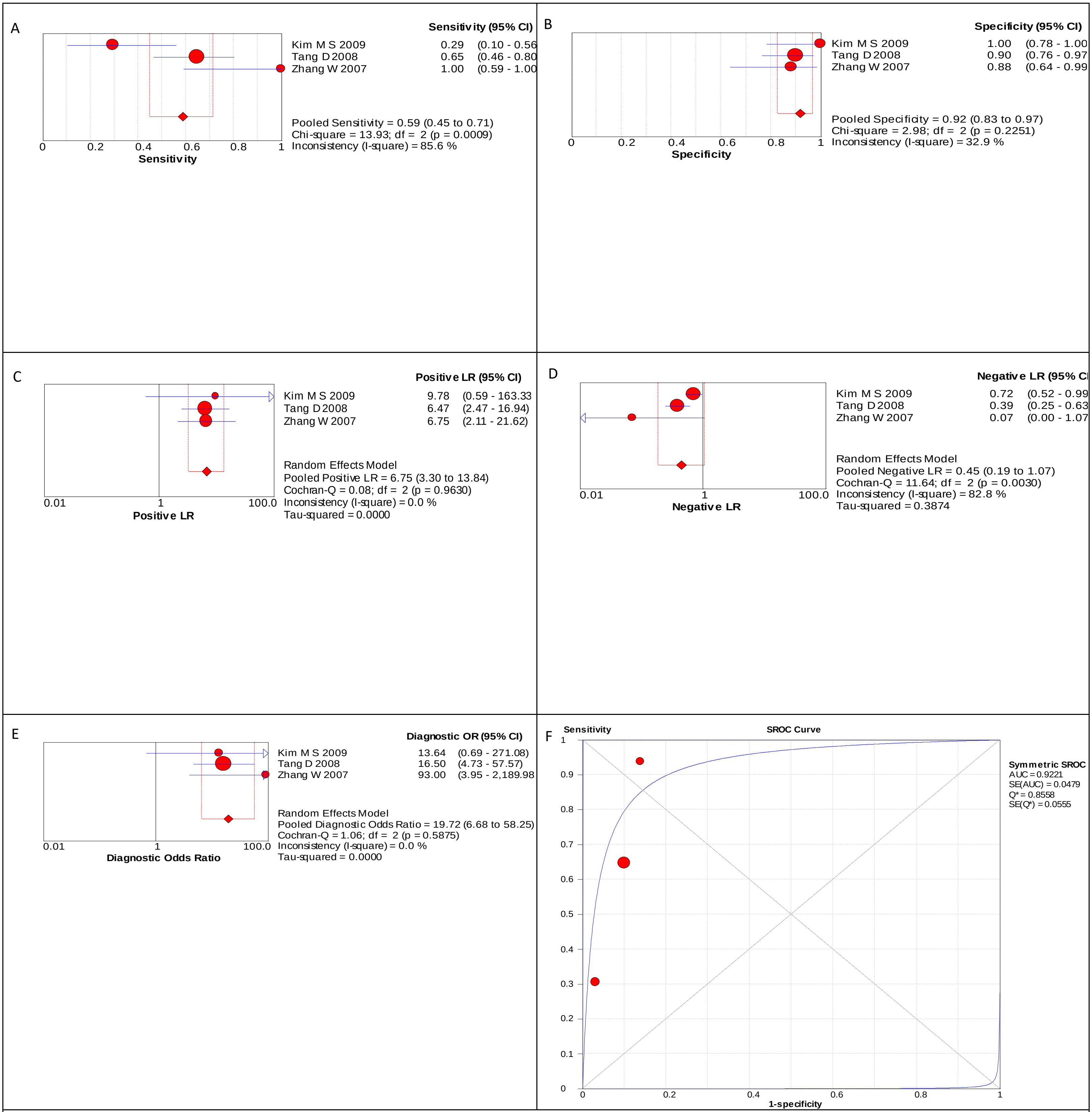

Figure S2.18. The summary *SFRP1 of* gene in TA (A) sensitivity, (B) specificity, (C) positive likelihood ratios, (D) negative likelihood ratios, (E) diagnostic odds ratio, (F) summary ROC curves.

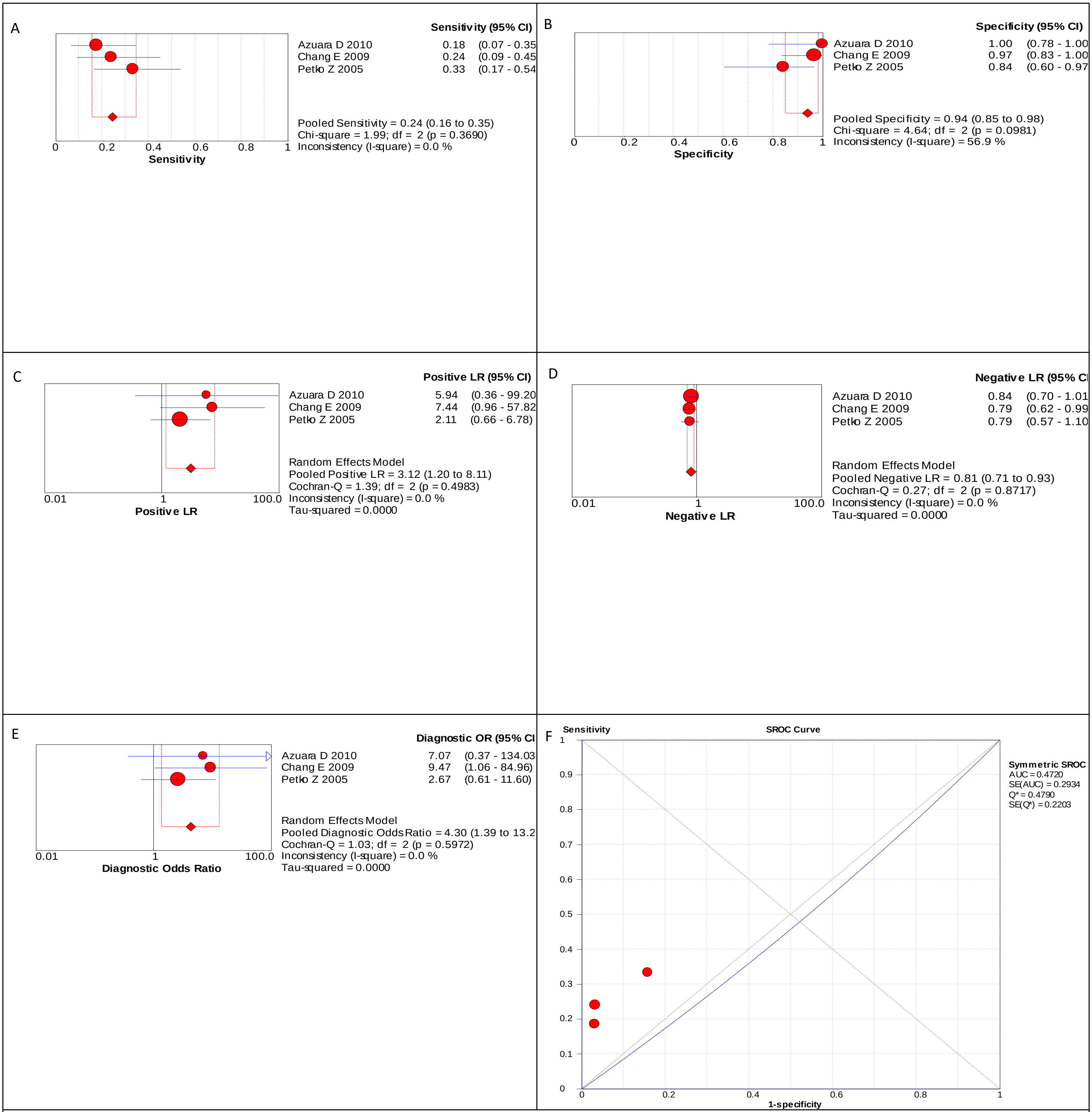

Figure S2.19. The summary *P16 of* gene in TA (A) sensitivity, (B) specificity, (C) positive likelihood ratios, (D) negative likelihood ratios, (E) diagnostic odds ratio, (F) summary ROC curves.

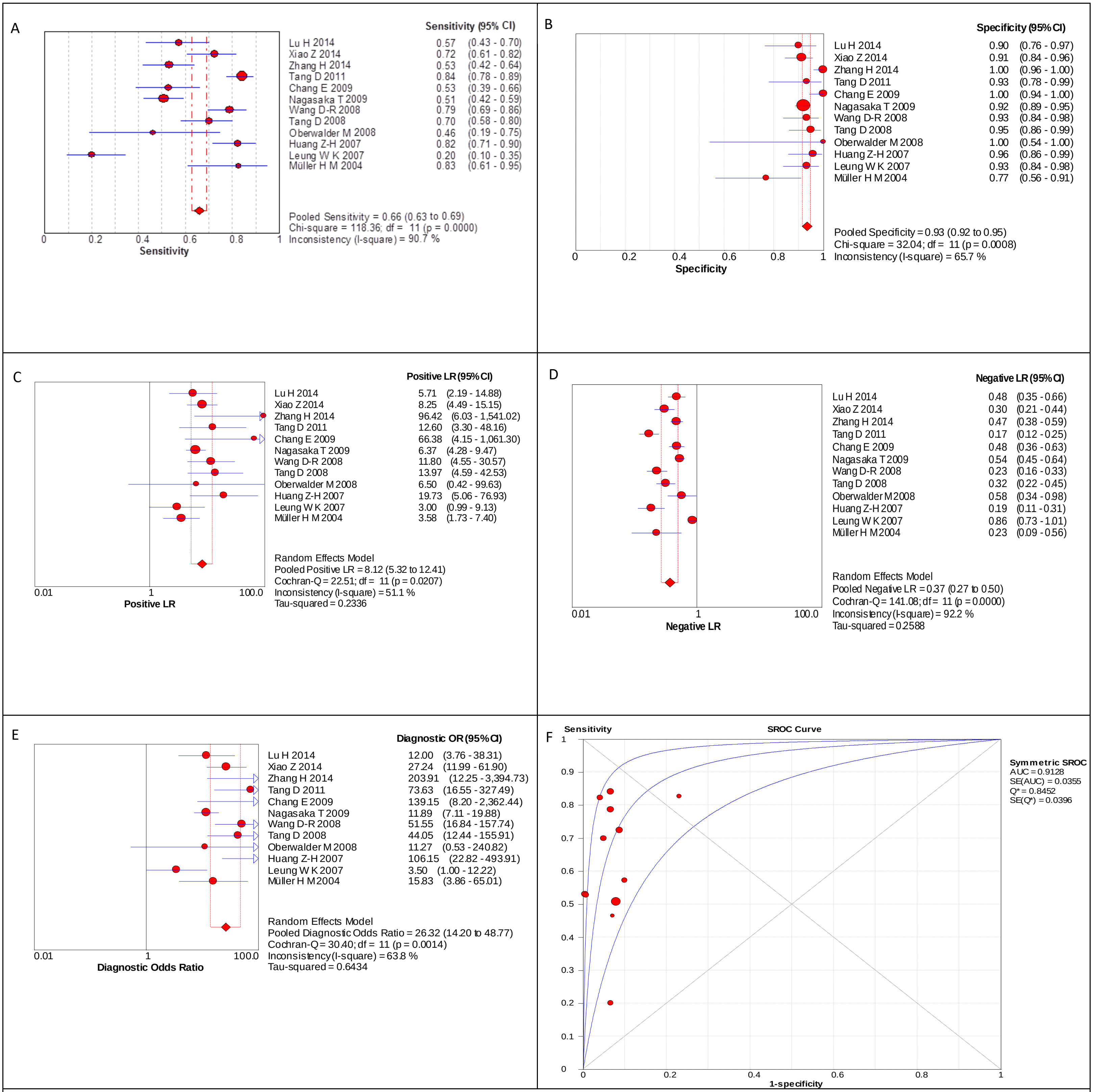

Figure S2.20. The summary *SFRP2 of* gene in TP (A) sensitivity, (B) specificity, (C) positive likelihood ratios, (D) negative likelihood ratios, (E) diagnostic odds ratio, (F) summary ROC curves.

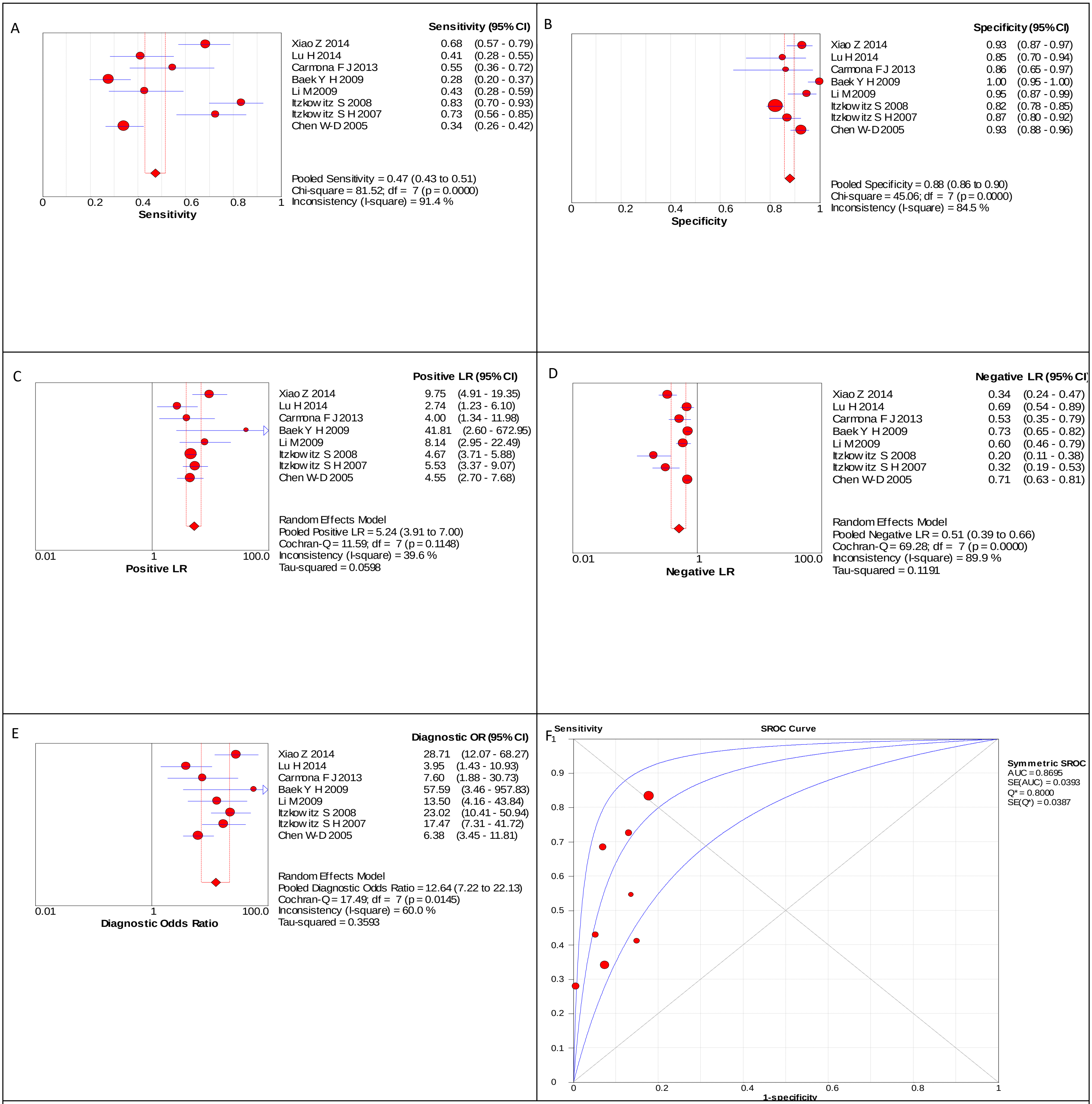

Figure S2.21. The summary *VIM of* gene in TP (A) sensitivity, (B) specificity, (C) positive likelihood ratios, (D) negative likelihood ratios, (E) diagnostic odds ratio, (F) summary ROC curves.

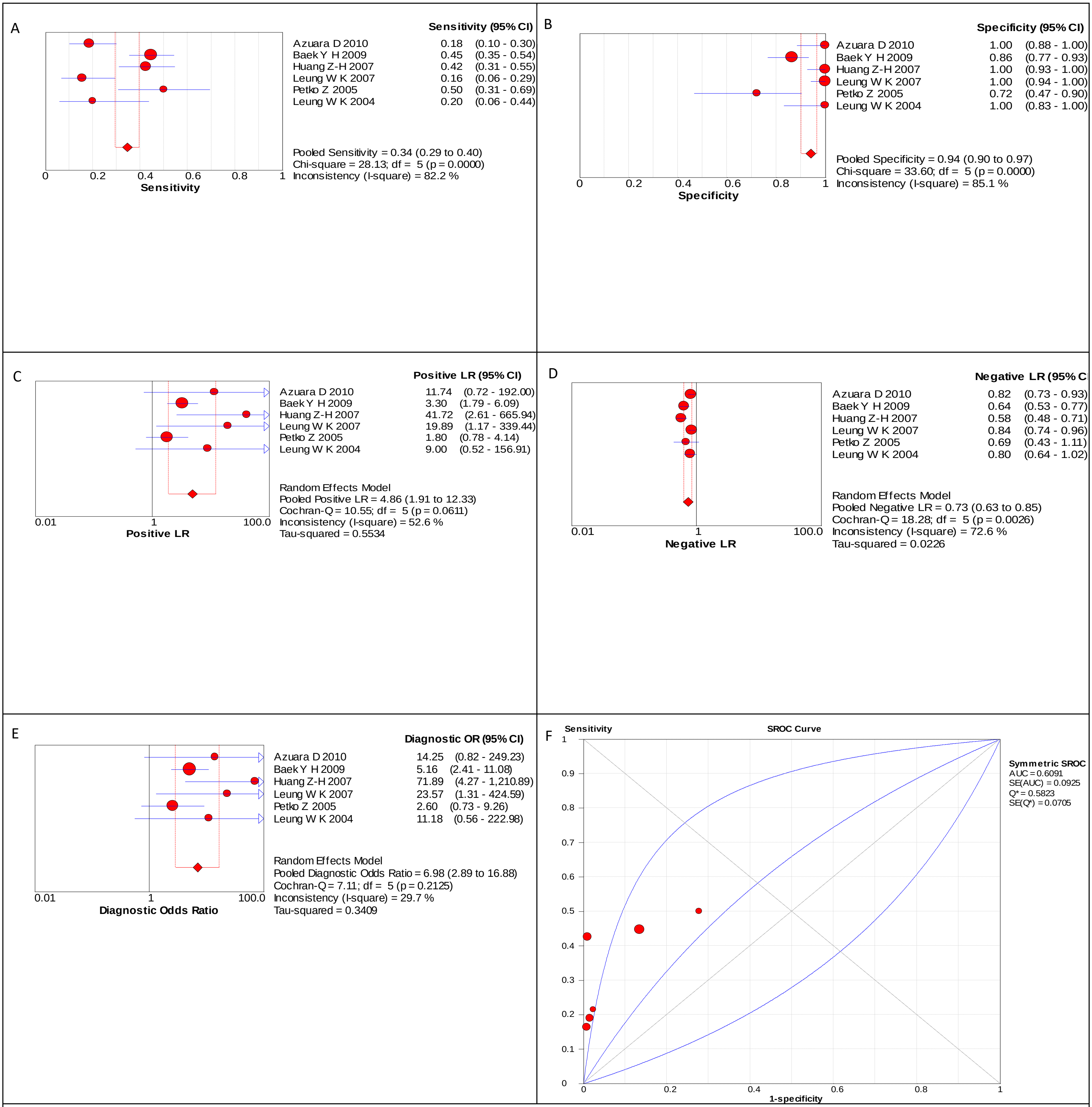

Figure S2.22. The summary *MGMT of* gene in TP (A) sensitivity, (B) specificity, (C) positive likelihood ratios, (D) negative likelihood ratios, (E) diagnostic odds ratio, (F) summary ROC curves.

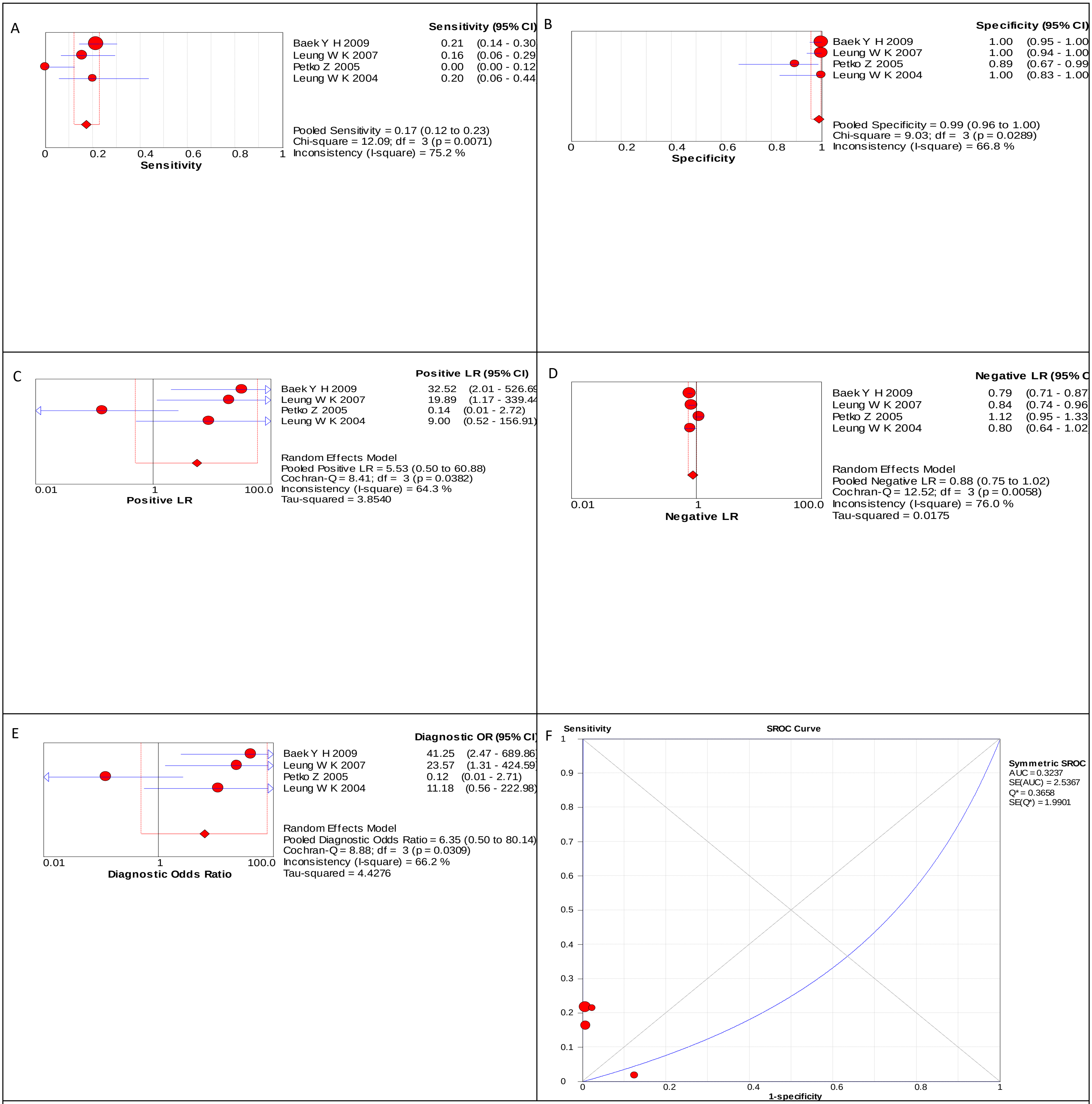

Figure S2.23. The summary *MLH1 of* gene in TP (A) sensitivity, (B) specificity, (C) positive likelihood ratios, (D) negative likelihood ratios, (E) diagnostic odds ratio, (F) summary ROC curves.

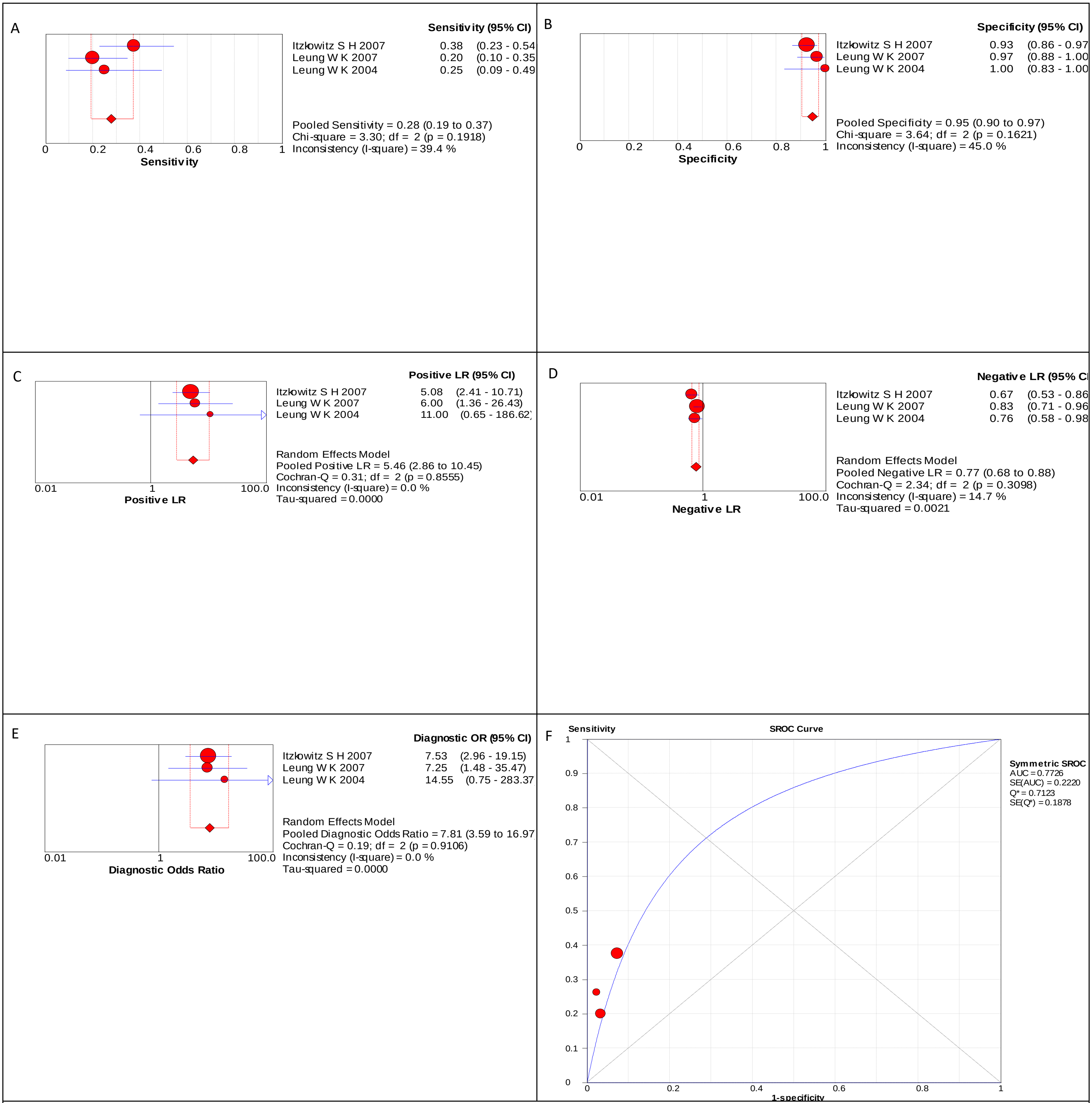

Figure S2.24. The summary *HLTF of* gene in TP (A) sensitivity, (B) specificity, (C) positive likelihood ratios, (D) negative likelihood ratios, (E) diagnostic odds ratio, (F) summary ROC curves.

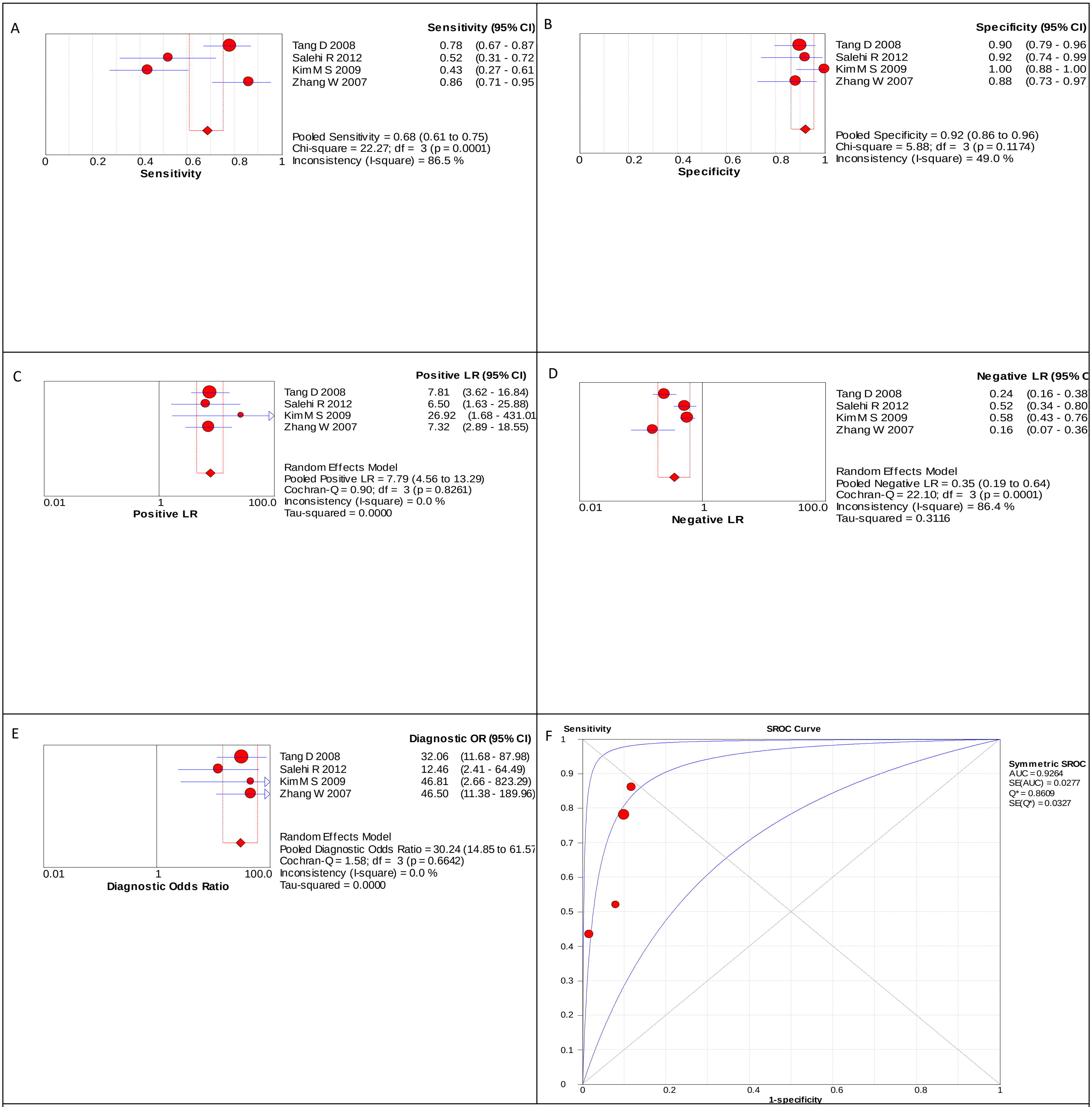

Figure S2.25. The summary *SFRP1 of* gene in TP (A) sensitivity, (B) specificity, (C) positive likelihood ratios, (D) negative likelihood ratios, (E) diagnostic odds ratio, (F) summary ROC curves.

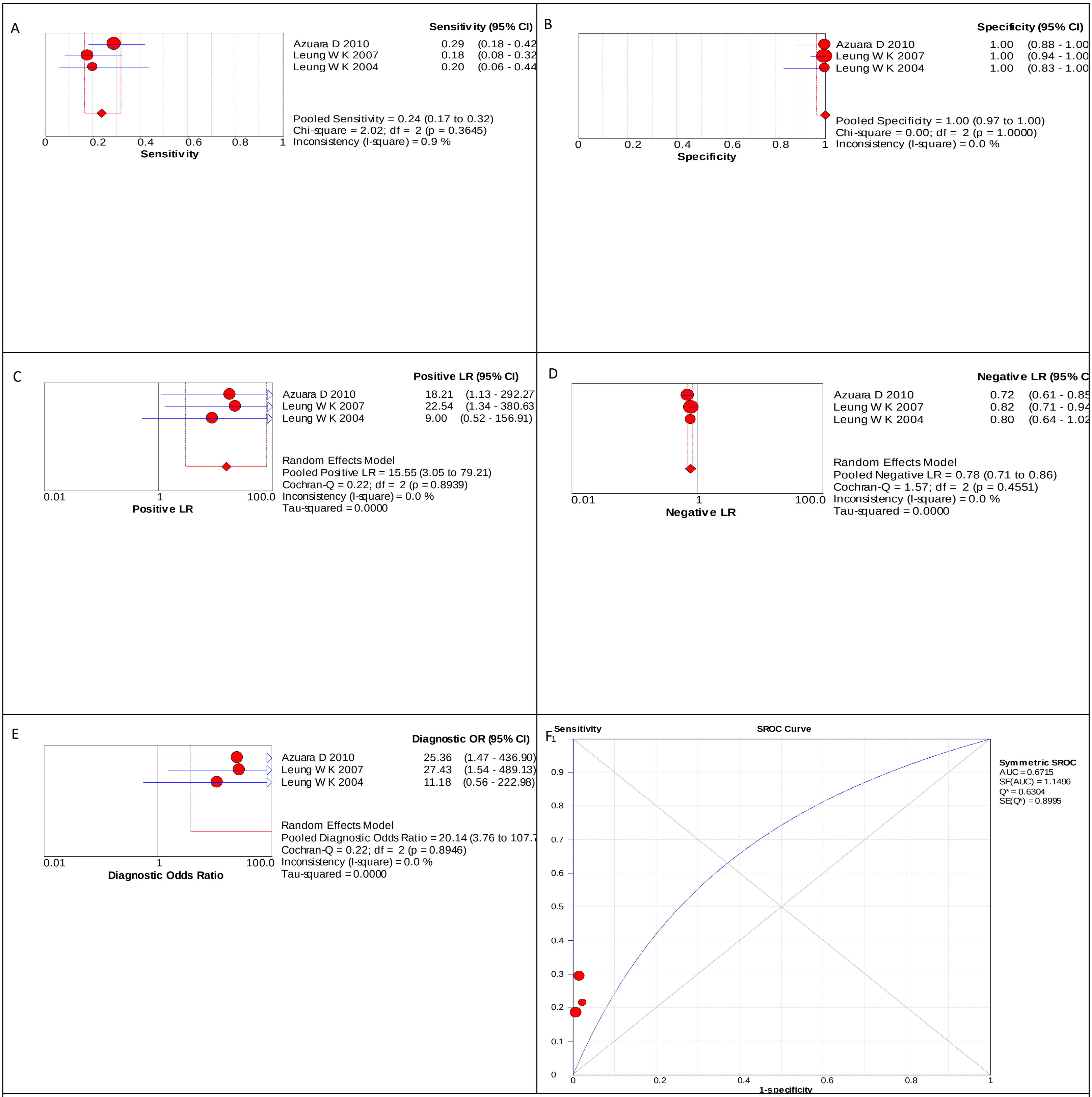

Figure S2.26. The summary *APC of* gene in TP (A) sensitivity, (B) specificity, (C) positive likelihood ratios, (D) negative likelihood ratios, (E) diagnostic odds ratio, (F) summary ROC curves.

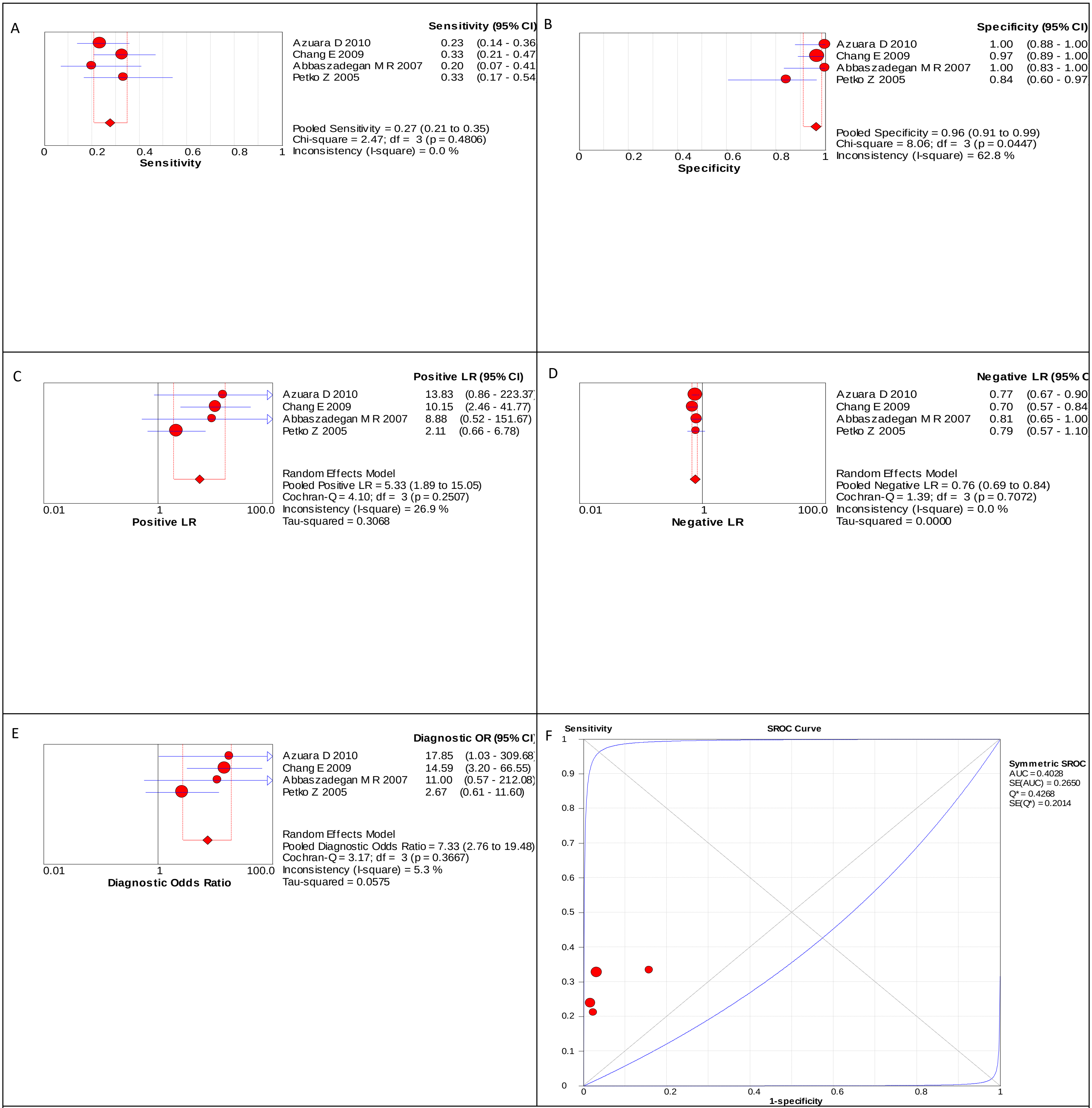

Figure S2.27. The summary *P16 of* gene in TP (A) sensitivity, (B) specificity, (C) positive likelihood ratios, (D) negative likelihood ratios, (E) diagnostic odds ratio, (F) summary ROC curves.

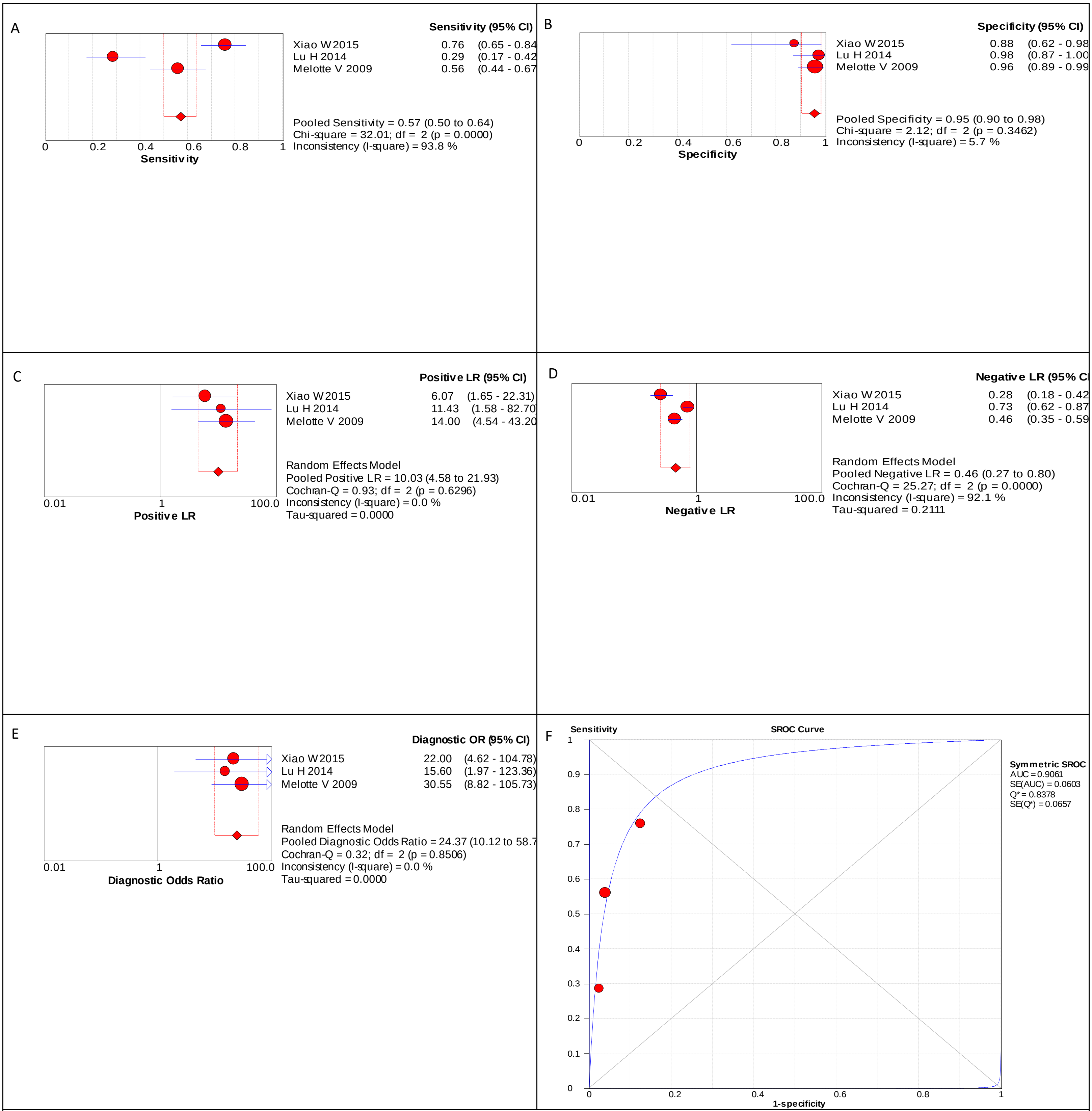

Figure S2.28. The summary *NDRG4 of* gene in TP (A) sensitivity, (B) specificity, (C) positive likelihood ratios, (D) negative likelihood ratios, (E) diagnostic odds ratio, (F) summary ROC curves.
