# Supplementary figures and images for: The importance of stool DNA methylation in colorectal cancer diagnosis: A meta-analysis
Source: PLoS One. 2018 Jul 19;13(7):e0200735. doi: 10.1371/journal.pone.0200735 (PMC6053185; doi:10.1371/journal.pone.0200735)

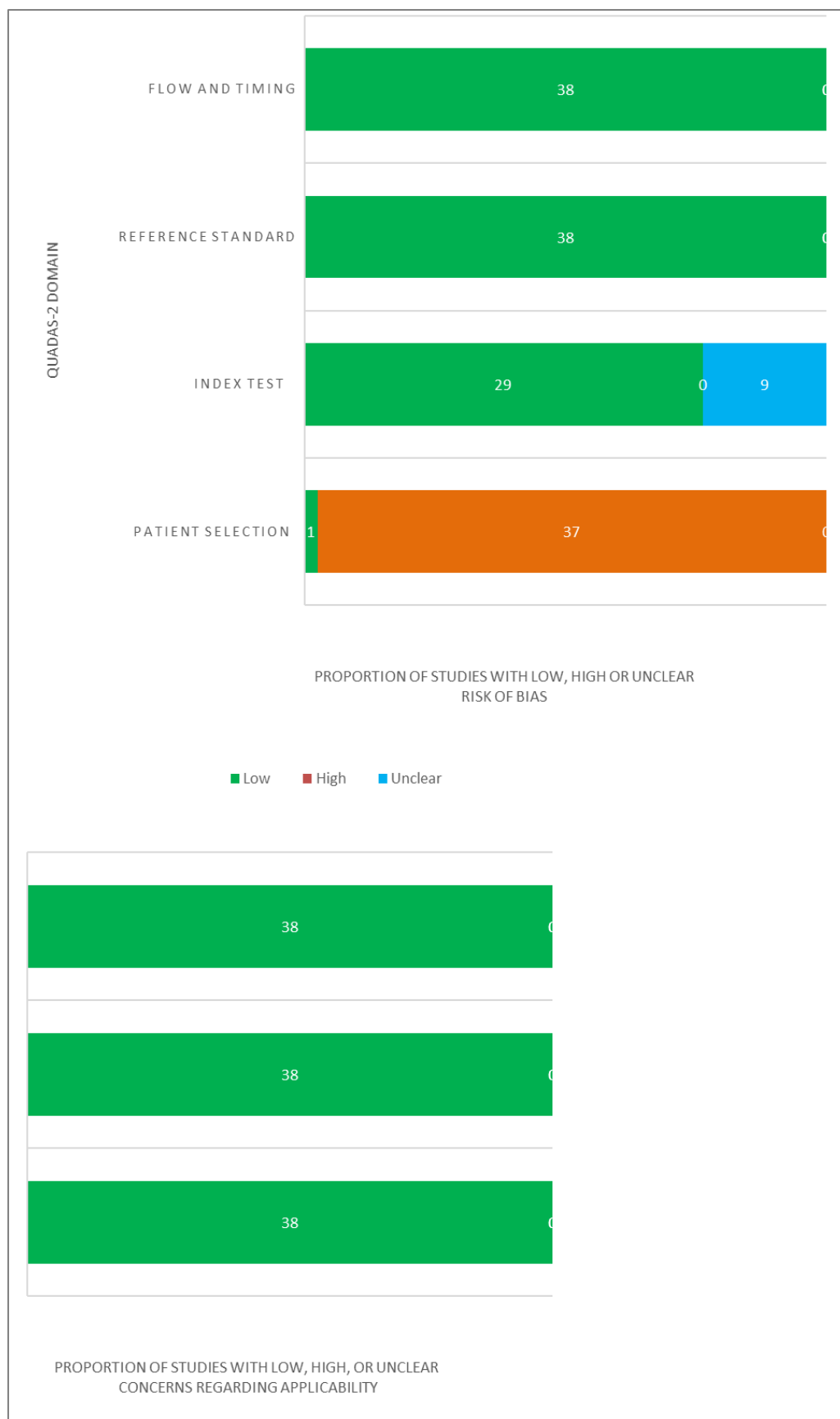

Figure 32. Graphical display of quality assessment results.

Supplement: S3 File — (PDF) [file pone.0200735.s003.pdf]
