# Supplementary material for: The importance of stool DNA methylation in colorectal cancer diagnosis: A meta-analysis
Source: PLoS One. 2018 Jul 19;13(7):e0200735. doi: 10.1371/journal.pone.0200735 (PMC6053185; doi:10.1371/journal.pone.0200735)
Supplement: S2 Table — (PDF) [file pone.0200735.s005.pdf]

Study					APPLICABILITY CONCERNS		
	PATIENT SELECTION	INDEX TEST	REFERENCE STANDARD	FLOW AND TIMING	PATIENT SELECTION	INDEX TEST	REFERE NCE STANDA RD
Müller, H. M., et al.	☹	?	☺	☺	☺	☺	☺
Leung, W. K., et al.	☹	☺	☺	☺	☺	☺	☺
Petko, Z., et al.	☹	☺	☺	☺	☺	☺	☺
Chen, W.-D., et al.	☹	☺	☺	☺	☺	☺	☺
Lenhard, K., et al.	☹	☺	☺	☺	☺	☺	☺
Huang, Z.-H., et al.	☹	☺	☺	☺	☺	☺	☺
Abbaszadegan, M. R., et al.	☹	?	☺	☺	☺	☺	☺
Zhang, W., et al.	☹	☺	☺	☺	☺	☺	☺
Itzkowitz, S. H., et al.	☹	☺	☺	☺	☺	☺	☺
Tang, D., et al.	☹	☺	☺	☺	☺	☺	☺
Leung, W. K., et al.	☹	☺	☺	☺	☺	☺	☺
Wang, D.-R. and D. Tang	☹	☺	☺	☺	☺	☺	☺
Oberwalder, M., et al.	☹	☺	☺	☺	☺	☺	☺
Itzkowitz, S., et al.	☹	☺	☺	☺	☺	☺	☺
Mayor, R., et al.	☹	?	☺	☺	☺	☺	☺
Hellebrekers, D. M., et al.	☹	☺	☺	☺	☺	☺	☺
Nagasaka, T., et al.	☹	?	☺	☺	☺	☺	☺
Melotte, V., et al.	☹	☺	☺	☺	☺	☺	☺
Li, M., et al.	☹	?	☺	☺	☺	☺	☺
Glöckner, S. C., et al.	☹	☺	☺	☺	☺	☺	☺
Kim, M. S., et al.	☹	☺	☺	☺	☺	☺	☺
Baek, Y. H., et al.	☹	☺	☺	☺	☺	☺	☺
Azuara, D., et al.	☹	☺	☺	☺	☺	☺	☺
Chang, E., et al.	☹	☺	☺	☺	☺	☺	☺

Tang, D., et al.	☹	?	☺	☺	☺	☺	☺
Salehi, R., et al.	☹	☺	☺	☺	☺	☺	☺
Bosch, L. J., et al.	☹	?	☺	☺	☺	☺	☺
Zhang, J., et al.	☹	☺	☺	☺	☺	☺	☺
Carmona, F. J., et al.	☺	☺	☺	☺	☺	☺	☺
Guo, Q., et al.	☹	☺	☺	☺	☺	☺	☺
Zhang, H., et al.	☹	☺	☺	☺	☺	☺	☺
He, C. G., et al.	☹	☺	☺	☺	☺	☺	☺
Lu, H., et al.	☹	☺	☺	☺	☺	☺	☺
Xiao, Z., et al.	☹	☺	☺	☺	☺	☺	☺
Amiot, A., et al.	☹	?	☺	☺	☺	☺	☺
Zhang, H., et al.	☹	☺	☺	☺	☺	☺	☺
Li, W.-h., et al.	☹	☺	☺	☺	☺	☺	☺
Xiao, W., et al.	☹	?	☺	☺	☺	☺	☺

☺ Low Risk ☹ High Risk ? Unclear Risk

- Quality was assessed with the Quality Assessment of Diagnostic Accuracy Studies-2 tool.
