# Supplementary material for: The importance of stool DNA methylation in colorectal cancer diagnosis: A meta-analysis
Source: PLoS One. 2018 Jul 19;13(7):e0200735. doi: 10.1371/journal.pone.0200735 (PMC6053185; doi:10.1371/journal.pone.0200735)
Supplement: S4 File — (PDF) [file pone.0200735.s007.pdf]

Threshold effect study

To detect cut-off threshold effects, the relationship between the log (sensitivity) and log (1 − specificity) were assessed using the Computation of Spearman correlation coefficient. A typical pattern of "shoulder arm" plot in an ROC space and a strong positive correlation would suggest a threshold effect.

Candidate Genes	spearman correlation values	P-Value	Cochran Q	P-Value	I^2%
**CRC**					
**SFRP2**	0.056	0.862	19.64	0.05	44
**SFRP1**	0.600	0.400	2.27	0.51	0
**NDRG4**	1	0	0.32	0.85	0
**TA**					
**SFRP1**	1	0	1.06	0.58	0
**VIM**	0.4	0.50	19.15	0.0007	79.1
**SFRP2**	0.6	0.88	13.55	0.059	48.3
**TP**					
**SFRP1**	1	0	1.58	0.66	0
**SFRP2**	0.120	0.711	30	0.0014	63.8
**NDRG4**	1	0	0.32	0.85	0
**VIM**	0.54	0.16	17.49	0.014	60
**Performance of single-gene stool-based DNA methylation biomarker tests**					
**CRC**	0.50	0.66	0.73	0.32	6.6
**Adenoma**	0.13	0.39	56.11	0.012	35.5
**CRC + Adenoma**	0.47	0	87.9	0.097	18.1
