# Supplementary material for: The importance of stool DNA methylation in colorectal cancer diagnosis: A meta-analysis
Source: PLoS One. 2018 Jul 19;13(7):e0200735. doi: 10.1371/journal.pone.0200735 (PMC6053185; doi:10.1371/journal.pone.0200735)
Supplement: S4 Table — (PDF) [file pone.0200735.s008.pdf]

Meta-analysis on Genetic Association Studies Checklist | PLOS ONE

	Item	Section name and paragraph number within manuscript
	**Introduction**	
1	Provide a detailed justification for the polymorphism studied; if a single polymorphism was analyzed, give details as to why others were not included in the meta-analysis.	N/A
2	Provide a detailed justification for the population(s) and clinical condition studied.	page 3
	**Methods**	
3	Provide full details of the search strategy employed; outline the full electronic search strategy –specific combination of keywords and any limits applied- for at least one database. Specify whether synonyms of polymorphisms/genes (e.g. SNP number) were searched.	page 3,4<br>Literature search strategy / Study Selection and Data Extraction
4	Report full details on the inclusion and exclusion criteria applied for selecting studies. *Please list the excluded articles and the reasons for exclusion of each article in a supplementary file.*	page 4<br>Inclusion and exclusion criteria
5	Provide details on how the quality of the studies included in the analyses was assessed.	page 4<br>Quality assessment<br>S2Table, S3 File
6	Describe steps taken to contact study authors to identify additional studies and to request missing data.	page 3,4<br>Literature search strategy
7	Describe how environmental effects were adjusted for, if this adjustment was not conducted, outline the reasons for this.	N/A
8	Describe the methods of handling heterogeneity/between-study variance.	page 5<br>Statistical analysis
9	Describe how the Hardy-Weinberg equilibrium and linkage disequilibrium were assessed.	N/A
10	Describe and justify the choice of model for the analyses (per-allele vs per-genotype vs genetic model-free, random effects vs fixed effects).	page 5<br>Statistical analysis

11	Describe whether a sensitivity analysis has been completed.	page 5<br>Statistical analysis
12	Describe whether an assessment of the effects of population stratification has been conducted.	N/A
13	Describe whether study-specific results have been assessed and if so the reasons for this (e.g. forest plot).	S1 File, F2 File
	**Results**	
14	Include flow diagram for the studies included in the meta-analysis as the first figure for the manuscript	page 5<br>Study selection
15	Report details on allele/genotype prevalence.	N/A
16	Report the effect size estimates and p values for each analysis.	S1 File, F2 File
	**Discussion**	
17	Discuss the limitations of the meta-analysis, including genotyping errors/bias and publication bias.	page 14
18	If the meta-analysis identifies an association within a subgroup of the population studied but not another, discuss the implications of these results, and if applicable the possibility of subgroup-specific publication bias.	N/A
19	Discuss the suitability of the sample size employed to the research question and the power of the study.	N/A
